# Constructing a Pan-Cancer Prognostic Model via Machine Learning Based on Immunogenic Cell Death Genes and Identifying NT5E as a Biomarker in Head and Neck Cancer

**DOI:** 10.3390/cimb47100812

**Published:** 2025-10-01

**Authors:** Luojin Wu, Qing Sun, Atsushi Kitani, Xiaorong Zhou, Liming Mao, Mengmeng Sang

**Affiliations:** 1 Department of Immunology, School of Medicine, Nantong University, Nantong 226019, China; 2 Mucosal Immunity Section, Laboratory of Clinical Immunology and Microbiology, National Institute of Allergy and Infectious Diseases, National Institutes of Health, Bethesda, MD 20892, USA; 3 Jiangsu Province Key Laboratory in University for Inflammation and Molecular Drug Target, Nantong University, Nantong 226019, China; 4 Basic Medical Research Center, School of Medicine, Nantong University, Nantong 226019, China

**Keywords:** pan-cancer, immunogenic cell death (ICD), machine learning algorithms, immunotherapy response, drug prediction and molecular docking

## Abstract

Immunogenic cell death (ICD) is a specialized form of cell death that triggers antitumor immune responses. In tumors, ICD promotes the release of tumor-associated and tumor-specific antigens, thereby reshaping the immune microenvironment, restoring antitumor immunity, and facilitating tumor eradication. However, the regulatory mechanisms of ICD and its immunological effects vary across tumor types, and a comprehensive understanding remains limited. We systematically analyzed the expression of 34 ICD-related regulatory genes across 33 tumor types. Differential expression at the RNA, copy number variation (CNV), and DNA methylation levels was assessed in relation to clinical features. Associations between patient survival and RNA expression, CNVs, single-nucleotide variations (SNVs), and methylation were evaluated. Patients were stratified into immunological subtypes and further divided into high- and low-risk groups based on optimal prognostic models built using a machine learning framework. We explored the relationships between ICD-related genes and immune cell infiltration, stemness, heterogeneity, immune scores, immune checkpoint and regulatory genes, and subtype-specific expression patterns. Moreover, we examined the influence of immunotherapy and anticancer immune responses, applied three machine learning algorithms to identify prognostic biomarkers, and performed drug prediction and molecular docking analyses to nominate therapeutic targets. ICD-related genes were predominantly overexpressed in ESCA, GBM, KIRC, LGG, PAAD, and STAD. RNA expression of most ICD-related genes was associated with poor prognosis, while DNA methylation of these genes showed significant survival correlations in LGG and UVM. Prognostic models were successfully established for 18 cancer types, revealing intrinsic immune regulatory mechanisms of ICD-related genes. Machine learning identified several key prognostic biomarkers across cancers, among which NT5E emerged as a predictive biomarker in head and neck squamous cell carcinoma (HNSC), mediating tumor–immune interactions through multiple ligand–receptor pairs. This study provides a comprehensive view of ICD-related genes across cancers, identifies NT5E as a potential biomarker in HNSC, and highlights novel targets for predicting immunotherapy response and improving clinical outcomes in cancer patients.

## 1. Introduction

Apoptotic tumor cells have long been regarded as a non-immunogenic form of cell death. However, emerging evidence suggests that under certain conditions, apoptosis in tumor cells can become immunogenic and lead to immunogenic cell death (ICD) [1]. ICD is a regulated form of cell death that reshapes adaptive immunity within the tumor microenvironment (TME), promotes immune memory, and enables durable clinical benefits. During chemotherapy, tumors can be suppressed not only through apoptosis but also through ICD [2]. The latter stimulates antitumor immunity via the release of tumor-associated antigens (TAAs) and tumor-specific antigens (TSAs), and is characterized by increased expression of damage-associated molecular patterns (DAMPs), precursor antigens, inflammatory cytokines, and mediators [3]. Unlike conventional apoptosis, ICD is specifically defined by DAMP release. In the context of tumors or infections, dying cells can elicit strong adaptive immune responses against altered self-antigens, cancer-derived neoepitopes, or pathogen-derived antigens [4]. Thus, induction of ICD represents a promising therapeutic approach in cancer.

Recent studies have revealed that ICD is closely linked to the TME and cancer therapy [5]. Molecules released from dying cells, such as heat shock proteins, ATP, DNA, and RNA, are recognized by antigen-presenting cells (APCs), particularly dendritic cells, thereby initiating specific immune responses [6]. In addition to activating effector immune cells, ICD may also promote apoptosis of immunosuppressive cells such as regulatory T cells and mast cells [7]. Therefore, ICD plays an essential role in immune homeostasis and antitumor immunity [8]. Specific ICD-related genes have also been implicated as prognostic indicators in different cancers. For instance, LY96 and IFNGR1 were identified as prognostic genes in thyroid cancer (THCA) [9]; LY96 is strongly downregulated in osteosarcoma, where it suppresses cell proliferation, migration, and invasion [10]; and IFNGR1 has been associated with poor prognosis in metastatic prostate cancer [11]. Moreover, EIF2AK3 enrichment was observed in lower-grade gliomas (LGG), and its knockdown significantly inhibited glioma cell viability and motility [12]. HSP90AA1 has been shown to drive proliferation in bladder cancer [13]. Collectively, these findings suggest that ICD-related molecules may serve as therapeutic targets, although their precise roles and regulatory mechanisms in tumors remain incompletely understood.

With the rapid development of bioinformatics, ICD-related classifications have been increasingly applied to explore molecular mechanisms, identify disease-specific biomarkers, and guide precision therapies. For example, Wang et al. demonstrated an association between ICD-related subtypes and immunotherapy outcomes in head and neck squamous cell carcinoma (HNSC) [14]. Similar prognostic and therapeutic values of ICD-based classifications have been reported in melanoma and gastric cancer [15,16]. By analyzing transcriptomic data from public resources such as TCGA and GTEx, it is possible to establish ICD-based classification systems for predicting prognosis and drug sensitivity across cancer types. Defining ICD-related regulatory genes can provide deeper insights into their roles in cancer biology and their impact on antitumor immunity. Hence, comprehensive investigation of ICD-related molecular features, clinical implications, and their interactions with the TME is critical for advancing the understanding of ICD and optimizing therapeutic strategies. In particular, identifying novel biomarkers and therapeutic targets is essential for evaluating ICD-associated immunotherapy efficacy.

In this study, we systematically investigated the biological functions and therapeutic potential of ICD-related genes across cancers using TCGA and GTEx datasets. We analyzed the differential expression of ICD-related genes between normal and tumor tissues, and assessed their associations with clinical characteristics. Prognostic values were further evaluated based on genomic and transcriptomic profiles. Using ICD gene expression, survival data, and clinical outcomes, patients were stratified into immunological subtypes and risk groups. We also compared immune infiltration, stemness, heterogeneity, immune scores, immune regulatory genes, and immunotherapy responses among subtypes. Furthermore, we predicted potential biomarkers and candidate drugs. Our analysis identified *NT5E* as a prognostic biomarker in HNSC, revealed its regulatory role in cell–cell communication, and uncovered key ligand–receptor interactions. Collectively, these findings provide new insights into the functions of ICD-related genes and contribute to the development of improved cancer immunotherapy strategies.

## 2. Materials and Methods

### 2.1. Data Collection

Gene expression data for normal tissues were obtained from the GTEx project (https://gtexportal.org/home/). The Cancer Genome Atlas (TCGA; http://cancergenome.nih.gov, accessed on 1 December 2024) provided multi-omics datasets, including expression profiles, clinical information, and mutation data for multiple cancer types [17]. Immunohistochemistry (IHC) results of ICD-related genes in cancer patients were retrieved from the Human Protein Atlas (HPA; http://www.proteinatlas.org/, accessed on 1 December 2024) [18].

### 2.2. Data Processing

All analyses were performed in R (v4.2.3). Batch effects between GTEx and TCGA RNA-seq data were corrected using the ComBat function from the *sva* package (v3.5). Differential expression of ICD-related genes was assessed using Wilcoxon rank-sum and signed-rank tests, while correlations were calculated using the cor.test function. Single-sample gene set enrichment analysis (ssGSEA) implemented in the GSVA package (v3.5) was used to calculate ICD scores for each patient based on ICD gene expression [19].

### 2.3. CNVs, SNVs and Methylation Levels

CNVs, SNVs, and methylation data were downloaded from the GDC database (https://portal.gdc.cancer.gov/, accessed on 1 December 2024). We included missense, nonsense, insertion/deletion, splice-site, and frameshift mutations. Variant characteristics were annotated using the maftools package (v2.16.0). Survival outcomes, including overall survival (OS), progression-free survival (PFS), disease-free interval (DFI), and disease-specific survival (DSS), were obtained from TCGA and used to evaluate associations with RNA expression, CNVs, SNVs, and methylation of ICD-related genes.

### 2.4. Molecular Clustering of Multiple Cancers

Consensus clustering was performed using ConsensusClusterPlus (v1.64.0) to identify ICD-related molecular subtypes across cancers. Partitioning around medoids (PAM) clustering with 1–Pearson correlation distance was applied, with 80% resampling repeated 10 times. The optimal number of clusters was determined from the empirical cumulative distribution function (CDF) plots [20].

### 2.5. Construction of ICD Prognostic Models by Machine Learning Algorithms

A machine learning–based framework was applied to construct prognostic models using 13 algorithms, generating 801 prognostic models in total [21]. Algorithms included Artificial Neural Network (ANN), Survival Random Forest (RF), LASSO, Elastic Net (Enet), Supervised PCA, XGBoost, Stepwise Cox, Partial Least Squares–Cox (plsRcox), Gradient Boosting Decision Tree (GBDT), Ridge, Survival SVM, Nu-SVC, and LightGBM. For each cancer type with more than 100 samples, ten-fold cross-validation was performed to identify the optimal model. In this approach, the dataset was randomly partitioned into ten folds, with one-fold used as the validation set and the remaining nine folds as the training set. This process was repeated ten times, each with a different validation fold, and the average performance metrics (C-index) across all iterations were reported as the overall model performance. Risk scores were calculated for each sample, and patients were stratified into high- and low-risk groups using optimal cutoffs determined by the maxstat package (v0.7-25). Independent GEO datasets were employed to validate the prognostic models. For HNSC, validation was performed using GSE41613 [21], GSE42743 [22], and GSE65858 [22]). For LUAD, the datasets GSE37745 [23], GSE68465 [24], and GSE72094 [23] were used. For pancreatic cancer, validation was conducted with GSE21501 [25], GSE28735 [26], and GSE62452 [27].

### 2.6. Immune Infiltration Analysis

Immune cell infiltration was estimated using the CIBERSORT algorithm in the *IOBR* package (v0.99.9) [28] and the deconvo_epic algorithm (v1.1.0). ESTIMATE, immune, and stromal scores were calculated using the ESTIMATE package (v1.0.13).

### 2.7. The Stemness Features

Stemness scores were obtained following Malta et al. [29], including DNAss, EREG-METHss, DMPss, ENHss, RNAss, and EREG.EXPss. These were calculated from RNA expression and DNA methylation profiles. Associations with ICD expression and group-specific differences were then assessed.

### 2.8. Tumor Heterogeneity

Tumor mutation burden (TMB) and Mutant-Allele Tumor Heterogeneity (MATH) scores were calculated using maftools [27]. Additional heterogeneity metrics, including neoantigen load, purity, ploidy, homologous recombination deficiency (HRD), and loss of heterozygosity (LOH), were obtained following Thorsson et al. [30]. Associations with ICD expression and subgroup differences were evaluated.

### 2.9. Immunomodulatory Gene Analysis

Immunoregulatory genes, including chemokines, receptors, MHC molecules, immunoinhibitors, and immunostimulators, were collected from Hu et al. [31]. Correlations between ICD-related genes and immunomodulatory genes were assessed, and subgroup differences were analyzed.

### 2.10. Immunotherapy Response Analysis

Immunotherapy response was predicted using the Tumor Immune Dysfunction and Exclusion (TIDE) database (http://tide.dfci.harvard.edu/, accessed on 1 December 2024) based on RNA expression profiles, and differences were analyzed between subgroups defined by the ICD risk models.

### 2.11. Anticancer Immune Response Analysis

The Tracking Tumor Immunophenotype (TIP) database (http://biocc.hrbmu.edu.cn/TIP/, accessed on 1 December 2024) was used to evaluate anticancer immune responses between high- and low-risk groups [32].

### 2.12. Identification of ICD Biomarkers by Three Machine Learning Algorithms

Potential ICD biomarkers were identified using three machine learning algorithms: LASSO, support vector machine–recursive feature elimination (SVM–RFE) [33], and random forest (RF) [34]. SVM was applied to train models based on ICD gene expression, and SVM–RFE was used for feature selection [35]. Genes with RF importance scores > 0.25 or SVM–RFE importance scores > 0.25 were considered significant. Final biomarkers were defined as the intersection of results from the three methods.

### 2.13. Single-Cell RNA-Seq Analysis

Single-cell RNA-seq data (GSE181919) from 20 primary HNSC patients were re-analyzed [36]. Seurat (v4.4.0) [37] and harmony (v1.2.1) [38] were used for preprocessing and batch correction. Cell types were annotated using raw metadata. Cells were divided into NT5E-high and NT5E-low groups based on NT5E expression. Differentially expressed genes (DEGs) were identified with FindMarker (*p* < 0.05; |log2FC| > 0.584). AUCell (v1.22.0) [39] was used to calculate ICD-related scores, and subgroup comparisons were performed within each cell type. CellChat (v1.6.1) [40] was applied to infer cell–cell communication networks.

### 2.14. Drug Prediction and Molecular Docking Analysis

Potential therapeutic compounds were identified using the Connectivity Map (CMAP; https://clue.io/, accessed on 1 December 2024) [41] based on differential ICD-related gene expression in HNSC. Protein structures of ICD-related genes were downloaded from UniProt (https://www.uniprot.org/, accessed on 1 December 2024) [42], and chemical structures of candidate compounds from PubChem (https://pubchem.ncbi.nlm.nih.gov/, accessed on 1 December 2024) [43]. Structures were preprocessed with UCSF Chimera (v1.17.3) [33]. Docking simulations were performed using DOCK (v6.10) [44], and compound–target interactions were visualized with PyMol (v2.4.0) [45].

### 2.15. Statistical Analysis

All statistical analyses were conducted in R (v4.2.3). Two-tailed tests were applied throughout, and *p* < 0.05 was considered statistically significant unless otherwise specified.

## 3. Results

### 3.1. Impact of ICD-Related Genes on Prognosis Across Cancer Types

**Figure 1:**
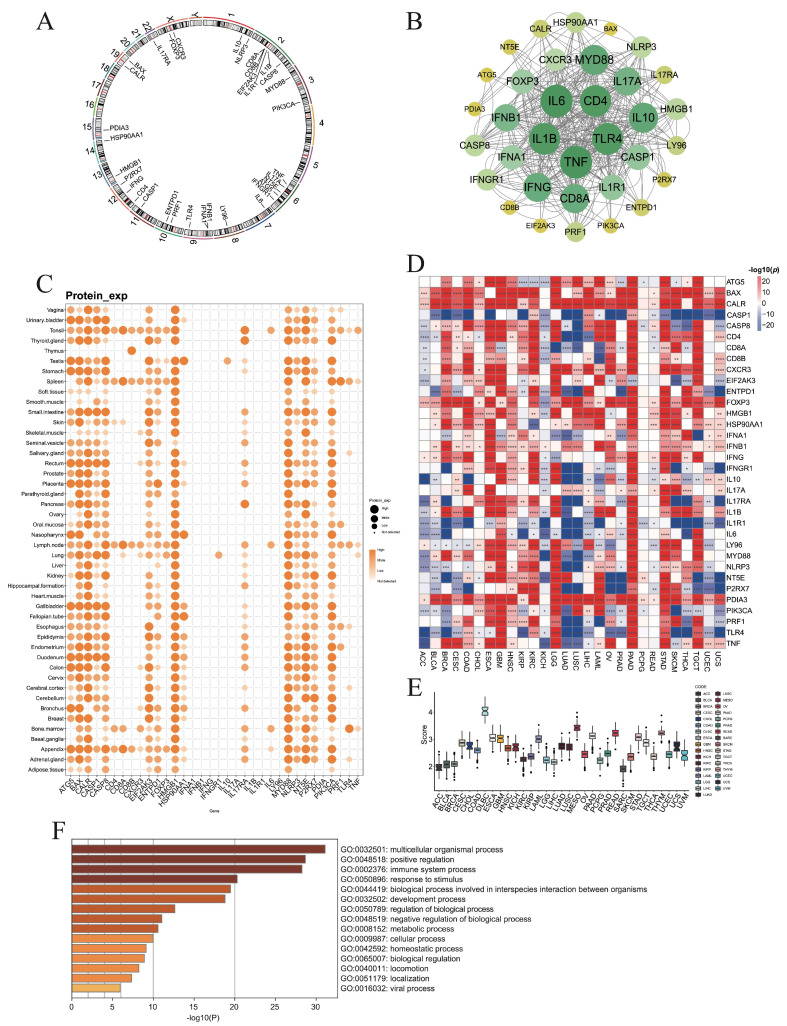
Expression levels of immunogenic cell death associated genes (ICDs). (**A**) Chromosome circular plot showing the distribution of 34 ICD-related genes on the chromosomes. (**B**) The protein–protein interactions (PPIs) of ICD-related genes. (**C**) The protein levels of ICD genes in various tissues. (**D**) The differential RNA expression levels of ICD genes between normal and tumor tissues in each cancer type. (**E**) The ICD scores of each cancer type by ssGSVA algorithm. (**F**) Gene ontology analysis identified multiple pathways associated with ICD-associated genes (*: *p* < 0.05, **: *p* < 0.01, ***: *p* < 0.001, ****: *p* < 0.0001).

To assess the prognostic and therapeutic potential of ICD-related genes in pan-cancer, we first curated 34 regulatory molecules from Garg et al. [42] and analyzed their RNA-seq expression profiles using TCGA and GTEx datasets. Genomic mapping showed that these genes were distributed across most autosomes but absent from chromosomes 4, 5, 16–18, 20–21, and Y (Figure 1A). Protein–protein interaction (PPI) analysis was subsequently performed to investigate the interactions among these genes within biological systems, identifying CD4, TLR4, TNF, IL1B, and IL6 as hub proteins (Figure 1B).

At the protein level, *BAX*, *CASP8*, *HMGB1*, *MYD88*, and *PIK3CA* were ubiquitously expressed across normal tissues except the thymus, while *CD8B* expression was largely restricted to the thymus (Figure 1C). Comparative transcriptomic analysis demonstrated that ICD genes were upregulated in tumor tissues of ESCA, GBM, KIRC, LGG, PAAD, and STAD, but downregulated in LUAD and LUSC (Figure 1D). Notably, *CASP1* was consistently elevated in normal tissues across most cancers, whereas *FOXP3* and *PDIA3* were frequently enriched in tumors (Figure 1D).

Furthermore, ssGSVA analysis revealed the highest ICD scores in DLBC (Figure 1E). GO enrichment highlighted biological processes related to multicellular organization, positive regulation of biological processes, and immune system functions (Figure 1F).

### 3.2. Connection of ICD-Related Gene Levels with Clinical Survival

**Figure 2:**
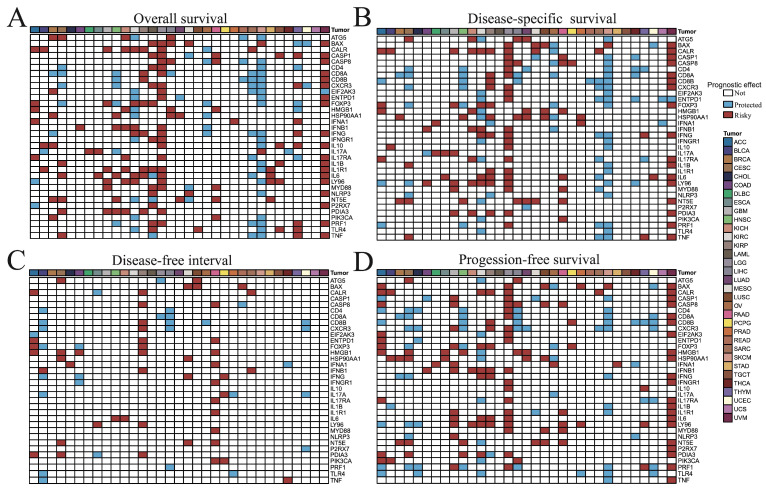
Correlation analysis between ICD-related genes and (**A**) overall survival, (**B**) disease-specific survival, (**C**) disease-free interval, and (**D**) progression-free survival of 33 tumor patients. The *p* value less than or equal to 0.05 and hazard ratio (HR) greater than 1 indicated that this gene was a risk factor (color in red), while HR less than 1 indicated that this gene was a protected factor (color in blue), and the *p* value greater than 0.05 indicated that this gene was not significant factor for survival.

To assess the prognostic impact of ICD-related genes across cancers, we evaluated their associations with OS, DSS, DFI, and PFS. For OS and DSS, most ICD-related genes acted as risk factors in LGG and UVM, but were predominantly protective in SKCM (Figure 2A,B). For DFI, ICD-related genes tended to be risk factors in KIRP and PAAD, while protective in BLCA (Figure 2C). For PFS, they again served mainly as risk factors in LGG and UVM and as protective factors in SKCM (Figure 2D). Collectively, these results indicate that ICD-associated genes confer unfavorable prognosis in LGG and UVM, but favorable outcomes in SKCM.

### 3.3. Inter-Gene Relationships Among ICD Regulators

We first examined the linear correlations among ICD-related genes across 33 cancer types and observed particularly strong associations in KICH and UVM. We next assessed correlations between ICD-related genes and ICD scores (Supplementary Figure S1). Notably, *CASP1*, *CASP8*, *CD4*, *CD8B*, *CXCR3*, and *LY96* were strongly correlated with ICD scores in LGG (Supplementary Figure S1). Overall, most ICD-related genes showed positive correlations in tumors, consistent with the notion that ICD involves the coordinated release of inflammatory signals and the induction of immune cell apoptosis, thereby promoting synergistic molecular expression and anti-infective or anti-tumor immune responses. In contrast, the limited number of negative correlations observed may suggest that specific forms of cell death dominate within certain tumor contexts, a possibility that warrants further investigation.

### 3.4. ICD Expression Versus Clinicopathologic Features

We related ICD gene expression to age, grade, grade, and TNM stage. Using 60 years as the age cutoff, KIRP and LGG showed the most age-related differences, with *HSP90AA1* and *HMGB1* frequently age-associated (Supplementary Figure S2A). Gender-biased expression was most pronounced in KIRP (Supplementary Figure S2B). By grade, *ENTPD1* and *IFNG* showed broad effects, with many genes differing in KIRC and LGG (Supplementary Figure S2C). Stage-wise analyses identified widespread differences across M, N, and T categories, including *BAX* and *LY96* (Supplementary Figure S2D–G).

### 3.5. Genomic Alterations of ICD Genes

Genetic variation plays a critical role in tumorigenesis; therefore, we investigated the impact of CNVs, SNVs, and DNA methylation of ICD-related genes across cancers. CNV analysis revealed that *ATG5* showed the strongest positive correlation with RNA expression in BRCA (Supplementary Figure S3A). Survival analyses indicated that CNVs of ICD-related genes were significantly associated with OS, PFS, DSS, and DFI in UCEC and KIRP (Supplementary Figure S3B). At the gene set level, CNVs were linked to DFI differences in CESC, DSS differences in LUSC, KIRP, DLBC, and BLCA, OS differences in THYM, BLCA, and UVM, and PFS differences in SARC and UVM (Supplementary Figure S3C).

In terms of SNVs, *PIK3CA* exhibited the highest mutation frequencies in UCEC, COAD, and BRCA (Supplementary Figure S3D). Only *BAX* and *CD8A* mutations were significantly associated with DSS, OS, and PFS in BRCA (Supplementary Figure S3E). Gene set–based comparisons revealed survival differences between wild-type and mutant groups in UCEC (OS, PFS, DFI), STAD (OS, PFS, DSS), and BLCA (OS, PFS, DSS, DFI) (Supplementary Figure S3F).

Methylation analysis showed widespread differences between tumor and normal tissues, particularly in BRCA, PRAD, KIRP, COAD, BLCA, UCEC, HNSC, LUAD, LUSC, LIHC, and KIRC (Supplementary Figure S3G). Notably, *NT5E* was hypermethylated in BRCA tumors, whereas *P2RX7* was enriched in normal KIRC tissues. Methylation of *PRF1* was inversely correlated with RNA expression across most cancers, while the majority of genes in THCA also displayed negative correlations (Supplementary Figure S3H). Importantly, methylation status was most strongly associated with survival in LGG (DSS, OS, and PFS), followed by UVM (Supplementary Figure S3I).

Together, these findings indicated that CNV, SNV, and methylation of ICD-related genes exert profound influences on gene expression and patient survival, with particularly strong effects in LGG and UVM.

### 3.6. Cluster Analysis

To stratify tumor samples by ICD-related gene expression, we performed unsupervised consensus clustering separately for each cancer type, which divided patients into two or three subclusters (Supplementary Figure S4A). Differential expression analysis revealed that *HSP90AA1* varied across subclusters in most cancers, while the largest number of ICD-related genes showed subgroup differences in BLCA and THYM (Supplementary Figure S4B). Moreover, ICD scores significantly differed among subclusters in BLCA, BRCA, KIRP, KIRC, LGG, LUAD, STAD, and THCA (Supplementary Figure S4C).

### 3.7. Machine-Learning Prognostic Models

**Figure 3:**
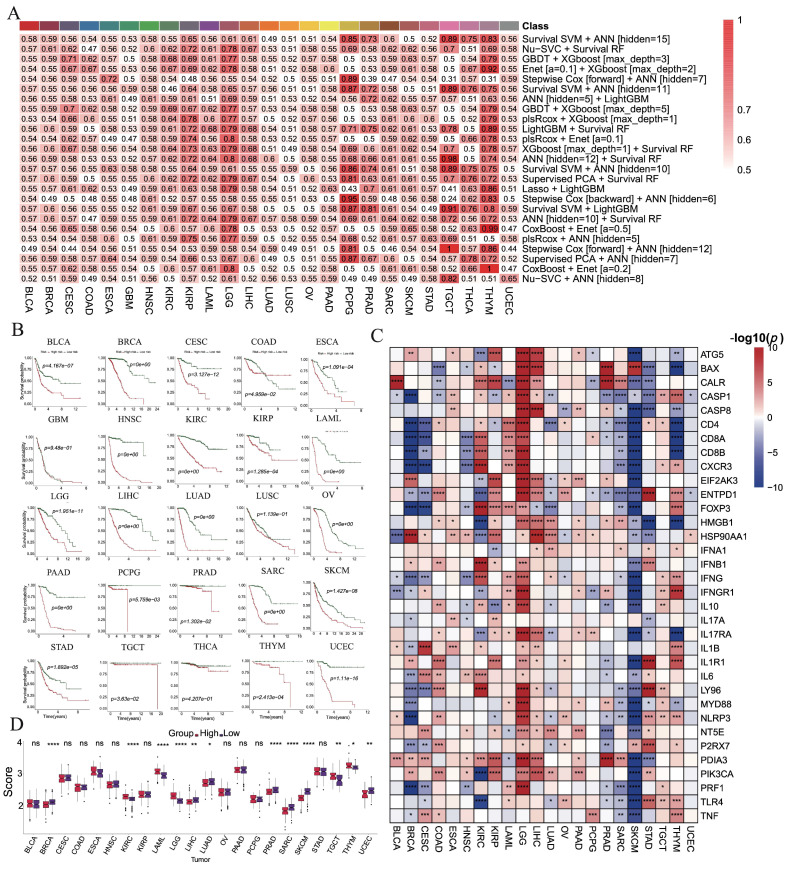
The construction of prognostic model of ICD-related genes in each cancer based on machine learning algorithms. (**A**) The optimal prognostic models are constructed by machine learning algorithms for each cancer type. (**B**) Kaplan–Meier analyses for OS between the high-risk and low-risk groups. (**C**) The differential expressions of ICD-related genes between two subgroups. (**D**) The difference in ICD score between two subgroups. (ns: *p* > 0.05, *: *p* < 0.05, **: *p* < 0.01, ***: *p* < 0.001, ****: *p* < 0.0001).

We constructed 801 prognostic models across 25 cancer types and evaluated their performance using C-index values (Supplementary Figure S5). Optimal models for each cancer type were identified through 10-fold cross-validation based on validation C-index values, with model selection varying across cancers (Figure 3A). For instance, the optimal model for BLCA combined survival SVM with ANN [hidden = 15], for BRCA it was Nu-SVC with survival RF, and for CESC it was GBDT with XGBoost [max_depth = 3].

Patients were stratified into high- and low-risk groups according to median risk scores (Figure 3B). Significant survival differences were observed in 22 cancers (except GBM, LUSC, and THCA), with the low-risk group consistently exhibiting better outcomes. ROC analysis further confirmed robust predictive power, with AUCs exceeding 0.7 in most cancers and reaching >0.9 in BRCA, LAML, LIHC, LUAD, PCPG, SARC, and THYM (Supplementary Figure S6). In contrast, BLCA, SKCM, and STAD exhibited AUCs below 0.7, indicating limited predictive power (Supplementary Figure S6).

Differential expression analyses revealed that ICD-related genes were generally upregulated in high-risk groups of LGG but downregulated in SKCM (Figure 3C). ICD scores also differed significantly between risk groups in BRCA, KIRC, LAML, LGG, LIHC, LUAD, PRAD, SARC, SKCM, TGCT, THYM, and UCEC, with higher scores in high-risk groups of BRCA, LIHC, LUAD, PRAD, SARC, SKCM, and UCEC (Figure 3D).

**Figure 4:**
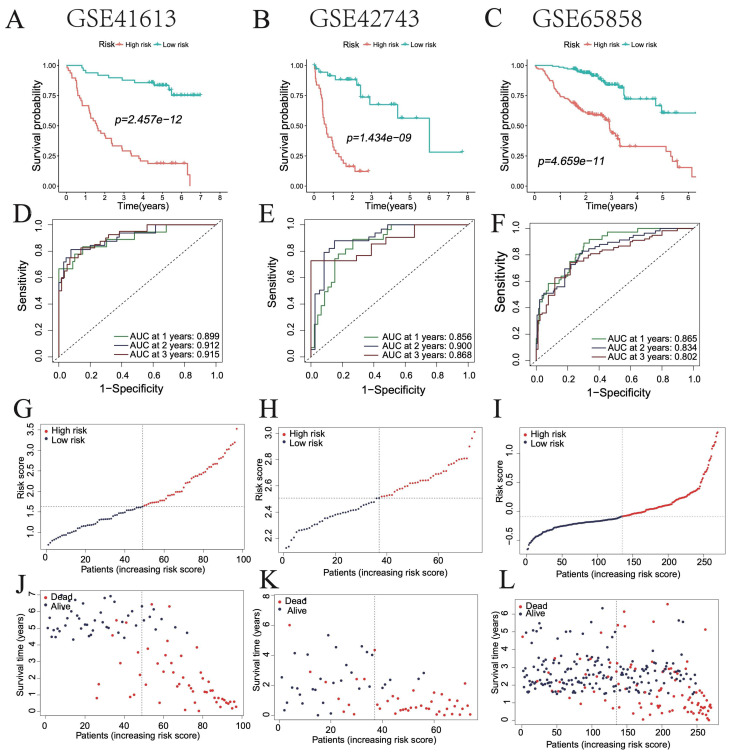
Validation of the optimal prognostic model in HNSC patients. (**A**–**C**) Survival curves of patients in high- and low-risk groups in GSE41613, GSE42743, and GSE65858 databases. (**D**–**F**) ROC curve showing the AUC of the model for different survival times in GSE41613, GSE42743, and GSE65858 databases. (**G**–**L**) Distribution of risk scores and survival statuses in high- and low-risk groups.

To further validate model robustness, three independent HNSC datasets (GSE41613, GSE42743, and GSE65858) were analyzed. Kaplan–Meier curves showed significantly better survival in low-risk groups across all datasets (Figure 4A–C). ROC curves yielded AUCs of 0.899, 0.912, and 0.915 for GSE41613; 0.856, 0.900, and 0.868 for GSE42743; and 0.865, 0.834, and 0.802 for GSE65858, all exceeding 0.8 (Figure 4D–F). Consistent differences in risk scores and clinical outcomes further supported the prognostic efficacy of the model (Figure 4G–L).

Similarly, the model was validated in three independent LUAD datasets (GSE37745, GSE68465, and GSE72094). ROC analyses produced AUCs of 0.684, 0.662, and 0.667, respectively (Supplementary Figure S7A–C), and Kaplan–Meier curves demonstrated significantly better survival in the low-risk groups across all datasets (Supplementary Figure S7D–F). In addition, validation in three independent PAAD datasets (GSE21501, GSE28735, and GSE62452) yielded AUCs of 0.899, 0.948, and 0.907, respectively (Supplementary Figure S8A–C). Kaplan–Meier analyses again confirmed significantly improved survival in the low-risk groups across all datasets (Supplementary Figure S8D–F).

### 3.8. Immune Infiltration, Stemness Features, and Tumor Heterogeneities Analysis

**Figure 5:**
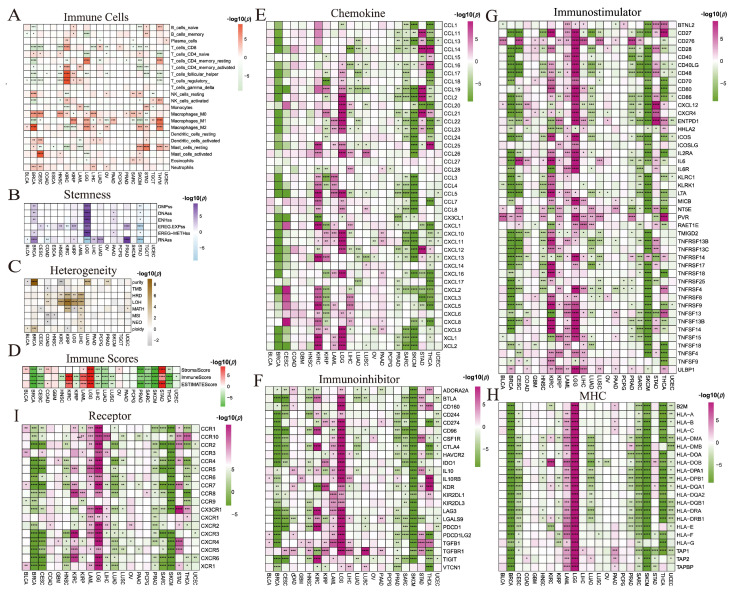
The differential expression of (**A**) immune cell infiltrations, (**B**) stemness features, (**C**) heterogeneities, (**D**) immune infiltration scores, (**E**) chemokine, (**F**) immunoinhibitor, (**G**) immunostimulator, (**H**) MHC, and (**I**) receptor genes between subclusters and lasso groups in each cancer type (*: *p* < 0.05, **: *p* < 0.01, ***: *p* < 0.001, ****: *p* < 0.0001).

To investigate the immune functions of ICD-associated genes in cancer, we first assessed their correlations with immune cell infiltration across 33 tumor types. Most ICD-related molecules were positively correlated with memory resting CD4 T cells in PRAD and TGCT, and with CD8 T cells in UVM (Supplementary Figure S9). Immune cell distributions differed significantly among subclusters, particularly in KIRC, LUAD, and THYM, with memory resting CD4 T cells, regulatory T cells, and M2 macrophages showing the most pronounced differences (Supplementary Figure S18A). Notably, most immune cells displayed higher infiltration in the low-risk group of BRCA, while M0 macrophages predominated in the high-risk group and CD8 T cells were enriched in the low-risk group across multiple cancers (Figure 5A).

We next examined the relationship between stemness and ICD-related genes. RNAss was negatively correlated with most ICD molecules in KICH, LGG, and THCA (Supplementary Figure S10). ATG5, CASP8, and HSP90AA1 were positively associated with stemness in TGCT, whereas CALR, HMGB1, IL1R1, MYD88, NLRP3, and NT5E were negatively associated. By contrast, CALR and MYD88 were positively correlated with stemness in ACC. Significant stemness differences were observed among subclusters in HNSC, KIRP, LGG, LUAD, and TGCT (Supplementary Figure S18B), and between high- and low-risk groups in BRCA, LGG and STAD (Figure 5B).

We further explored tumor heterogeneity. Tumor purity was negatively correlated with most ICD genes in BRCA, CHOL, COAD, KICH, KIRC, LIHC, and LUAD, while HRD was positively correlated with most genes in KICH (Supplementary Figure S11). HSP90AA1 showed broad positive associations with heterogeneity in HNSC. Subclusters in HNSC, LUSC, and UCEC exhibited significant heterogeneity differences (Supplementary Figure S18C), and HRD and LOH were markedly elevated in the high-risk groups of COAD, KIRC, KIRP, LGG, and LIHC (Figure 5C).

Finally, most ICD-related molecules were positively correlated with stromal, immune, and ESTIMATE scores across cancers (Supplementary Figure S12). These scores also differed significantly among subclusters (Supplementary Figure S18D), being elevated in high-risk groups of LAML, LGG, and STAD, but enriched in low-risk groups of BRCA, CESC, LIHC, LUAD, PRAD, SARC, SKCM, and THCA (Figure 5D).

### 3.9. Immunomodulatory Gene Landscape

We next examined the associations between ICD-related and immunomodulatory genes, including chemokines, receptors, MHC, immunoinhibitors, and immunostimulators. Overall, most ICD-related genes were positively correlated with immunomodulatory genes across cancers, particularly with MHC molecules (Supplementary Figures S13–S17). For instance, CD8A expression correlated strongly with chemokine genes (Supplementary Figure S13). Chemokines such as *CCL2*, *CCL23*, *CCL25*, *CCL26*, *CCL4*, *CCL5*, *CXCL10*, *CXCL11*, *CXCL3*, and *CXCL5* were differentially expressed across subclusters in more than 15 cancer types, with marked differences observed in BRCA, LUAD, TGCT, and THCA (Supplementary Figure S18E). Most chemokine genes were downregulated in high-risk groups of BRCA and SKCM but upregulated in KIRC and LGG (Figure 5E).

*CASP1* showed broad positive correlations with immunoinhibitory genes (Supplementary Figure S14). More than 20 immunoinhibitory genes were differentially expressed among subclusters in BRCA, LGG, and SKCM, with *LGALS9* differing in over 15 cancer types (Supplementary Figure S18F). Most immunoinhibitors were enriched in the low-risk groups of BRCA, CESC, HNSC, PRAD, SARC, and SKCM, while high expression was seen in the high-risk groups of LAML and LGG (Figure 5F).

Similarly, *CASP1* correlated positively with immunostimulatory genes in TGCT and THCA (Supplementary Figure S15). Strong subcluster-specific differences in immunostimulatory genes were observed in BLCA, BRCA, KIRC, LGG, LIHC, LUAD, PRAD, STAD, and THYM, with *LTA* and *TNFRSF4* differing in more than 20 cancer types (Supplementary Figure S18G). Most immunostimulatory genes were enriched in low-risk groups of BRCA, CESC, and SKCM, but in high-risk groups of KIRC, LAML, and LGG (Figure 5G).

*CASP1*, *CD4*, *CD8A*, *CXCR3*, and *IFNG* were positively correlated with most ICD genes (Supplementary Figure S16). All MHC genes showed strong differences among subclusters in BLCA, BRCA, CESC, LGG, LUAD, and LUSC (Supplementary Figure S18H). Most MHC genes were highly expressed in the low-risk groups of BRCA, CESC, SARC, SKCM, THCA, and UCEC, but enriched in high-risk groups of LAML and LGG (Figure 5H).

Finally, *CD4* correlated positively with most receptor genes (Supplementary Figure S17). Receptor genes in LIHC, LUSC, PRAD, STAD, and THYM exhibited significant subcluster differences, with *CCR10*, *CCR5*, and *CXCR6* differing in more than 15 cancer types (Supplementary Figure S18I). Most receptor genes were highly expressed in the low-risk groups of BRCA, CESC, LUAD, PRAD, SARC, and THYM, while enriched in the high-risk groups of LAML and LGG (Figure 5I).

### 3.10. Immunotherapy Analysis

**Figure 6:**
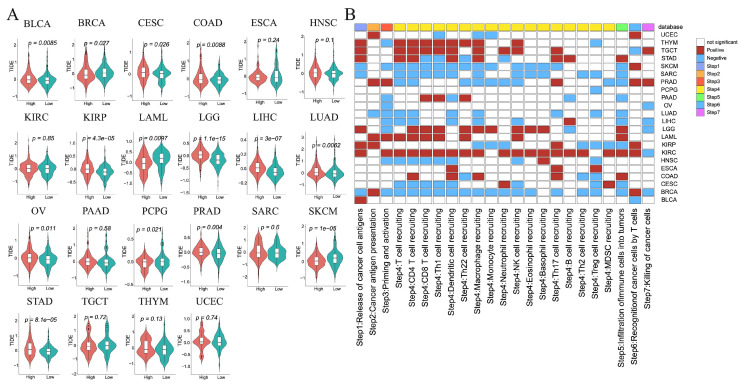
Immunotherapy and anticancer immune response analysis between high- and low-risk groups in each tumor. (**A**) The differences in TIDE scores. (**B**) The differences in activity scores of the cancer-immunity cycles.

The TIDE score reflects tumor immune escape potential and resistance to immunotherapy. We calculated TIDE scores for each patient and compared differences between high- and low-risk groups across cancer types. High-risk tumors exhibited significantly higher TIDE scores in BLCA, CESC, COAD, KIRP, LGG, LIHC, LUAD, OV, and STAD, but lower scores in BRCA, LAML, PCPG, and SKCM (Figure 6A). Elevated TIDE scores indicated reduced predicted responsiveness to immunotherapy.

### 3.11. Anticancer Immune Response Analysis

To assess the potential impact of ICD-related genes on cancer immunotherapy, we calculated cancer-immunity cycle activity scores using the TIP database. Most steps showed higher activity in high-risk tumors of KIRC, LAML, LGG, TGCT, and THYM (Figure 6B, Supplementary Figure S19), but lower activity in high-risk tumors of BRCA, CESC, SARC, and SKCM (Figure 6B, Supplementary Figure S19). Notably, step 7 (cancer cell killing) scores differed significantly between high-cold and low-hot groups in BRCA, KIRC, LGG, LIHC, OV, PRAD, and TGCT, indicating that active immune responses could enhance tumor cell elimination.

### 3.12. Biomarker Discovery Across Cancers

**Figure 7:**
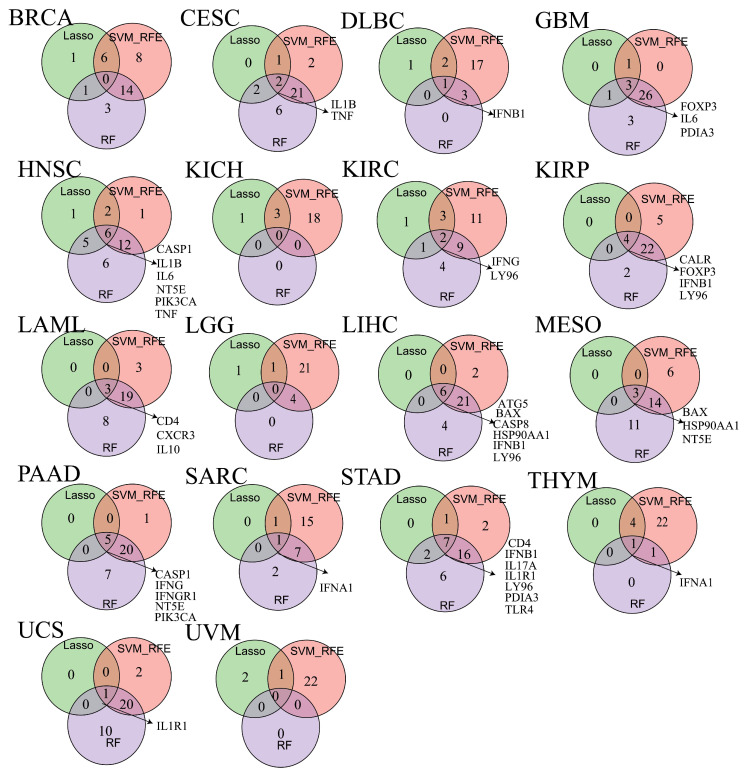
The veen plot showing biomarkers obtained from the intersection of results from SVM-RFE, RF and LASSO algorithms in overall survival (OS).

To evaluate whether ICD-related genes could serve as prognostic biomarkers across cancers, we applied three machine learning algorithms (LASSO, SVM-RFE, and RF) to identify prognosis-associated genes (Supplementary Table S1). Using OS as the endpoint, biomarkers were identified in 14 cancer types (Figure 7). For example, *IL1B* and *TNF* were identified in CESC, while IFNB1 was a biomarker in DLBC. *LY96* emerged as a recurrent biomarker in four cancers (KICH, KIRC, LIHC, and STAD), and *IFNB1* was identified in DLBC, KIRP, and STAD. Other genes were identified as biomarkers in one or two cancer types.

We further identified ICD-related biomarkers significantly associated with disease- DSS, DFI, and PFS (Supplementary Figure S20A–C). Biomarkers linked to DSS were found in eight cancer types, with *CALR*, *FOXP3*, and *NT5E* identified in at least three cancers (Supplementary Figure S20A). For DFI, biomarkers were identified in 11 cancer types, with *CASP8*, *CD8B*, *FOXP3*, *HMGB1*, *HSP90AA1*, *LY96*, *PDIA3*, and *TNF* recurrently identified across at least three cancers (Supplementary Figure S20B). For PFS, biomarkers were detected in 10 cancer types, with *CALR*, *CASP8*, *FOXP3*, and *LY96* consistently identified in at least three cancers (Supplementary Figure S20C).

### 3.13. Single-Cell Characterization of NT5E in HNSC

**Figure 8:**
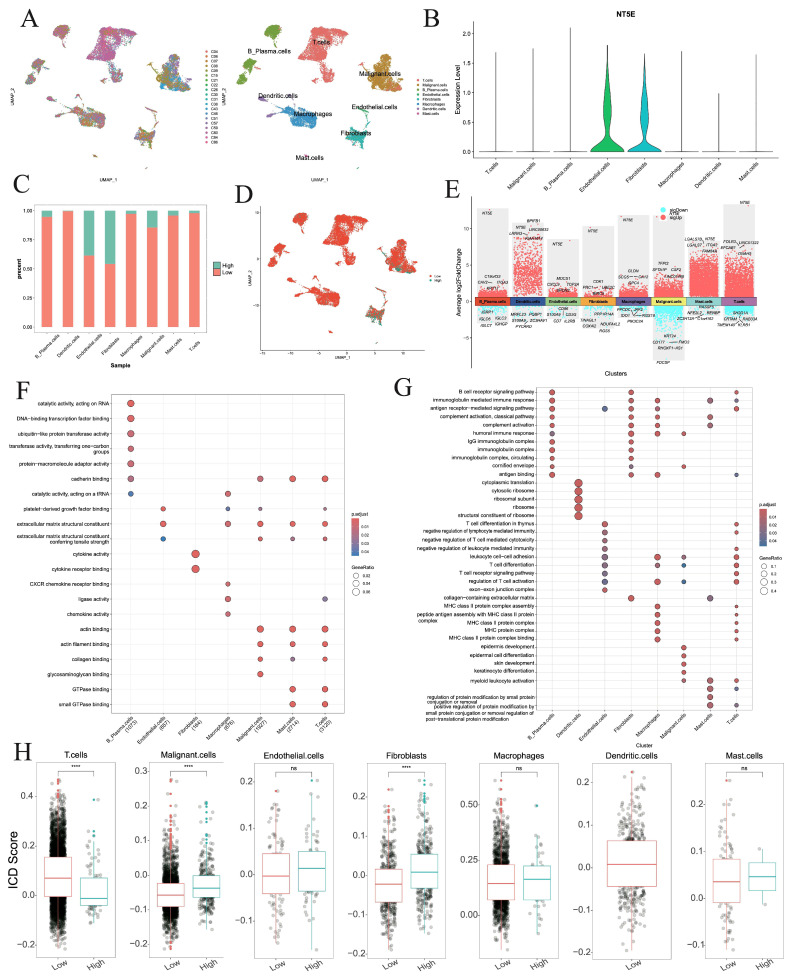
Single-cell analysis of *NT5E* in the HNSC. (**A**) UMAP plot showing the patients and cell types. (**B**) *NT5E* RNA expression in different cell types. (**C**) Percentages of activated and nonactivated cells in each cell type. (**D**) UMAP plot showing *NT5E*-high and *NT5E*-low cells on the basis of *NT5E* RNA expression. (**E**) The differentially expressed genes between *NT5E*-high and *NT5E*-low cell group in each cell type. (**F**,**G**) GO analysis of up-regulated and down-regulated genes in *NT5E*-high cell group of each cell type. (**H**) Comparison of the ICD scores between the *NT5E*-high and *NT5E*-low cell group in each cell type (ns: not significant, ****: *p* < 0.0001).

*NT5E* was identified as a risk factor for OS, DSS, and PFS in HNSC (Figure 2), and also emerged as a biomarker for OS and DSS in HNSC across three machine learning algorithms (Figure 7, Supplementary Figure S20), suggesting its potential as a therapeutic target. To further characterize *NT5E*, we analyzed single-cell RNA-seq data from 20 primary cancer patients (GSE181919). After batch correction using Harmony, eight major cell types were identified, including B plasma cells, dendritic cells, endothelial cells, fibroblasts, macrophages, malignant cells, mast cells, and T cells (Figure 8A). *NT5E* expression was highest in endothelial cells and fibroblasts (Figure 8B). Within each cell type, cells were stratified into *NT5E*-high and *NT5E*-low groups. Notably, *NT5E*-low cells accounted for >10% of endothelial cells, fibroblasts, and malignant cells (Figure 8C,D). Differential expression analysis revealed extensive transcriptional changes, with thousands of upregulated and hundreds of downregulated genes across cell types (Figure 8E, Supplementary Table S2). GO analysis highlighted cell type-specific biological processes, such as catalytic activity and RNA binding in B plasma cells, cytokine receptor binding in fibroblasts, and ribosome biogenesis in dendritic cells (Figure 8F,G, Supplementary Tables S3 and S4). ICD scores were elevated in *NT5E*-high fibroblasts and malignant cells but reduced in *NT5E*-high T cells (Figure 8H).

**Figure 9:**
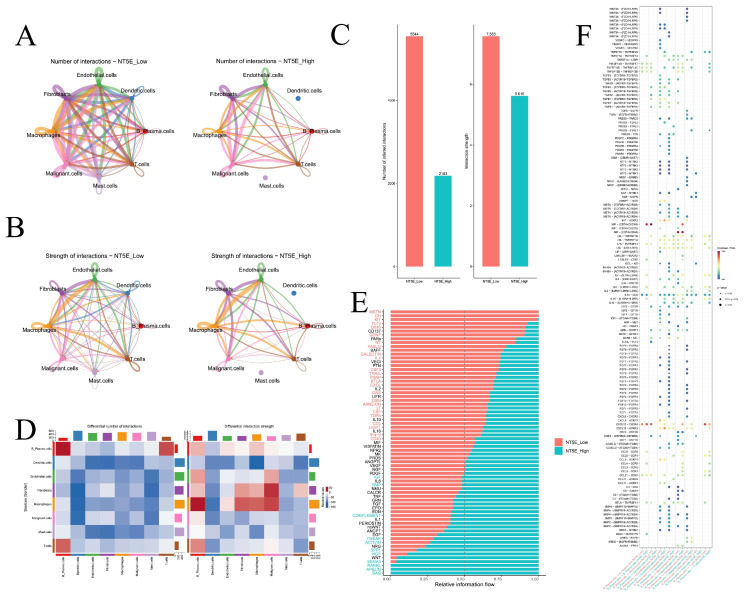
Cell–cell communication. (**A**) The number of interactions between different cell types in the *NT5E*-high and *NT5E*-low groups. (**B**) The strength of interactions between different cell types in the *NT5E*-high and *NT5E*-low groups. (**C**) The number of interactions and strength of interactions in the *NT5E*-high and *NT5E*-low groups. (**D**) The differential number of interactions and strength of interactions between the *NT5E*-high and *NT5E*-low groups. (**E**) The relative information flow in the *NT5E*-high and *NT5E*-low groups. (**F**) Comparison of specific ligand-receptor interactions among cell types between the *NT5E*-high and *NT5E*-low group.

Cell–cell communication networks inferred by CellChat revealed striking differences between *NT5E*-high and *NT5E*-low groups (Figure 9A,B). Dendritic cells and mast cells lost interactions in the *NT5E*-high group, and overall interaction numbers and strengths were reduced compared to the *NT5E*-low group (Figure 9C). In the *NT5E*-high group, B plasma cells exhibited increased self-interactions and stronger crosstalk with T cells, while macrophage interactions with fibroblasts, malignant cells, and B plasma cells were enhanced (Figure 9D). Pathway analysis indicated MSTN and GH signaling were specific to *NT5E*-low cells, whereas PANKL, APELIN, and BAG signaling were enriched in *NT5E*-high cells (Figure 9E). Ligand–receptor analysis further showed that TGFB-mediated interactions (TGFB1/2/3–ACVR1B+TGFBR2) were prevalent in NT5E-low groups, while IL2–(IL2RB+IL2RG) and IL15–(IL15RA+IL2RB) signaling dominated *NT5E*-high groups (Figure 9F, Supplementary Figure S21A–G).

### 3.14. Predicting Drugs and Molecular Docking Analysis

We next sought to identify potential small-molecule therapeutics for HNSC by targeting ICD-related genes. Using upregulated ICD-related genes in HNSC as molecular signatures, we systematically screened the CMAP database and identified compounds that were negatively correlated with these signatures. Based on normalized connectivity scores (norm_cs), the top ten candidate compounds were fluvoxamine, BRD-K60306319, SB-206553, BRD-K21537090, L-692585, BRD-K12484704, BRD-K08039553, BRD-K49111930, BRD-K90987254, and BRD-K91880445 (Table 1).

**Figure 10:**
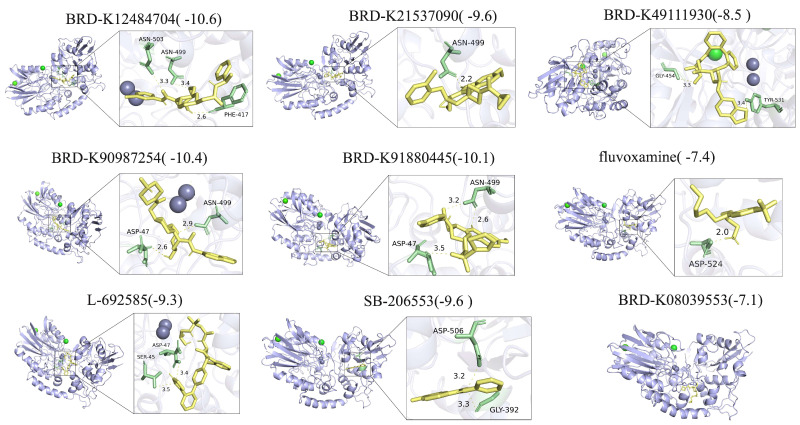
Molecular docking and between NT5E and potential drugs.

To further evaluate their therapeutic potential, molecular docking was performed between NT5E protein structures and the ten candidate compounds. The chemical structures of nine drugs (excluding BRD-K60306319) were retrieved from ZINC15, and complete NT5E protein structures were obtained from the Protein Data Bank. Docking analysis revealed strong binding affinities for all compounds, with docking scores below –5 (Figure 10). Detailed interaction analyses showed that fluvoxamine formed hydrogen bonds (H-bonds) with NT5E residues Asn503, Asn499, Phe417, and Asp524; BRD-K21537090 with Asn499; BRD-K49111930 with Gly454 and Tyr531; BRD-K90987254 and BRD-K91880445 with Asp47 and Asn499; L-692585 with Ser45 and Asp47; and SB-206553 with Gly392 and Asp506 (Figure 10). By contrast, BRD-K08039553 interacted mainly through non-bonded interactions (Figure 10).

### 3.15. Validation of the Expression Levels of ICD-Related Genes in HNSC

**Figure 11:**
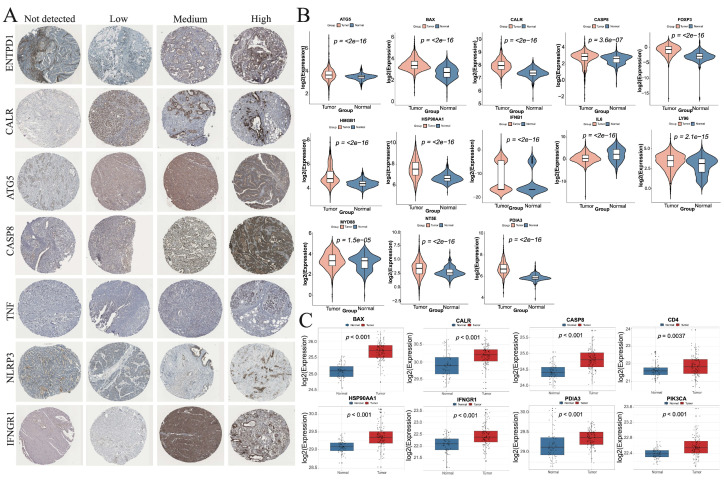
Validation of the expression levels of ICD-related genes in HNSC. (**A**) Protein expression levels of ENTPD1, CALR, ATG5, CASP8, TNF, NLRP3, and IFNGR1 in HNSC tumor tissues. (**B**) Differential RNA expression levels of ICD-related genes between normal and tumor tissues in HNSC patients from the CPTAC database. (**C**) Differential protein expression levels of ICD-related genes between normal and tumor tissues in HNSC patients from the CPTAC database.

From the HPA database, IHC results were available for seven ICD-related genes, including ENTPD1, CALR, ATG5, CASP8, TNF, NLRP3, and IFNGR1 in HNSC. The distribution and staining intensities of these proteins, ranging from not detected to low, medium, and high expression, are shown in Figure 11A. Furthermore, based on the RNA expression levels of ICD-related genes in HNSC patients from the CPTAC database (https://pdc.cancer.gov/pdc/cptac-pancancer, accessed on 1 December 2024), we found that the differential RNA expression of ICD-related genes was largely consistent with that observed in the TCGA database (Figure 11B). In addition, analysis of protein expression levels of ICD-related genes in HNSC patients from the CPTAC database revealed that the expression trends of BAX, CALR, CASP8, CD4, HSP90AA1, IFNGR1, PDIA3, and PIK3CA were consistent with their corresponding RNA expression profiles (Figure 11C).

## 4. Discussion

As a form of cell death that provokes immune activation, ICD possesses strong immunomodulatory properties and can stimulate multiple immune pathways during tumor cell demise [46]. Inducing ICD is considered a promising strategy to overcome key challenges in cancer immunotherapy, including low response rates and severe side effects [47]. ICD not only reverses the immunosuppressive tumor microenvironment but also enhances sensitivity to immunotherapy [41]. Therefore, identifying ICD-related biomarkers in pan-cancer cohorts holds potential to improve the efficacy of clinical cancer treatment. In this context, our study systematically investigated the roles of ICD-related genes across cancers and demonstrated their close associations with patient survival, the tumor microenvironment, and clinical characteristics.

Our analysis revealed distinct expression patterns of CASP1, FOXP3, and PDIA3 between normal and tumor tissues, highlighting their potential roles in shaping the tumor immune microenvironment. CASP1, a key inflammasome component that mediates IL-1β/IL-18 maturation and pyroptosis, was consistently elevated in normal tissues but downregulated in tumors [48,49]. This pattern suggests that tumors may suppress pyroptotic signaling and inflammatory cytokine release to evade immune surveillance [50]. Conversely, *FOXP3*, a master transcription factor of regulatory T cells (Tregs), is essential for immune tolerance and homeostasis [51]. In tumors, however, *FOXP3*^+^ Tregs suppress effector immune responses, facilitating immune evasion [52]. High intratumoral *FOXP3*^+^ Tregs are consistently associated with poor prognosis in various cancers due to their immunosuppressive activity. Moreover, *FOXP3* expression has been reported in tumor cells themselves, including breast, prostate, and melanoma, further contributing to immune evasion and adverse outcomes [52]. Therapeutically targeting *FOXP3* and Tregs is challenging, as disrupting their function risks compromising immune tolerance and inducing autoimmunity [53]. Importantly, the role of *FOXP3* varies across tumor types, highlighting the need for context-specific strategies. In addition, PDIA3, an endoplasmic reticulum chaperone involved in protein folding and MHC-I antigen presentation, was frequently upregulated in tumors [54]. While this may help cancer cells adapt to metabolic and proteotoxic stress, aberrant PDIA3 expression can impair antigen presentation, thereby weakening anti-tumor immune responses [55].

Collectively, these findings support a model in which tumors downregulate pro-inflammatory CASP1 while upregulating FOXP3 and PDIA3 to establish an immunosuppressive and immune-evasive niche. From a clinical perspective, these molecules may serve as complementary biomarkers: CASP1 as a marker of impaired inflammatory death, FOXP3 as an indicator of Treg-mediated suppression, and PDIA3 as a determinant of antigen presentation capacity. Therapeutically, strategies aiming to restore CASP1 activity, deplete or inhibit FOXP3+ Tregs, or target PDIA3-related antigen presentation defects could enhance immune surveillance and improve responsiveness to immunotherapy in multiple cancer types.

Using LASSO, SVM-RFE, and RF algorithms, LY96 was identified as a prognostic biomarker in four cancer types for OS and in three cancer types for DFI and PFS. LY96, also known as MD-2, is an essential co-receptor of Toll-like receptor 4 (TLR4), mediating pathogen recognition and activation of innate immune responses [56,57]. Mechanistically, LY96 facilitates the binding of lipopolysaccharide (LPS) and endogenous ligands to TLR4, triggering NF-κB signaling and the production of pro-inflammatory cytokines such as TNF-α, IL-6, and IL-1β [58,59]. While acute activation of this pathway may enhance anti-tumor immune surveillance, chronic activation contributes to tumor-promoting inflammation by fostering angiogenesis, immune evasion, and metastatic dissemination [60].

Moreover, elevated LY96 expression in the tumor microenvironment has been linked to the recruitment of immunosuppressive populations, including myeloid-derived suppressor cells (MDSCs) and M2 macrophages, thereby suppressing cytotoxic T-cell function and exacerbating tumor progression [61,62,63,64]. This dual role of LY96—acting both as a driver of immune activation and as a mediator of tumor-supportive inflammation—likely underlies its association with adverse prognosis in multiple cancers.

From a clinical standpoint, LY96 may serve as a predictive biomarker for patient stratification, particularly in identifying individuals more likely to respond to therapies targeting the TLR4/MD-2 axis [65,66,67]. Pharmacological disruption of LY96–TLR4 interactions or inhibition of downstream NF-κB signaling has shown promise in preclinical studies [68,69,70], and could represent a therapeutic avenue to attenuate tumor-promoting inflammation while restoring anti-tumor immunity.

NT5E has previously been reported as a biomarker in HNSC [61], and our study further confirmed this observation through survival analysis and multiple machine learning approaches, thereby reinforcing its clinical significance. Mechanistically, NT5E encodes CD73, a rate-limiting ectoenzyme in the purinergic signaling pathway that catalyzes the conversion of extracellular AMP into adenosine [71,72]. The accumulation of adenosine in the tumor microenvironment (TME) promotes immune evasion by suppressing cytotoxic T-cell and NK-cell activity, while enhancing the function of regulatory T cells (Tregs) [73,74]. In addition, NT5E-driven adenosine signaling has been implicated in epithelial–mesenchymal transition (EMT), angiogenesis, metastatic dissemination, and therapeutic resistance by activating pro-survival signaling and facilitating tissue repair mechanisms [75].

Recent experimental studies further corroborate the oncogenic role of NT5E/CD73 in HNSC. Shi et al. demonstrated that CD73 acts as an effector of EGFR/EMT-mediated invasion, and its inhibition with the antibody 22E6 synergized with Cetuximab to markedly reduce invasiveness [76]. Similarly, Xue et al. showed that CD73 overexpression correlates with poor prognosis and promotes invadopodia formation, while its inhibition diminished invasive potential [77]. Beyond cell-intrinsic mechanisms, Lu et al. reported that CD73 is highly enriched in small extracellular vesicles (sEVs) derived from HNSCC, highlighting its role in extracellular immune modulation and supporting its utility as a circulating biomarker [78]. Moreover, Shen et al. integrated multi-omics and single-cell analyses and found that high CD73 expression was associated with CD8+ T-cell depletion, enhanced EMT and metastasis, and poor response to PD-1/PD-L1 blockade, underscoring its dual contribution to immune evasion and tumor aggressiveness [79].

Clinically, NT5E holds considerable promise as both a prognostic biomarker and a therapeutic target. Its elevated expression is strongly associated with poor overall and disease-specific survival, suggesting utility in risk stratification assays for HNSC [80,81]. Additionally, NT5E may serve as a companion biomarker for immunotherapy, as adenosine-mediated immunosuppression represents a major barrier to effective immune checkpoint blockade [71,82]. Targeted therapies against CD73 or the adenosine signaling axis are currently under active clinical development, with several trials evaluating CD73 inhibitors or anti-adenosine receptor agents in combination with PD-1/PD-L1 inhibitors [83,84]. Together, our findings not only validate NT5E as a robust computationally derived biomarker but also highlight its translational potential as a druggable target in HNSC.

Based on drug prediction and molecular docking analyses, ten candidate compounds were identified for HNSC treatment from CMAP using upregulated ICD-related gene signatures. Among them, several agents demonstrated notable therapeutic potential with distinct mechanisms of action. Fluvoxamine, a selective serotonin reuptake inhibitor (SSRI), can induce endoplasmic reticulum stress to trigger cancer cell apoptosis and inhibit protective autophagy, thereby sensitizing cancer cells to additional stressors or therapies [85]; it may also indirectly modulate pro-survival signaling pathways such as PI3K/AKT/mTOR and MAPK [86]. SB-206553, an antagonist of 5-hydroxytryptamine receptor subtypes 2B and 2C (5-HT2B/2C), may suppress cancer-promoting processes—including proliferation, invasion, angiogenesis, and metastasis—by blocking serotonin-driven oncogenic signaling [87]. It may further reshape the tumor microenvironment through modulation of 5-HT receptors on fibroblasts and immune cells [88]. L-692585, a growth hormone secretagogue receptor (GHSR) agonist, can inhibit Ghrelin/GHSR signaling, a pathway implicated in multiple cancers for promoting mitogenesis, inhibiting apoptosis, and enhancing angiogenesis and metastasis [89,90,91,92].

In contrast, BRD-K60306319, BRD-K21537090, BRD-K12484704, BRD-K08039553, BRD-K49111930, BRD-K90987254, and BRD-K91880445 represent promising but largely unexplored candidates in oncology. Further experimental and clinical studies are needed to assess their therapeutic efficacy and applicability in malignancies, including pancreatic cancer. Notably, our molecular docking analysis demonstrated strong interactions between these BRD compounds and specific amino acid residues of the NT5E protein. Future investigations should clarify whether these agents exert anticancer effects in HNSC through NT5E inhibition.

However, this study has several limitations. First, the heterogeneity of data from the GTEx and TCGA databases may have affected the accuracy of our analyses. Second, comprehensively verifying the associations between 34 ICD-related genes and 33 cancer types represents a substantial undertaking that cannot be achieved in a short timeframe. Moreover, no single gene demonstrated consistent associations or functions across all cancers, as the significance of individual genes varied depending on cancer type and analytic dimension. Therefore, further studies are required to clarify the specificity and applicability of these genes in distinct tumor contexts.

## 5. Conclusions

We analyzed the expression of 34 ICD-related regulatory genes across 33 cancer types. These results enhance our understanding of the regulatory mechanisms of ICD in pan-cancer and reveal novel biomarkers in certain cancers for predicting immunotherapy response and improving patient outcomes. Collectively, our work provides a systematic framework to elucidate the associations between ICD-related genes and multiple cancers, highlighting their roles in tumor progression and potential as therapeutic targets. We further constructed ICD-associated molecular subtypes and developed prognostic models that integrated clinicopathological features of cancer patients. Our findings demonstrate that ICD levels are closely linked to tumor progression, thereby deepening our understanding of ICD in pan-cancer and offering valuable insights for the identification of novel prognostic and immunotherapy response biomarkers. Moreover, we identified *NT5E* as a potential biomarker in HNSC, where it mediates TME remodeling and cell–cell communication. Finally, by leveraging the CMAP database, we identified candidate compounds that may target ICD-related genes and could represent promising therapeutic options for HNSC.

## Figures and Tables

**Table 1:** Drug repositioning results were obtained based on the up-regulated ICD-related genes of HNSC patients in the CMAP platform.

Rank	pert_id	CMAP Name	Mechanism of Action	Raw_cs	Norm_cs
1	BRD-K72676686	fluvoxamine		−0.7568	−2.1254
2	BRD-K60306319	BRD-K60306319		−0.7389	−2.0752
3	BRD-K36395411	SB-206553	Serotonin receptor antagonist	−0.7342	−2.0621
4	BRD-K21537090	BRD-K21537090		−0.7323	−2.0565
5	BRD-K70241288	L-692585	Growth hormone releasing peptide ligand agonist	−0.7304	−2.0513
6	BRD-K12484704	BRD-K12484704		−0.7293	−2.0483
7	BRD-K08039553	BRD-K08039553		−0.7247	−2.0352
8	BRD-K49111930	BRD-K49111930		−0.7231	−2.0307
9	BRD-K90987254	BRD-K90987254		−0.7182	−2.0172
10	BRD-K91880445	BRD-K91880445		−0.7167	−2.0127

## Data Availability

Data of mRNA expression profiles, mutation annotation data, CNV data, and clinical metadata about 33 cancer types were collected from TCGA database in UCSC (https://xenabrowser.net/).

## Supplementary Materials

The following supporting information can be downloaded at https://www.mdpi.com/article/10.3390/cimb47100812/s1.

## Abbreviations

ACC: Adrenocortical carcinoma; BRCA: Breast invasive carcinoma; BLCA: Bladder urothelial cancer; CESC: Cervical squamous cell carcinoma and endocervical adenocarcinoma; COAD: Colon adenocarcinoma; CHOL: Cholangiocarcinoma; DLBC: Diffuse Large B cell; ESCA: Esophageal carcinoma; GBM: Glioblastoma multiforme; HNSC: Head and neck squamous cell carcinoma; KIRP: Kidney renal papillary cell carcinoma; KIRC: Kidney renal cell carcinoma; KICH: Kidney chromophobe; LGG: Brain low grade glioma; LUAD: Lung adenocarcinoma; LUSC: Lung squamous cell carcinoma; LIHC: Liver hepatocellular carcinoma; LAML: Acute myeloid leukemia; MESO: Mesothelioma; OV: Ovarian cystadenocarcinoma; PRAD: Prostate adenocarcinoma; PAAD: Pancreatic adenocarcinoma; PCPG: Phenochromocytoma and paraganglioma; READ: Rectum adenocarcinoma; SARC: Sarcoma; STAD: Stomach adenocarcinoma; SKCM: Skin cutaneous melanoma; THCA: Thyroid carcinoma; TGCT: Testicular germ cell tumors; THYM: Thymoma; UCEC: Uterine corpus endometrial carcinoma UCS Uterine carcinosarcoma; UVM: Uveal Melanoma; UCS: Uterine Carcinosarcoma.
