# Supplementary material for: Constructing a Pan-Cancer Prognostic Model via Machine Learning Based on Immunogenic Cell Death Genes and Identifying NT5E as a Biomarker in Head and Neck Cancer
Source: Curr Issues Mol Biol. 2025 Oct 1;47(10):812. doi: 10.3390/cimb47100812 (PMC12564767; doi:10.3390/cimb47100812)
Supplement: Supplementary file 1 [file cimb-47-00812-s001.zip › cimb-3868671-supplementary/Supplementary_0930/Supplementary Figure s20.pdf]

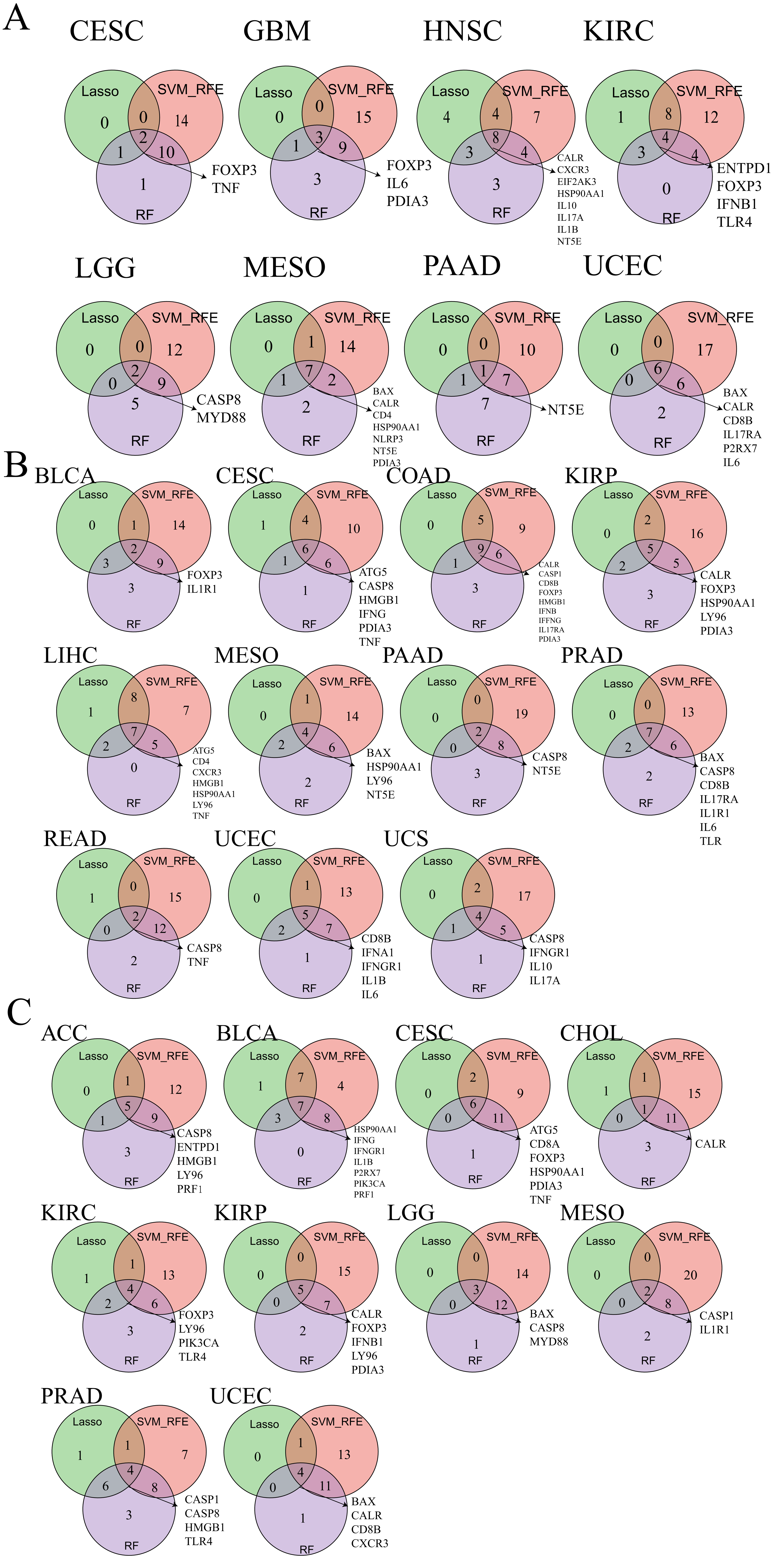
A
CESC
Lasso 0
SVM_RFE 14
0 2 10 1 1
RF
FOXP3
TNF
GBM
Lasso 0
SVM_RFE 15
0 3 9 1 3
RF
FOXP3
IL6
PDIA3
HNSC
Lasso 4
SVM_RFE 7
4 8 4 3 3
RF
CALR
CXCR3
EIF2AK3
HSP90AA1
IL10
IL17A
IL1B
NT5E
KIRC
Lasso 1
SVM_RFE 12
8 4 4 3 0
RF
ENTPD1
FOXP3
IFNB1
TLR4
LGG
Lasso 0
SVM_RFE 12
0 2 9 0 5
RF
CASP8
MYD88
MESO
Lasso 0
SVM_RFE 14
1 7 2 1 2
RF
BAX
CALR
CD4
HSP90AA1
NLRP3
NT5E
PDIA3
PAAD
Lasso 0
SVM_RFE 10
0 1 7 1 7
RF
NT5E
UCEC
Lasso 0
SVM_RFE 17
0 6 6 0 2
RF
BAX
CALR
CD8B
IL17RA
P2RX7
IL6
B
BLCA
Lasso 0
SVM_RFE 14
1 2 9 3 3
RF
FOXP3
IL1R1
CESC
Lasso 1
SVM_RFE 10
4 6 6 1 1
RF
ATG5
CASP8
HMGB1
IFNG
PDIA3
TNF
COAD
Lasso 0
SVM_RFE 9
5 9 6 1 3
RF
CALR
CASP1
CD8B
FOXP3
HMGB1
IFNB
IFFNG
IL17RA
PDIA3
KIRP
Lasso 0
SVM_RFE 16
2 5 5 2 3
RF
CALR
FOXP3
HSP90AA1
LY96
PDIA3
LIHC
Lasso 1
SVM_RFE 7
8 7 5 2 0
RF
ATG5
CD4
CXCR3
HMGB1
HSP90AA1
LY96
TNF
MESO
Lasso 0
SVM_RFE 14
1 4 6 2 2
RF
BAX
HSP90AA1
LY96
NT5E
PAAD
Lasso 0
SVM_RFE 19
0 2 8 0 3
RF
CASP8
NT5E
PRAD
Lasso 0
SVM_RFE 13
0 7 6 2 2
RF
BAX
CASP8
CD8B
IL17RA
IL1R1
IL6
TLR
READ
Lasso 1
SVM_RFE 15
0 2 12 0 2
RF
CASP8
TNF
UCEC
Lasso 0
SVM_RFE 13
1 5 7 2 1
RF
CD8B
IFNA1
IFNGR1
IL1B
IL6
UCS
Lasso 0
SVM_RFE 17
2 4 5 1 1
RF
CASP8
IFNGR1
IL10
IL17A
C
ACC
Lasso 0
SVM_RFE 12
1 5 9 1 3
RF
CASP8
ENTPD1
HMGB1
LY96
PRF1
BLCA
Lasso 1
SVM_RFE 4
7 7 8 3 0
RF
HSP90AA1
IFNG
IFNGR1
IL1B
P2RX7
PIK3CA
PRF1
CESC
Lasso 0
SVM_RFE 9
2 6 11 0 1
RF
ATG5
CD8A
FOXP3
HSP90AA1
PDIA3
TNF
CHOL
Lasso 1
SVM_RFE 15
1 1 11 0 3
RF
CALR
KIRC
Lasso 1
SVM_RFE 13
1 4 6 2 3
RF
FOXP3
LY96
PIK3CA
TLR4
KIRP
Lasso 0
SVM_RFE 15
0 5 7 0 2
RF
CALR
FOXP3
IFNB1
LY96
PDIA3
LGG
Lasso 0
SVM_RFE 14
0 3 12 0 1
RF
BAX
CASP8
MYD88
MESO
Lasso 0
SVM_RFE 20
0 2 8 0 2
RF
CASP1
IL1R1
PRAD
Lasso 1
SVM_RFE 7
1 4 8 6 3
RF
CASP1
CASP8
HMGB1
TLR4
UCEC
Lasso 0
SVM_RFE 13
1 4 11 0 1
RF
BAX
CALR
CD8B
CXCR3
