# Supplementary figures and images for: Constructing a Pan-Cancer Prognostic Model via Machine Learning Based on Immunogenic Cell Death Genes and Identifying NT5E as a Biomarker in Head and Neck Cancer
Source: Curr Issues Mol Biol. 2025 Oct 1;47(10):812. doi: 10.3390/cimb47100812 (PMC12564767; doi:10.3390/cimb47100812)

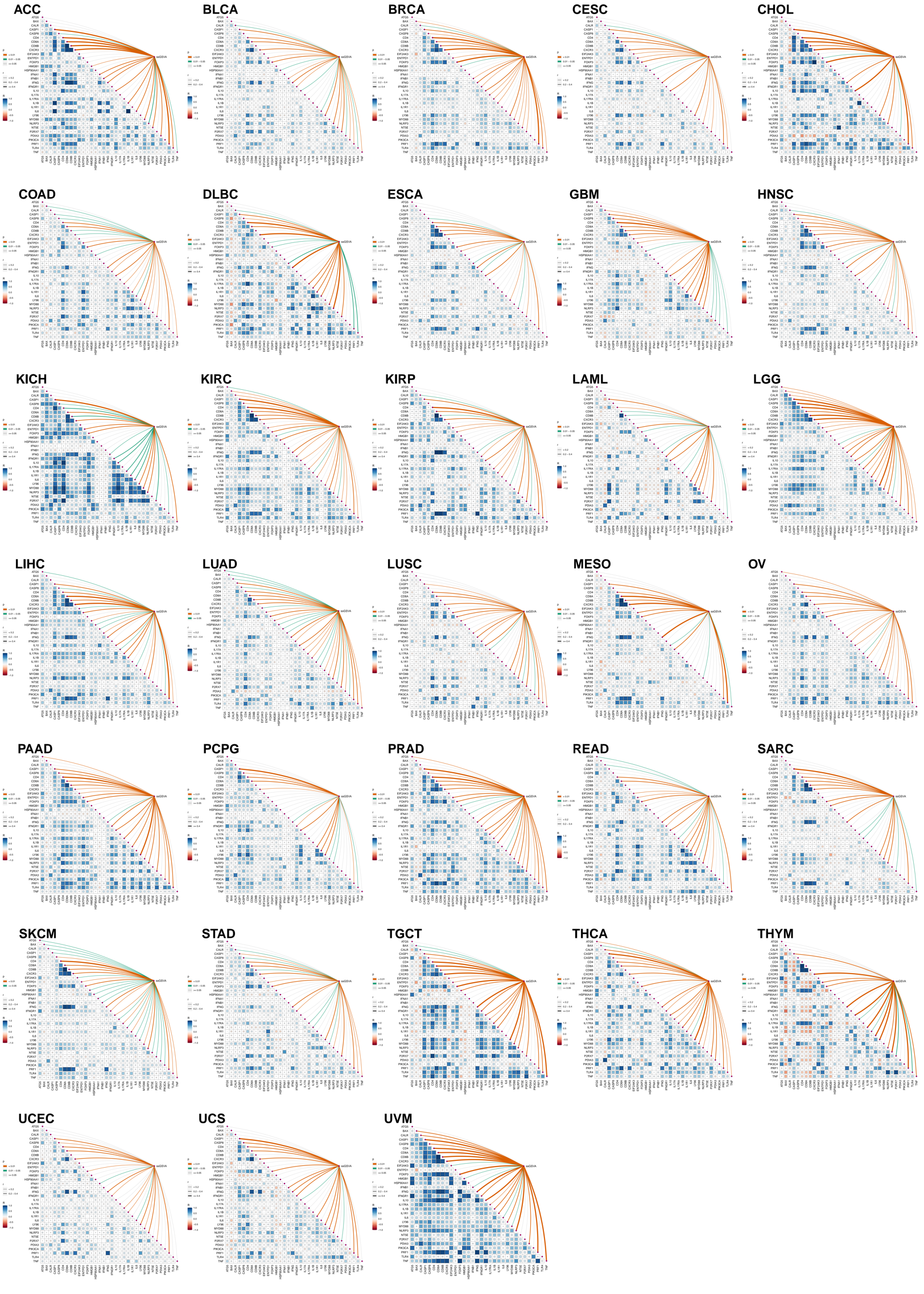

Supplement: Supplementary file 1 [file cimb-47-00812-s001.zip › cimb-3868671-supplementary/Supplementary_0930/Supplementary Figure s1.pdf]

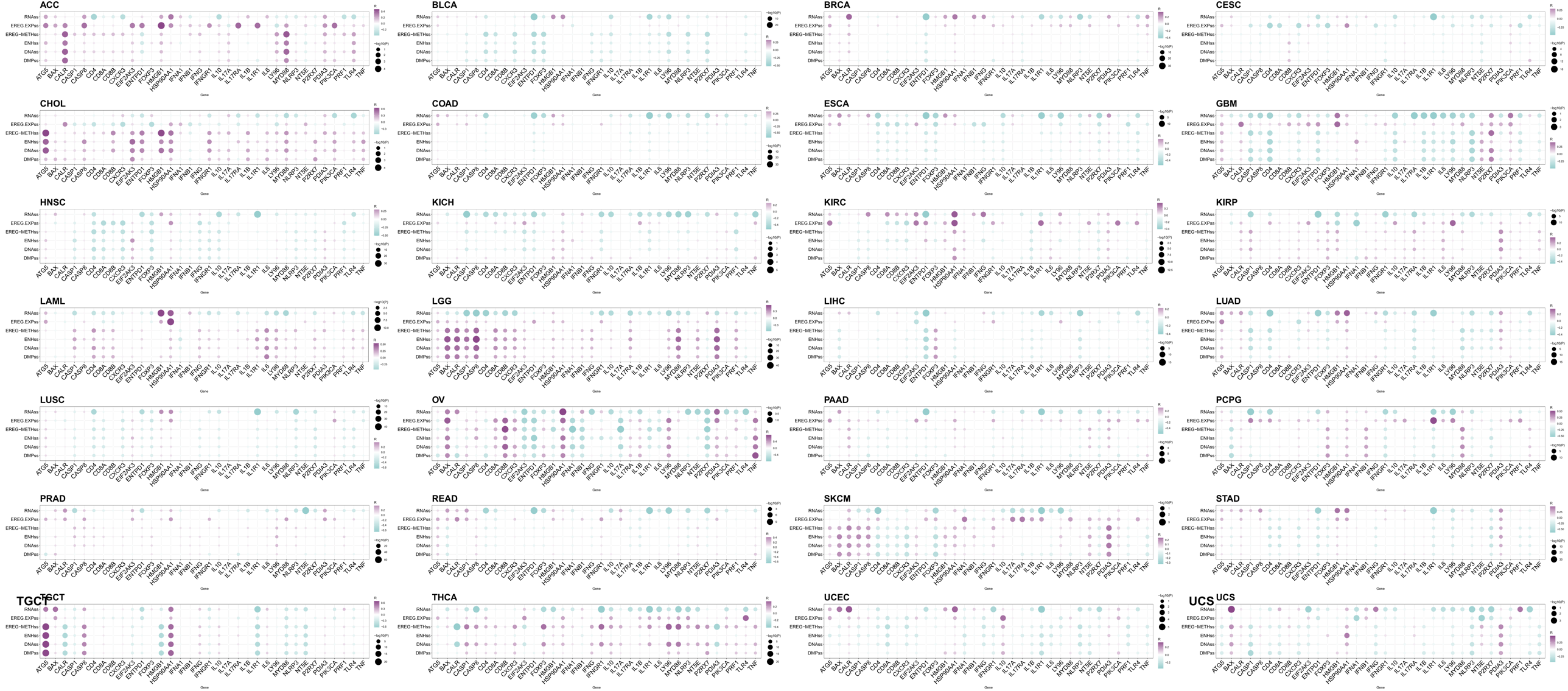

Supplement: Supplementary file 1 [file cimb-47-00812-s001.zip › cimb-3868671-supplementary/Supplementary_0930/Supplementary Figure s10.pdf]

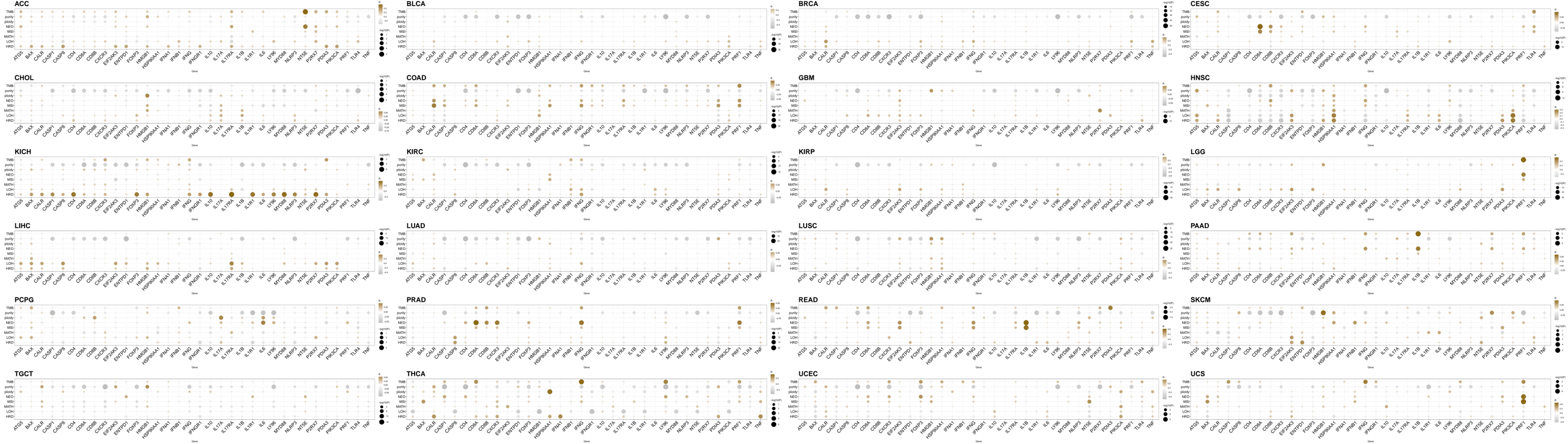

Supplement: Supplementary file 1 [file cimb-47-00812-s001.zip › cimb-3868671-supplementary/Supplementary_0930/Supplementary Figure s11.pdf]

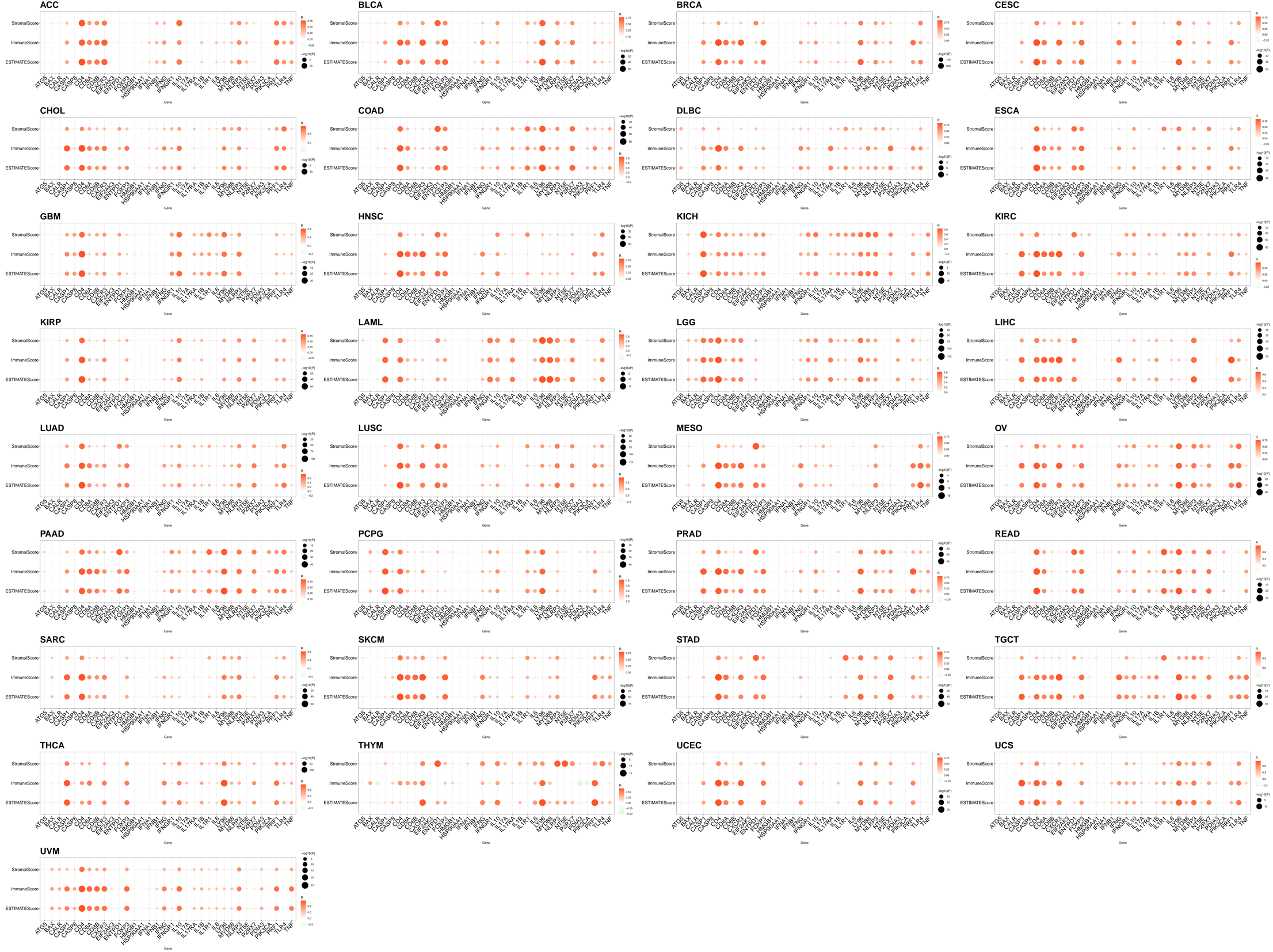

Supplement: Supplementary file 1 [file cimb-47-00812-s001.zip › cimb-3868671-supplementary/Supplementary_0930/Supplementary Figure s12.pdf]

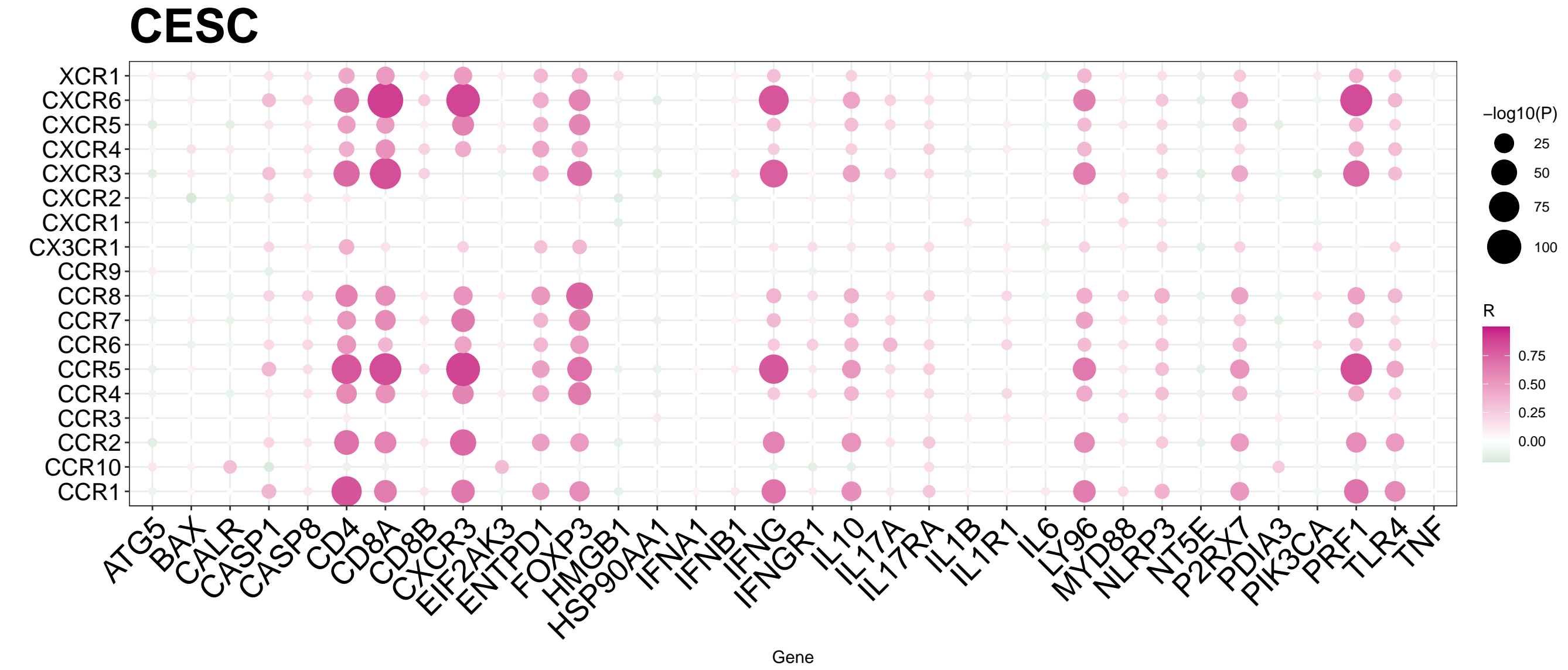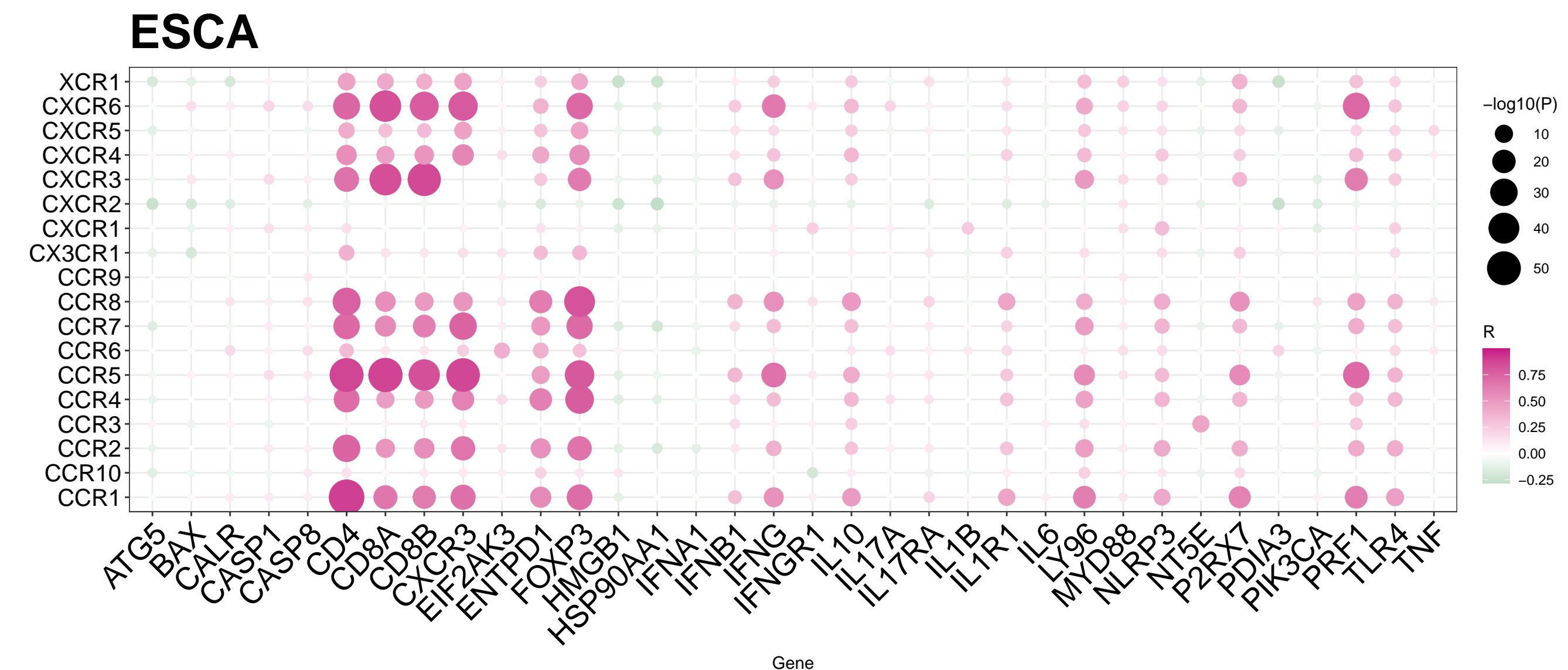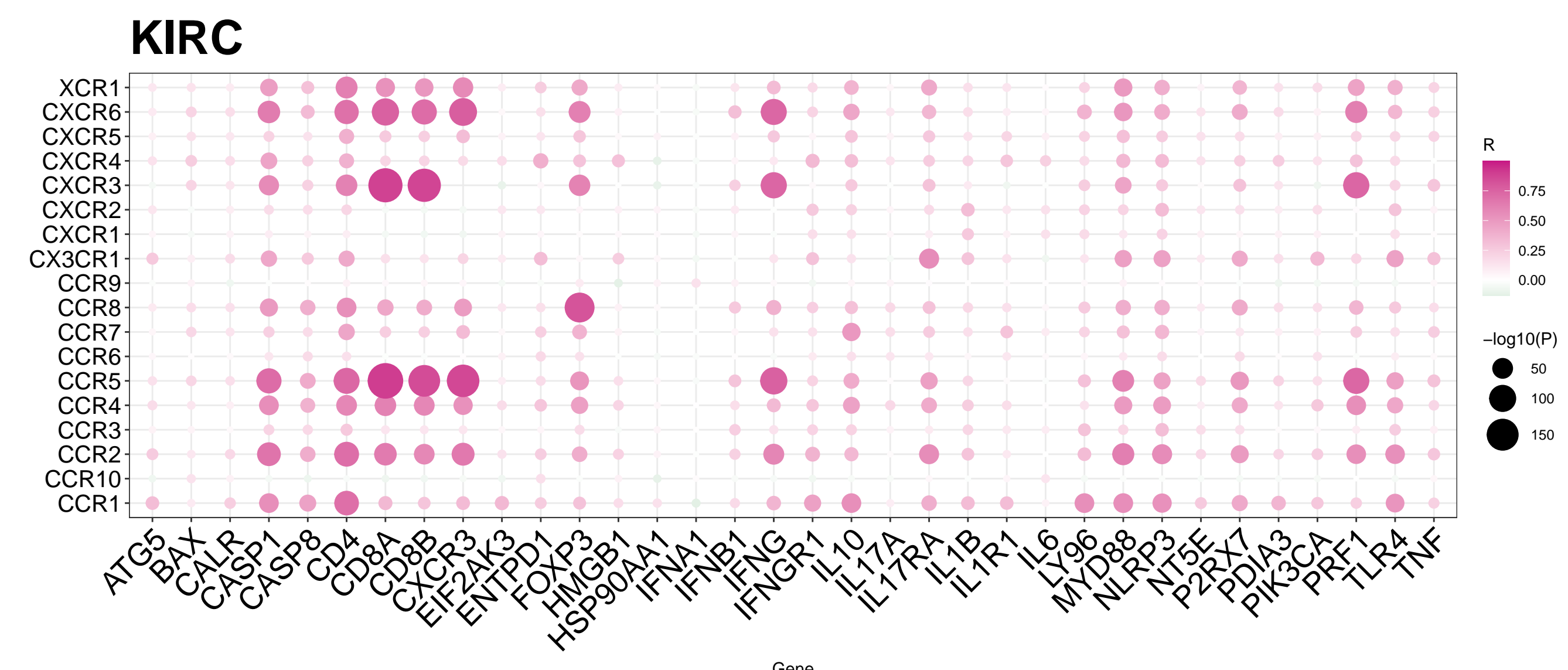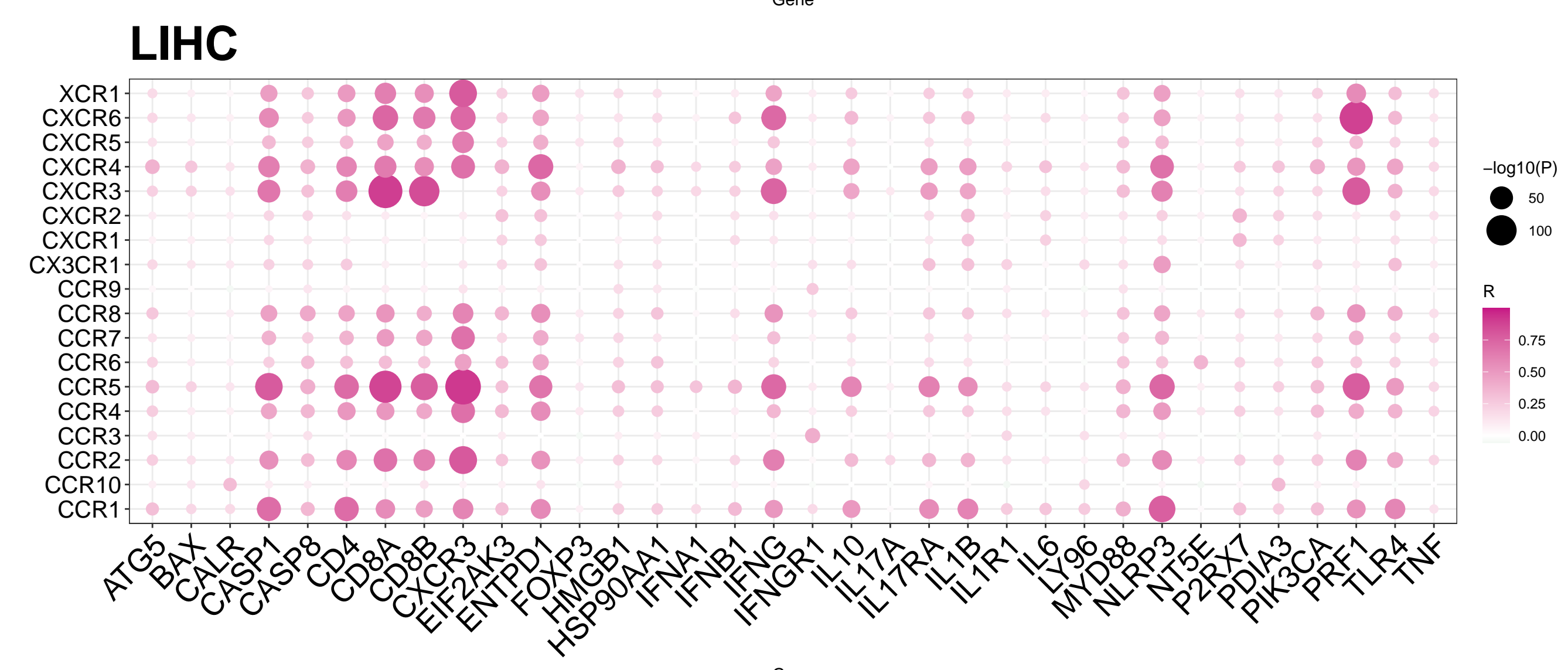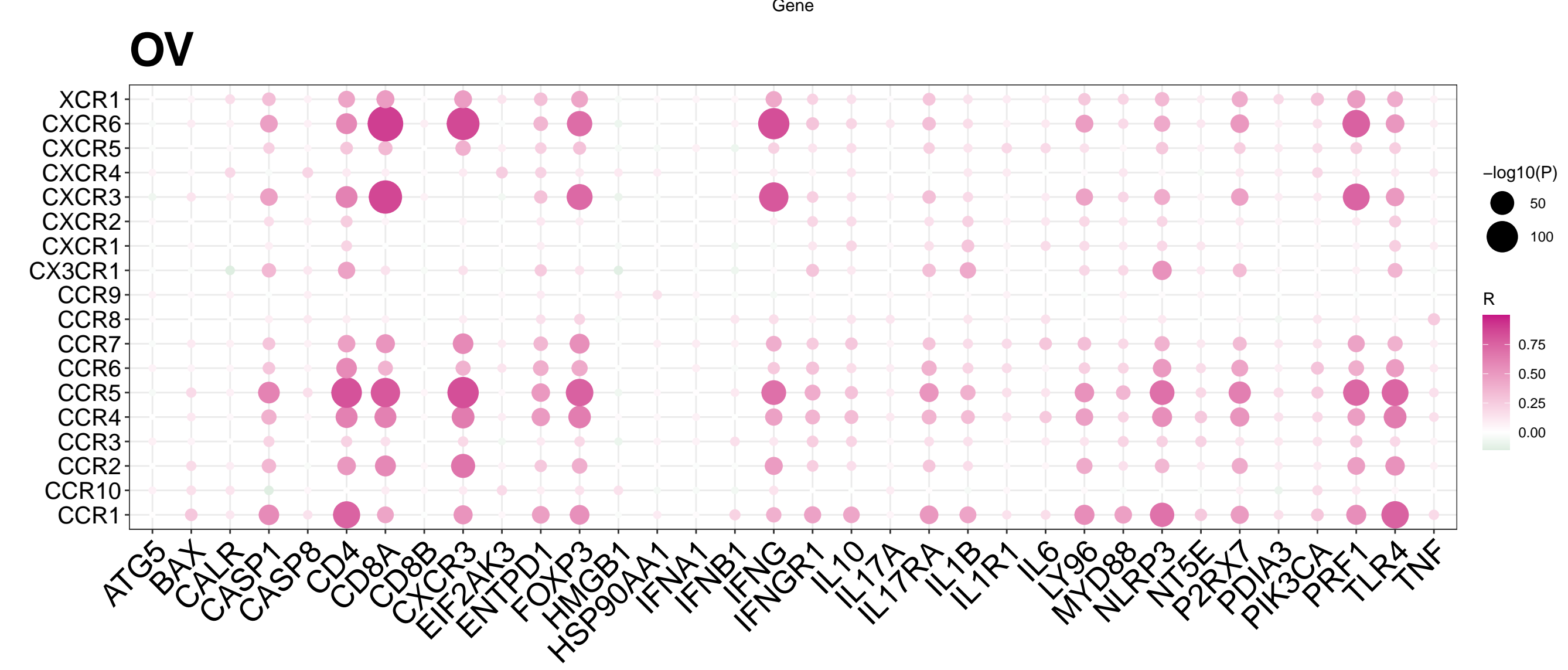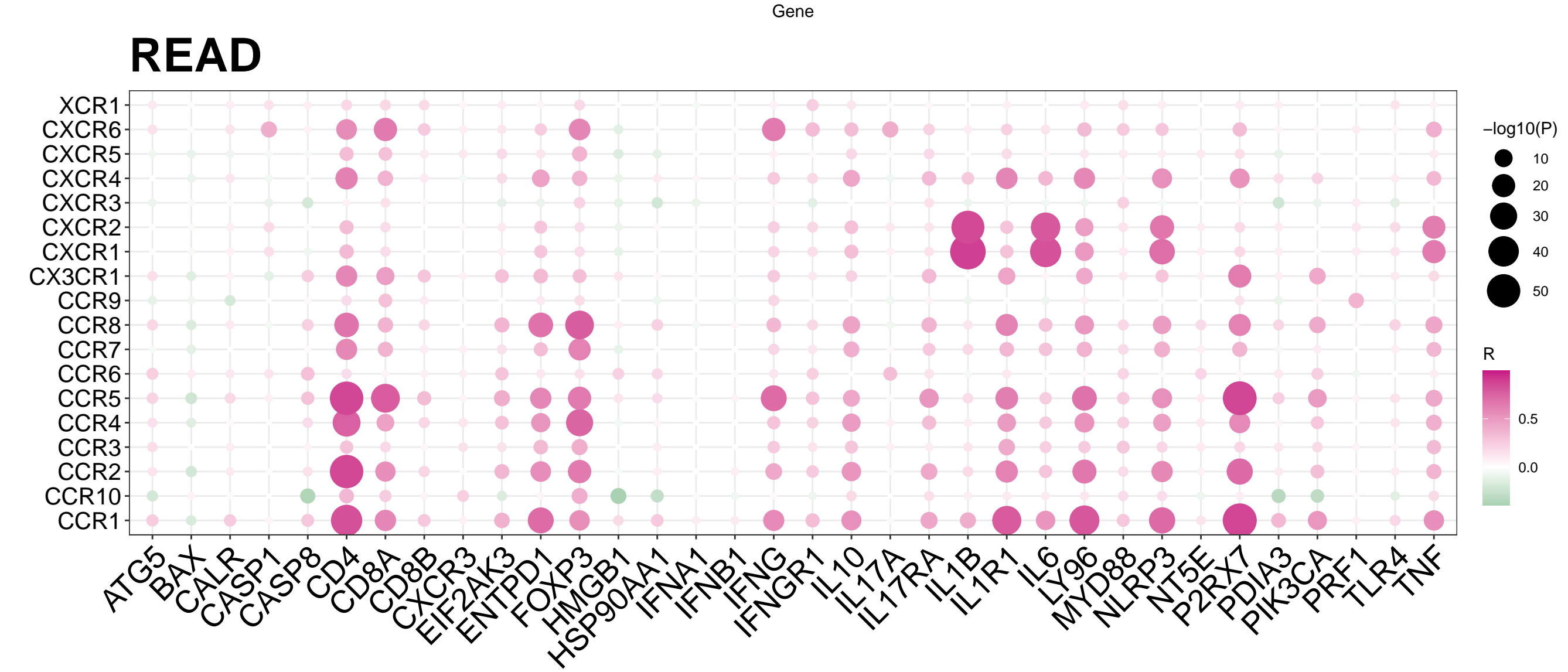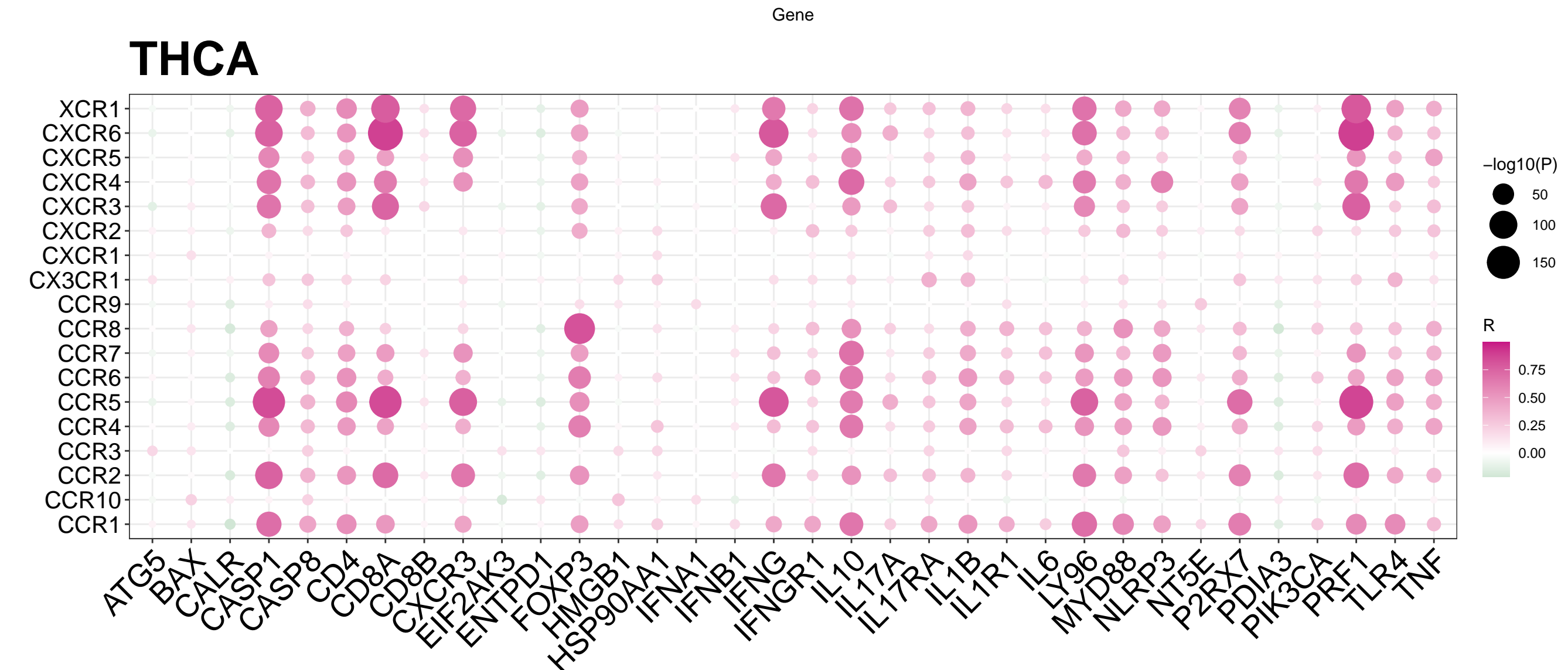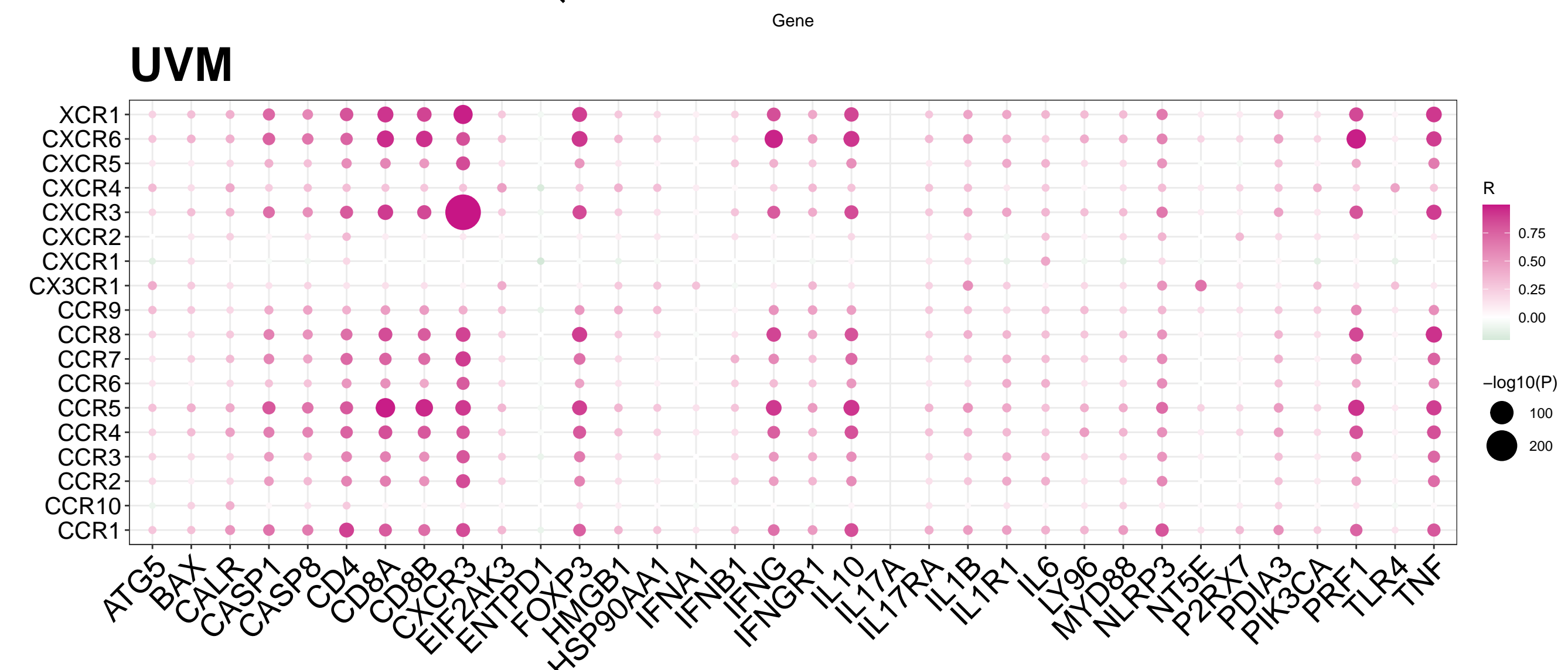

Supplement: Supplementary file 1 [file cimb-47-00812-s001.zip › cimb-3868671-supplementary/Supplementary_0930/Supplementary Figure s13-Receptor.pdf]

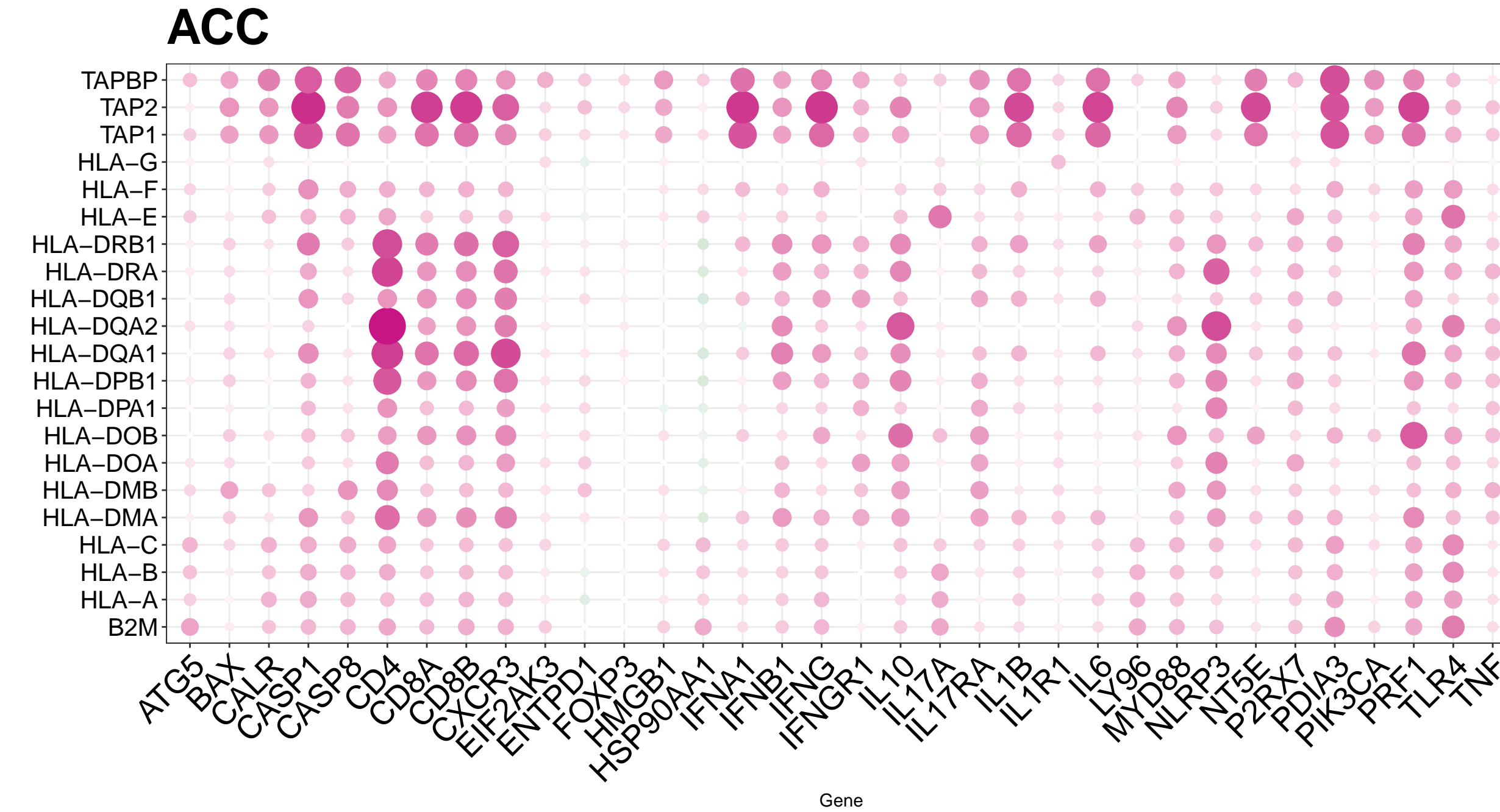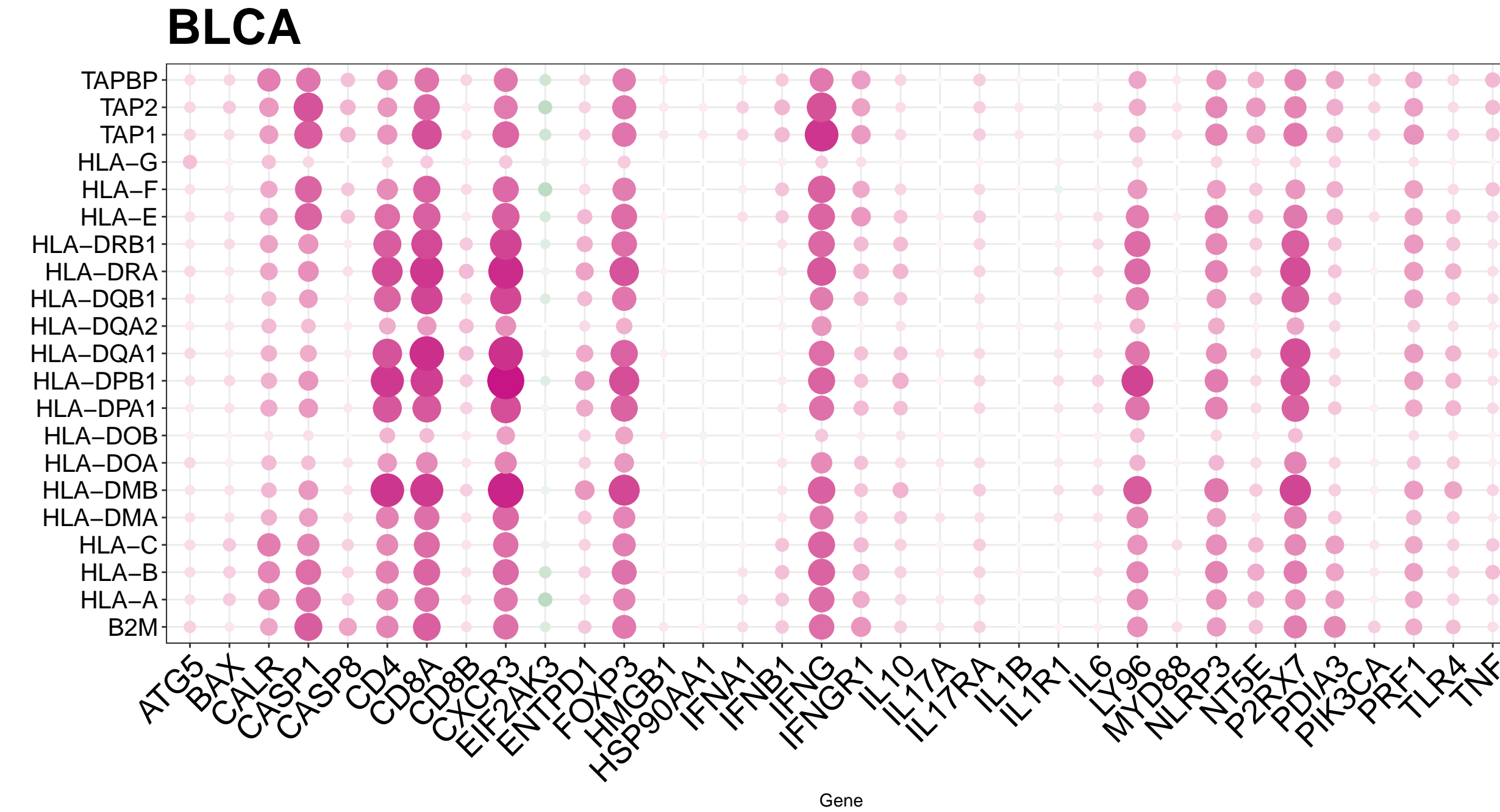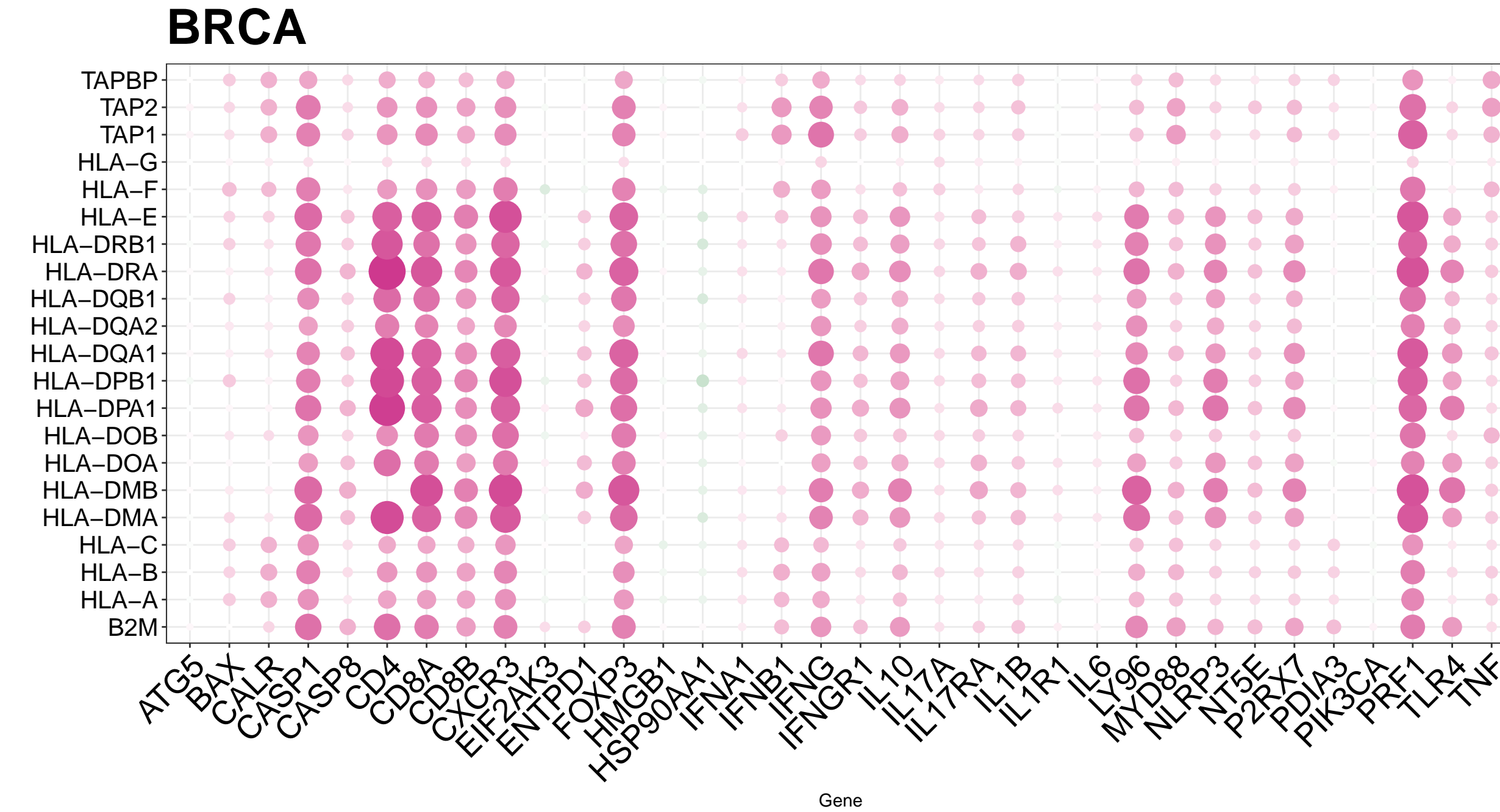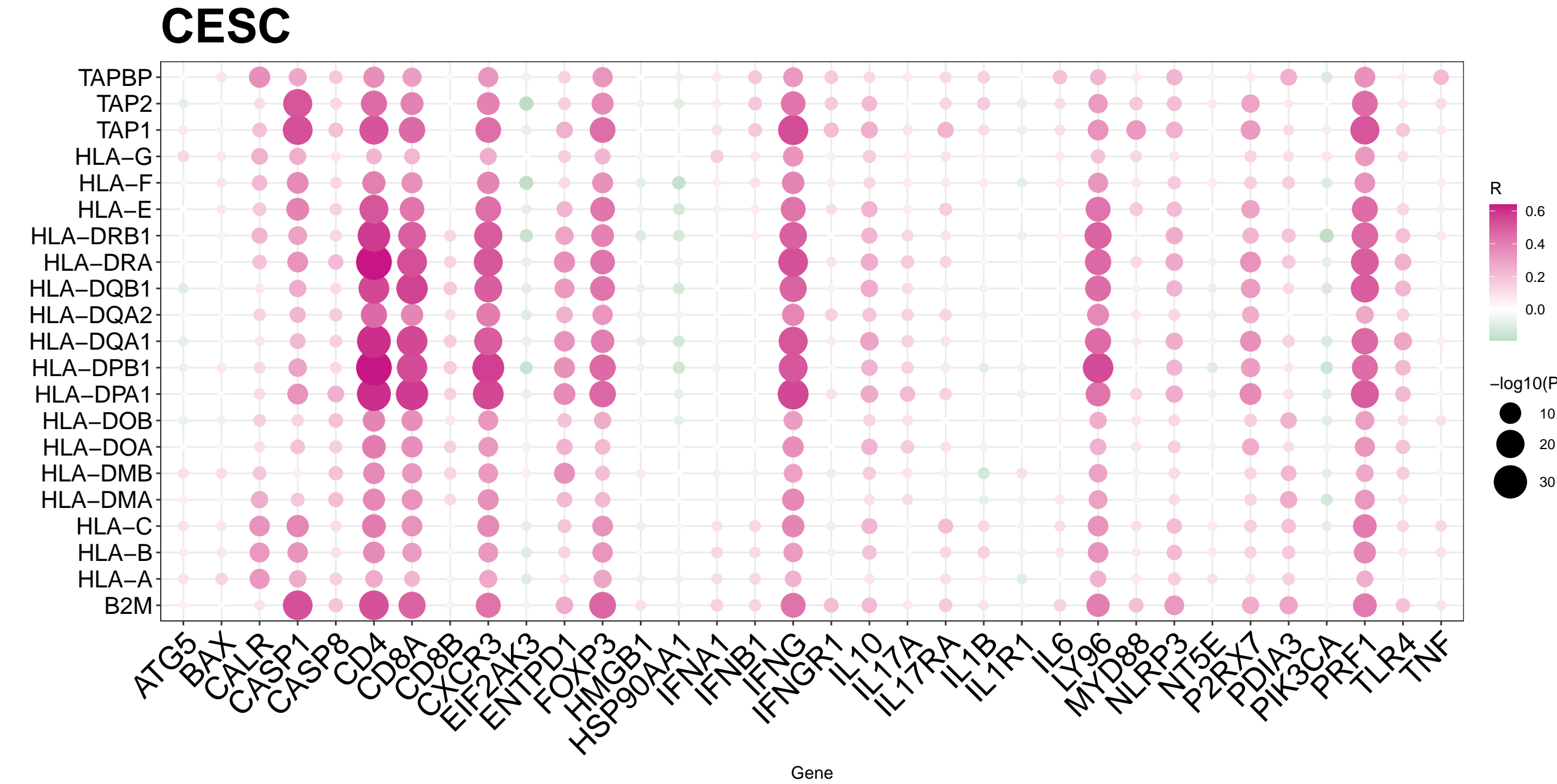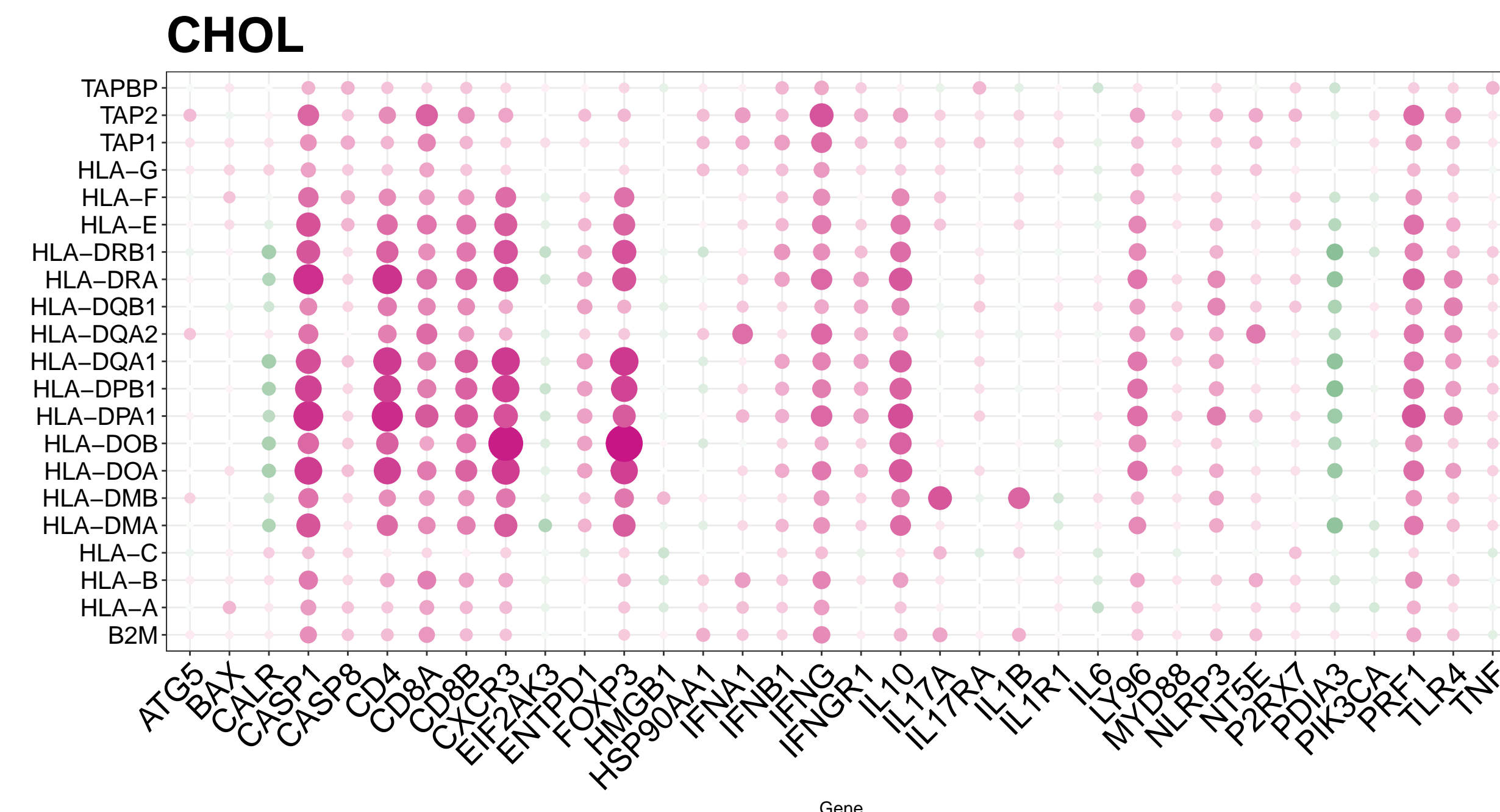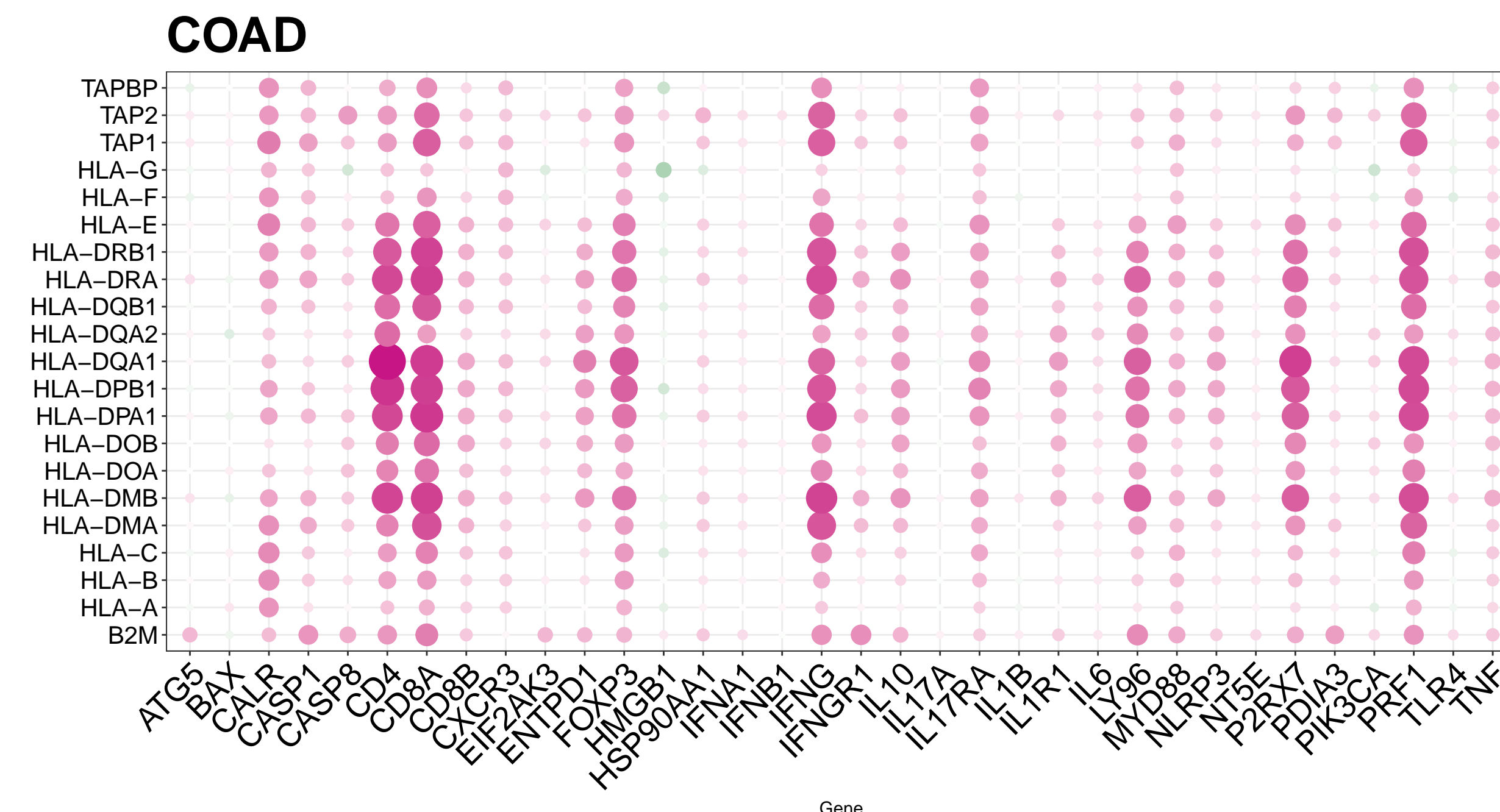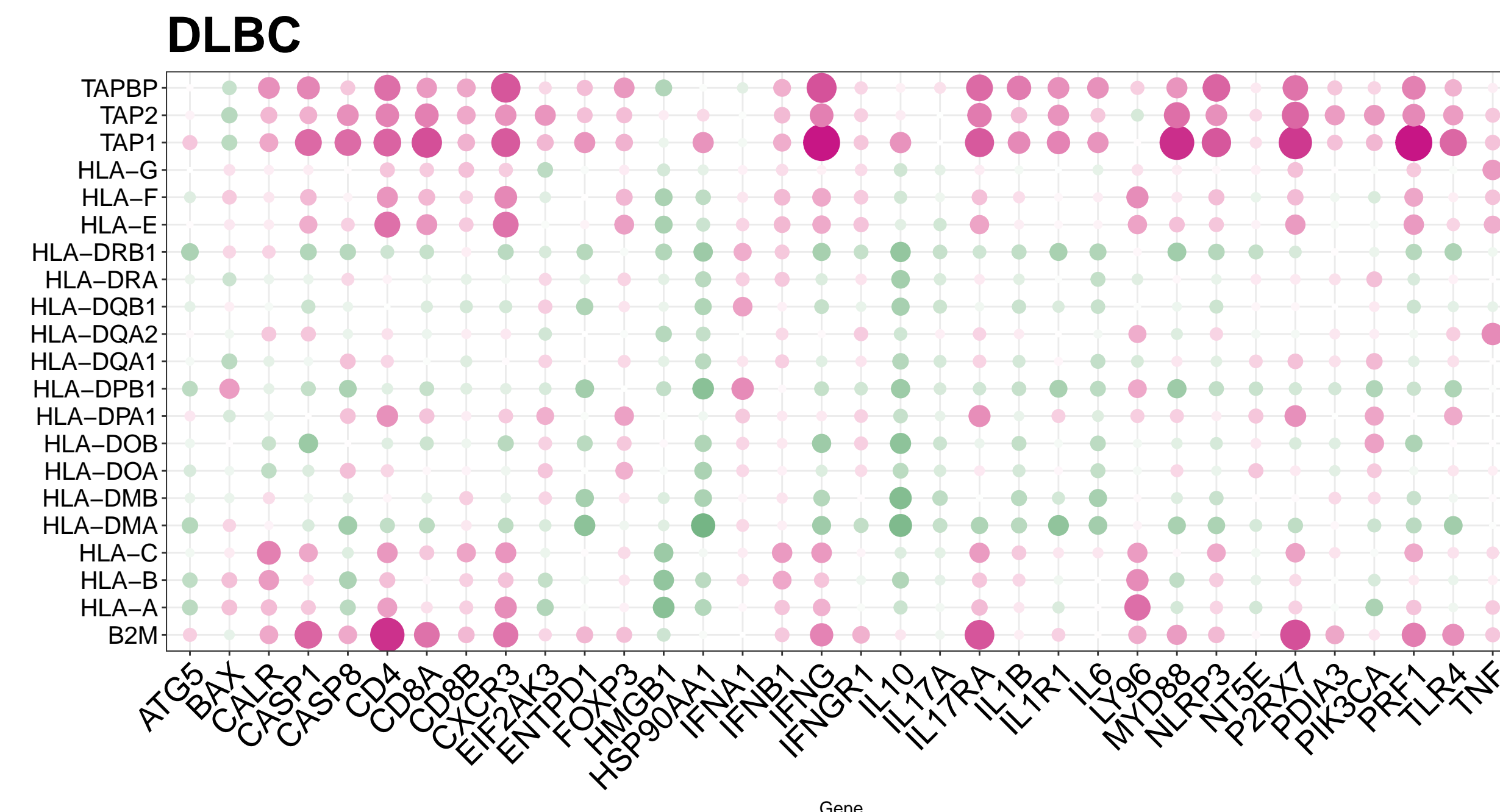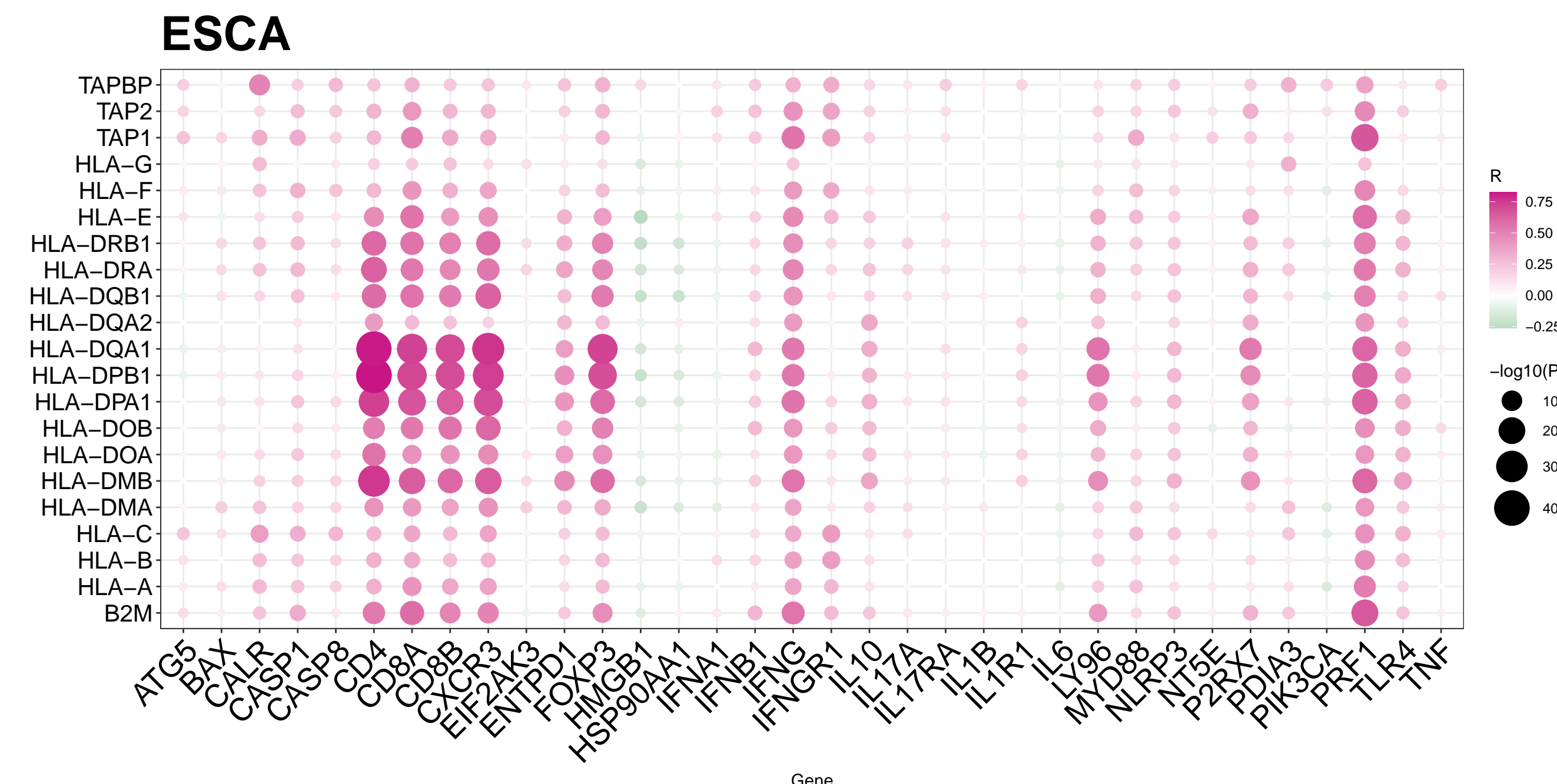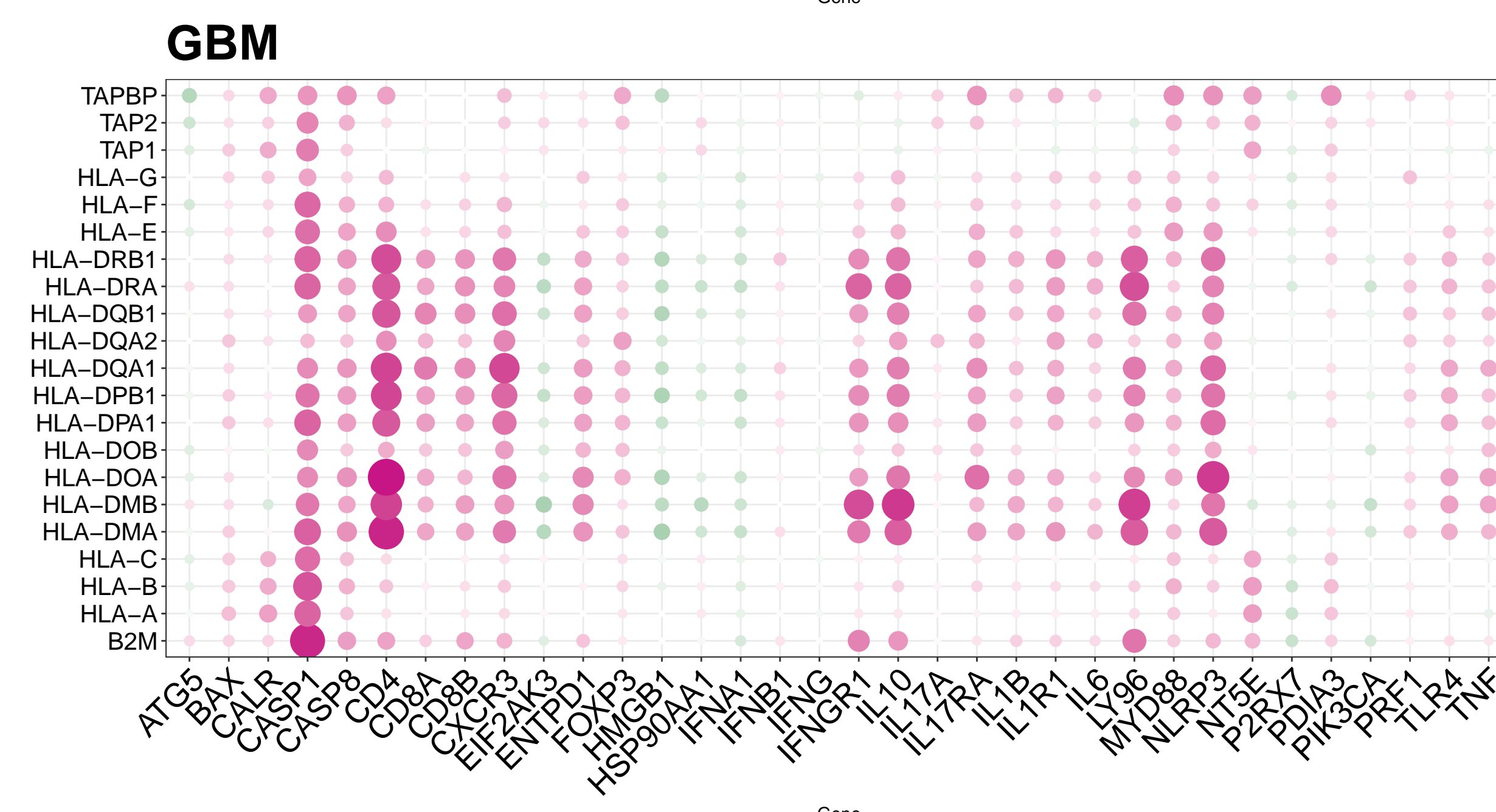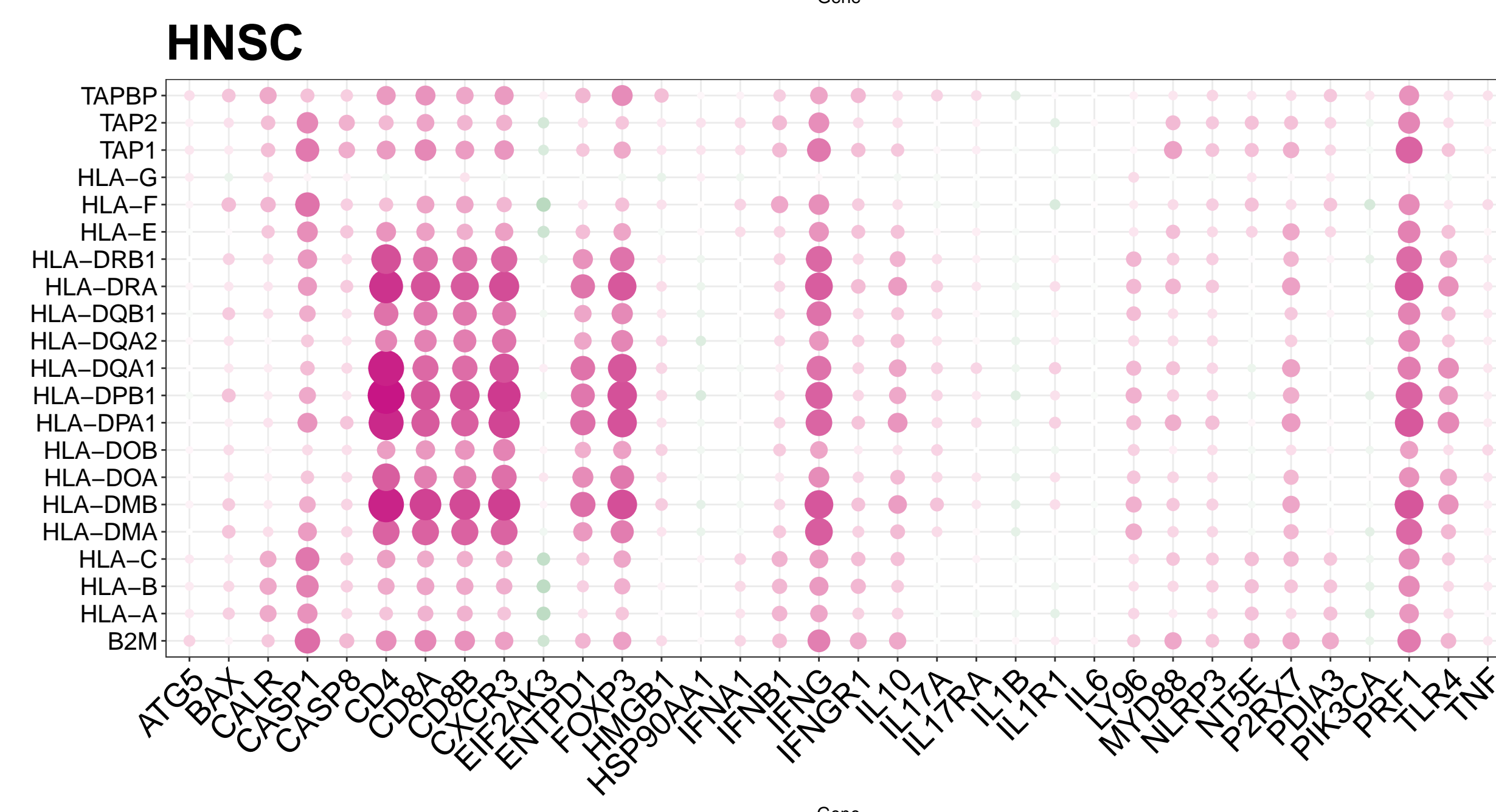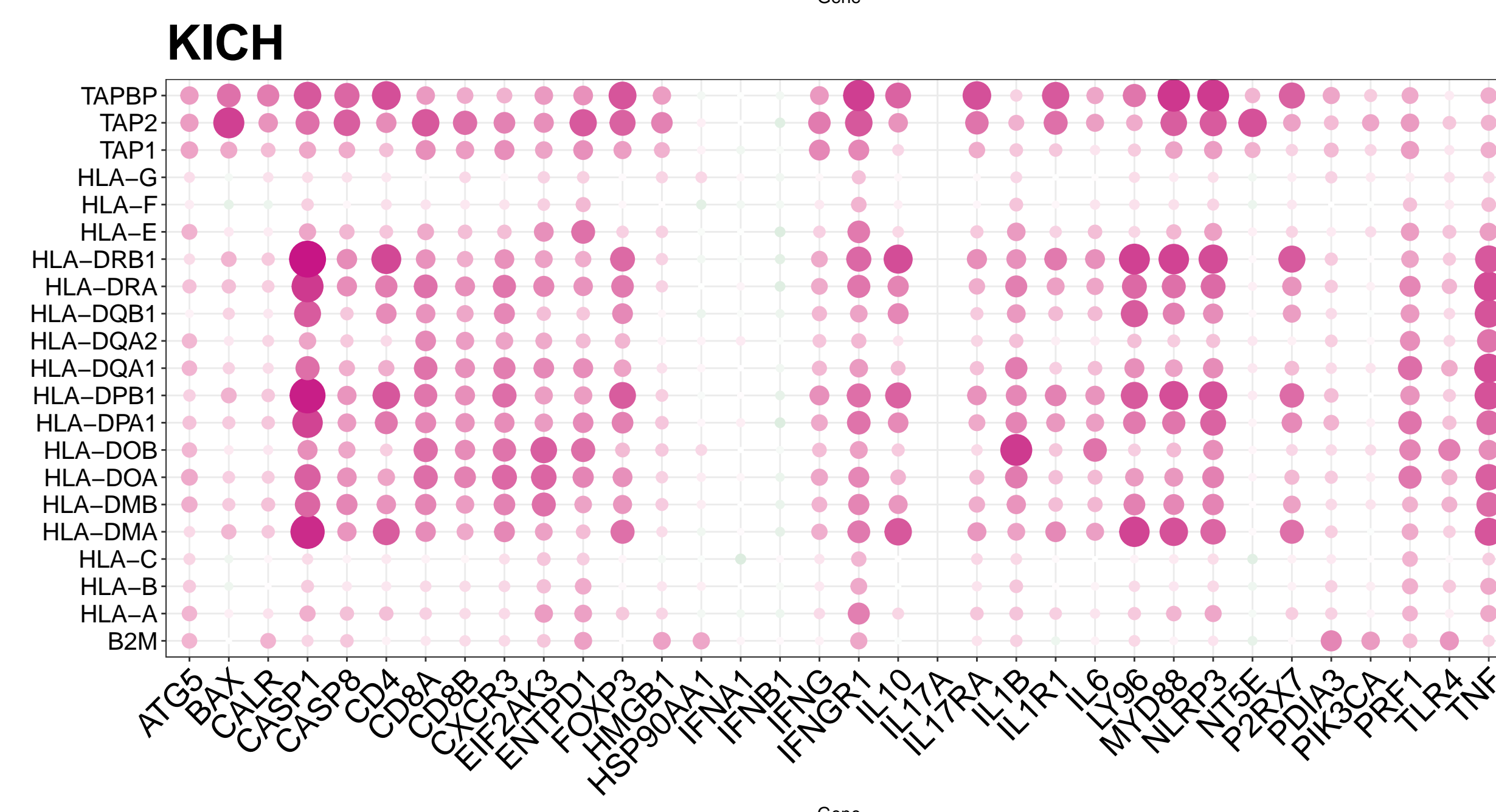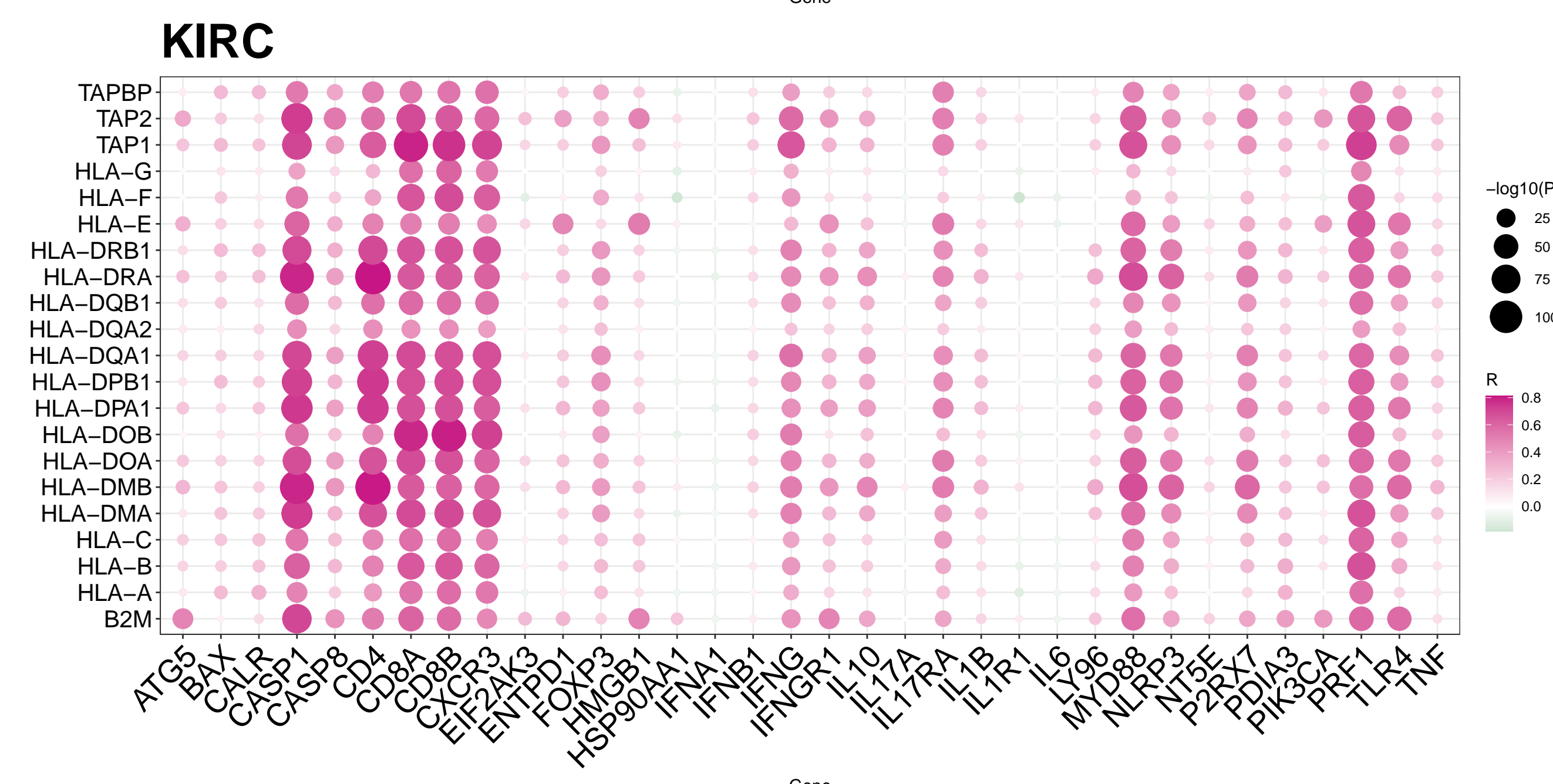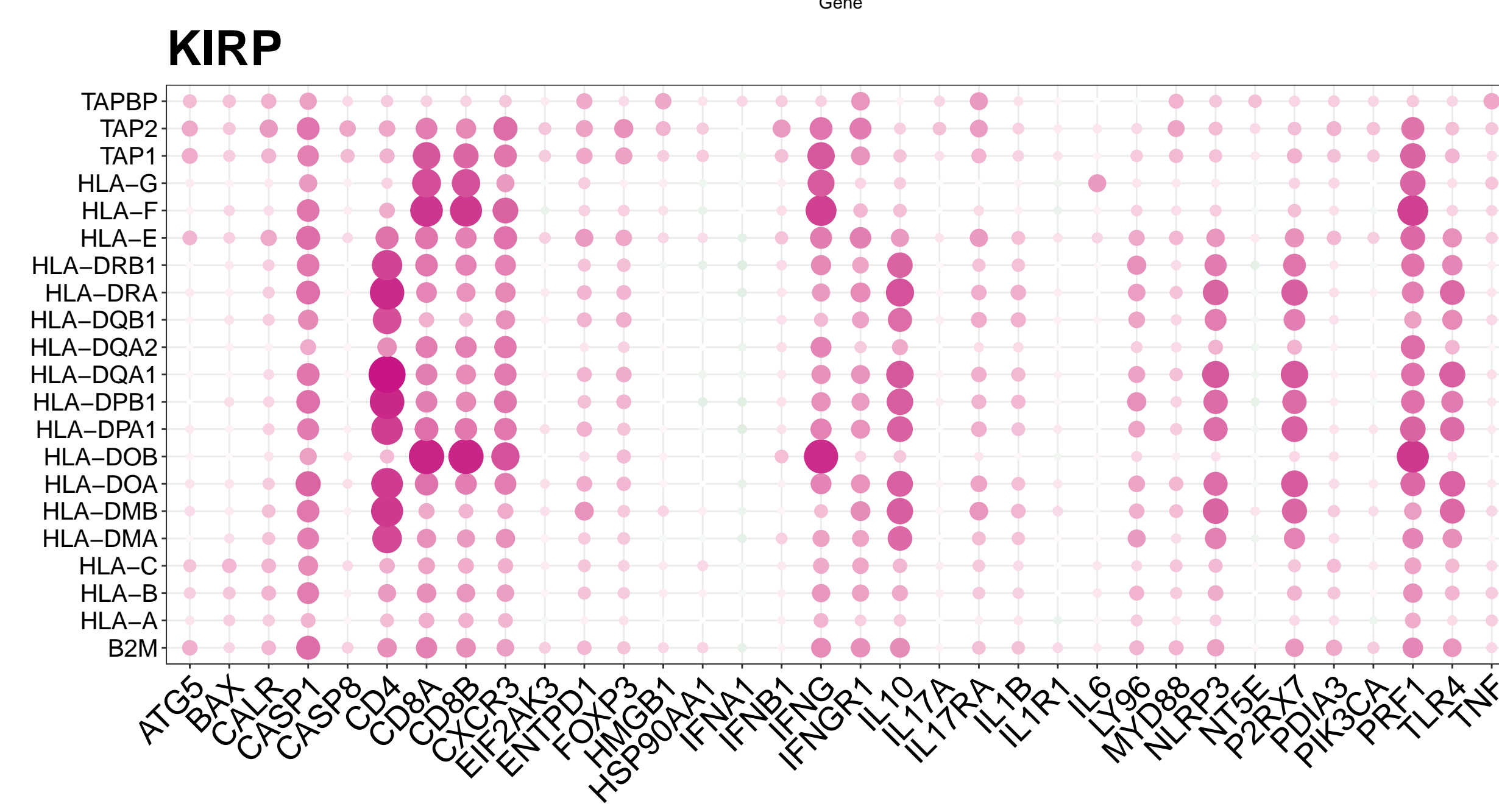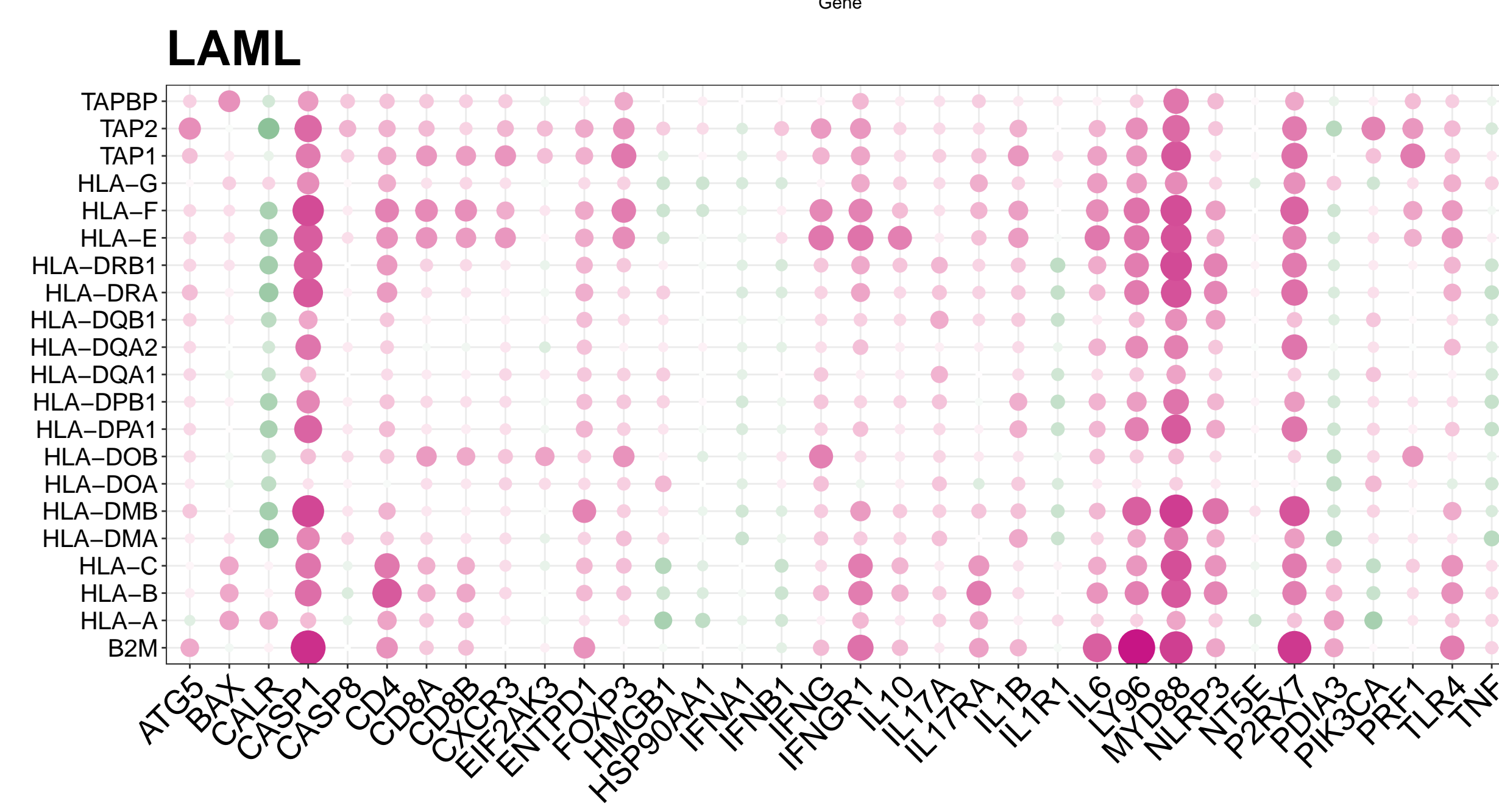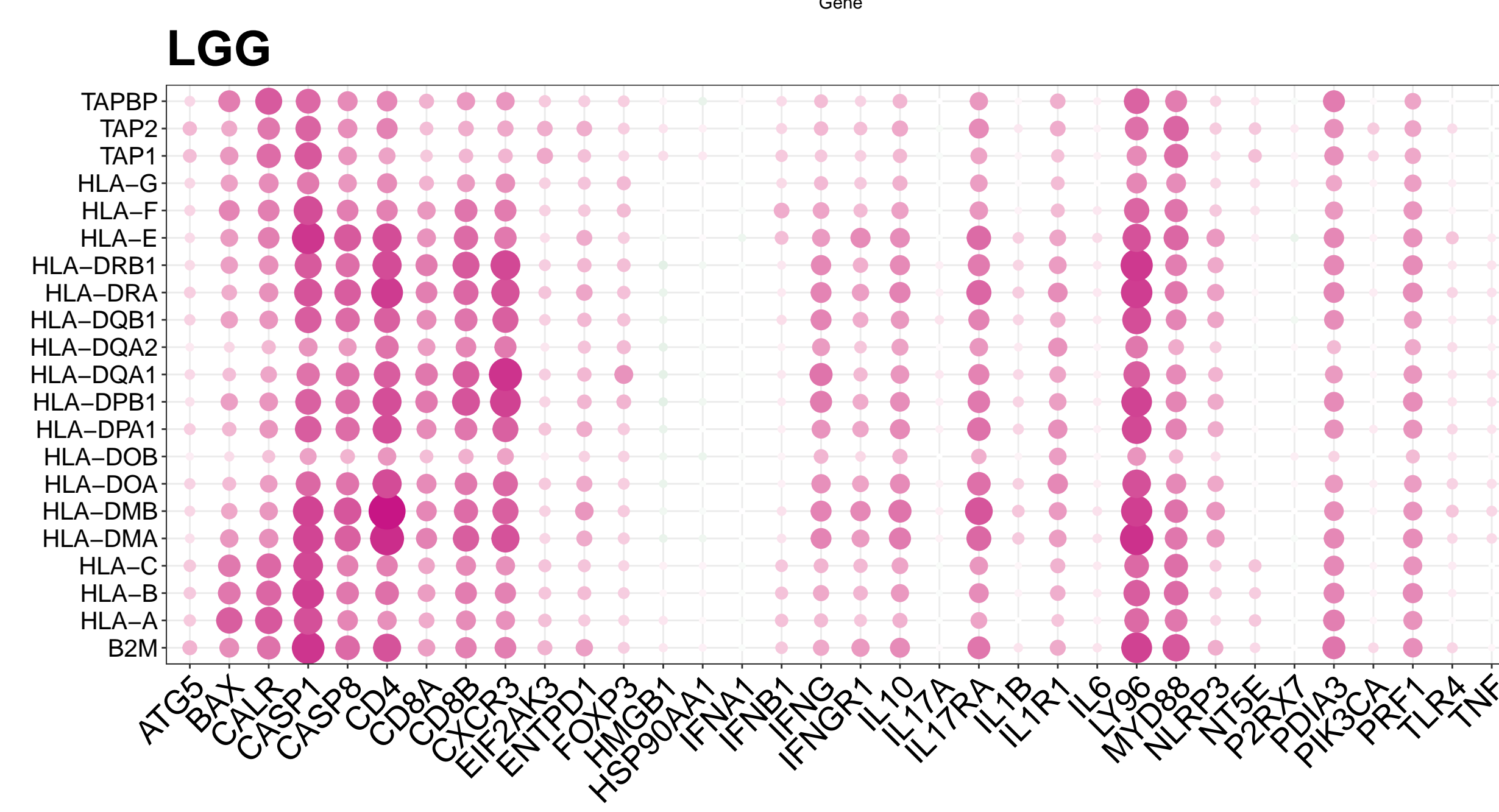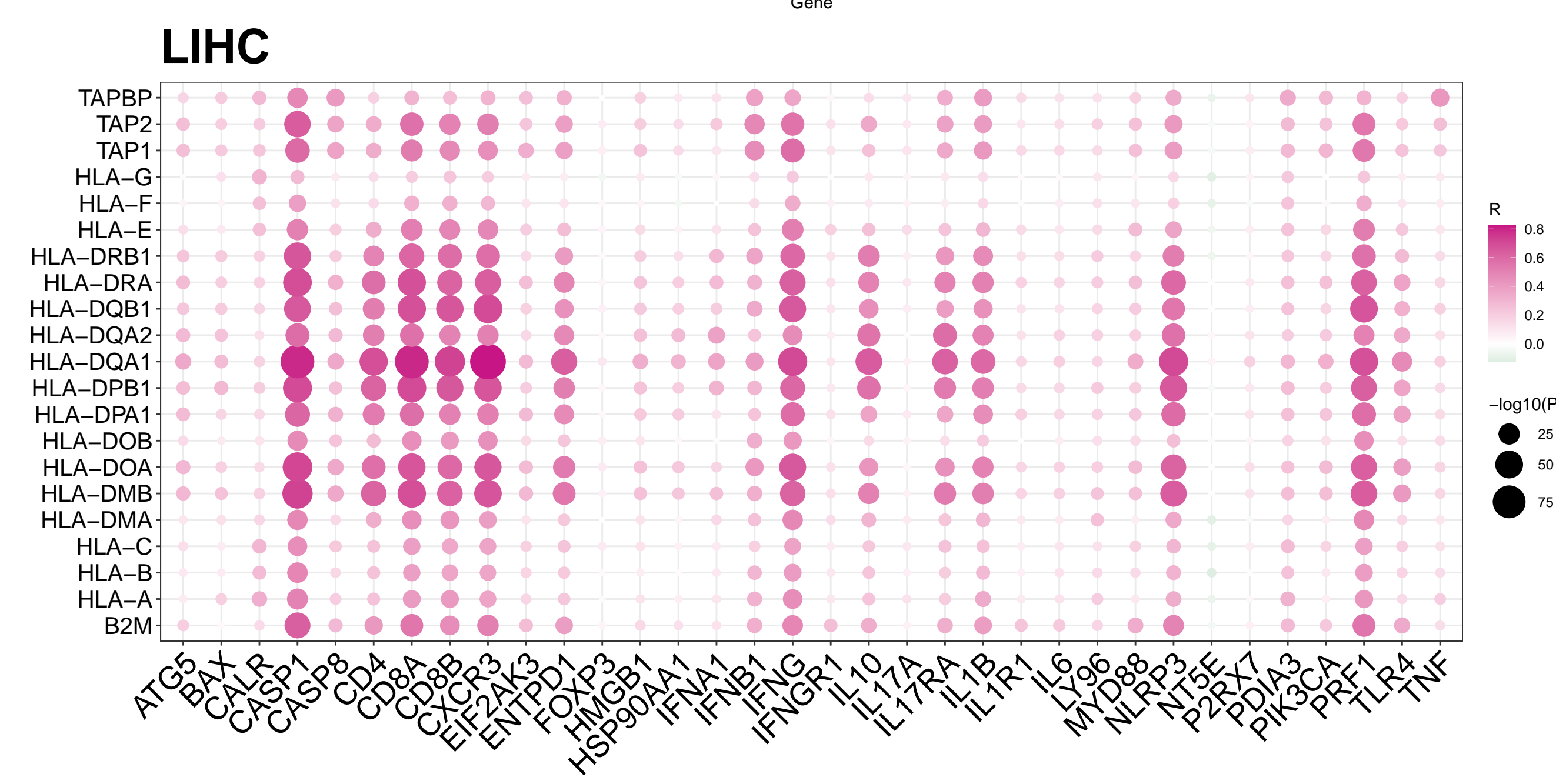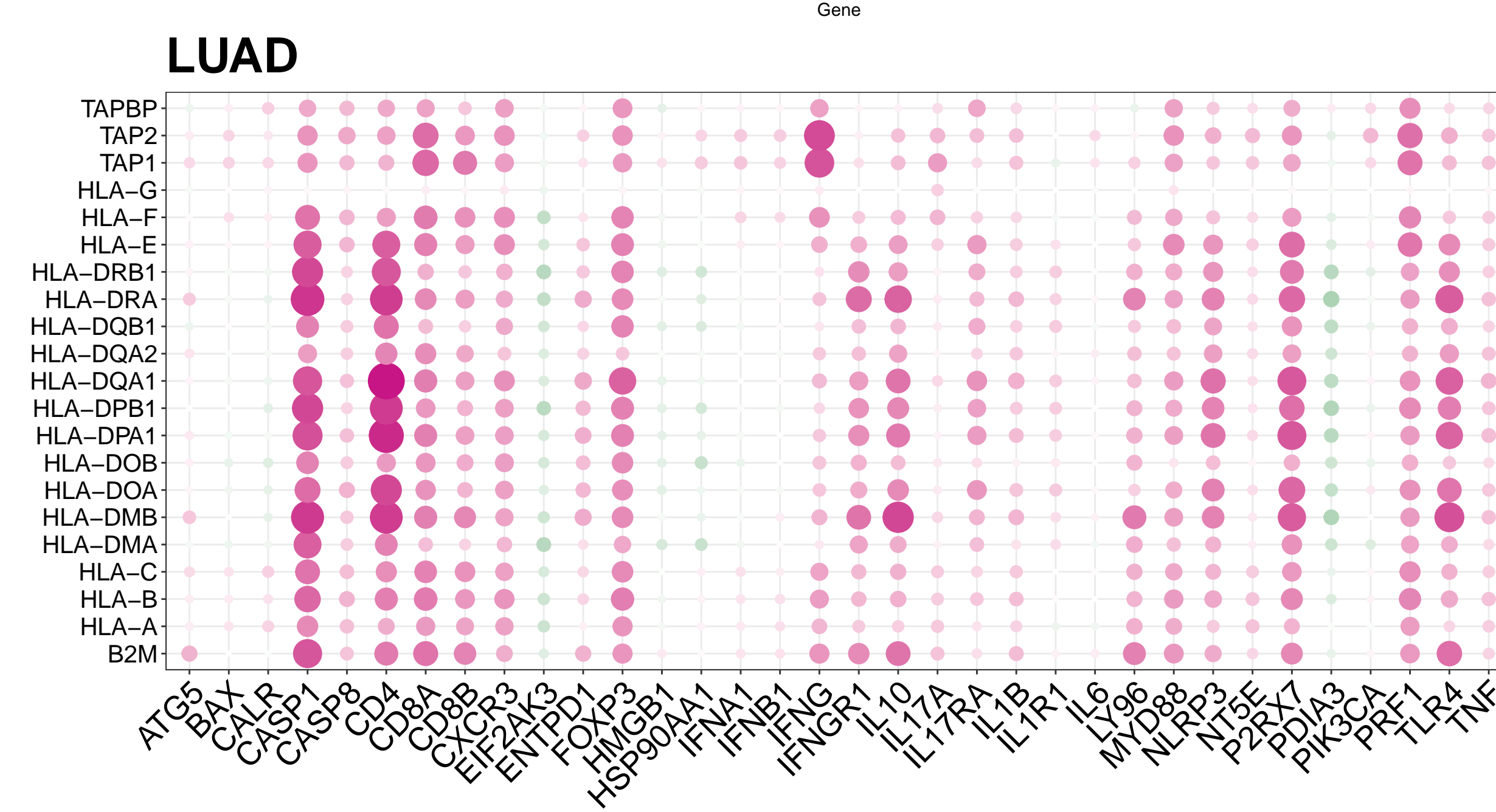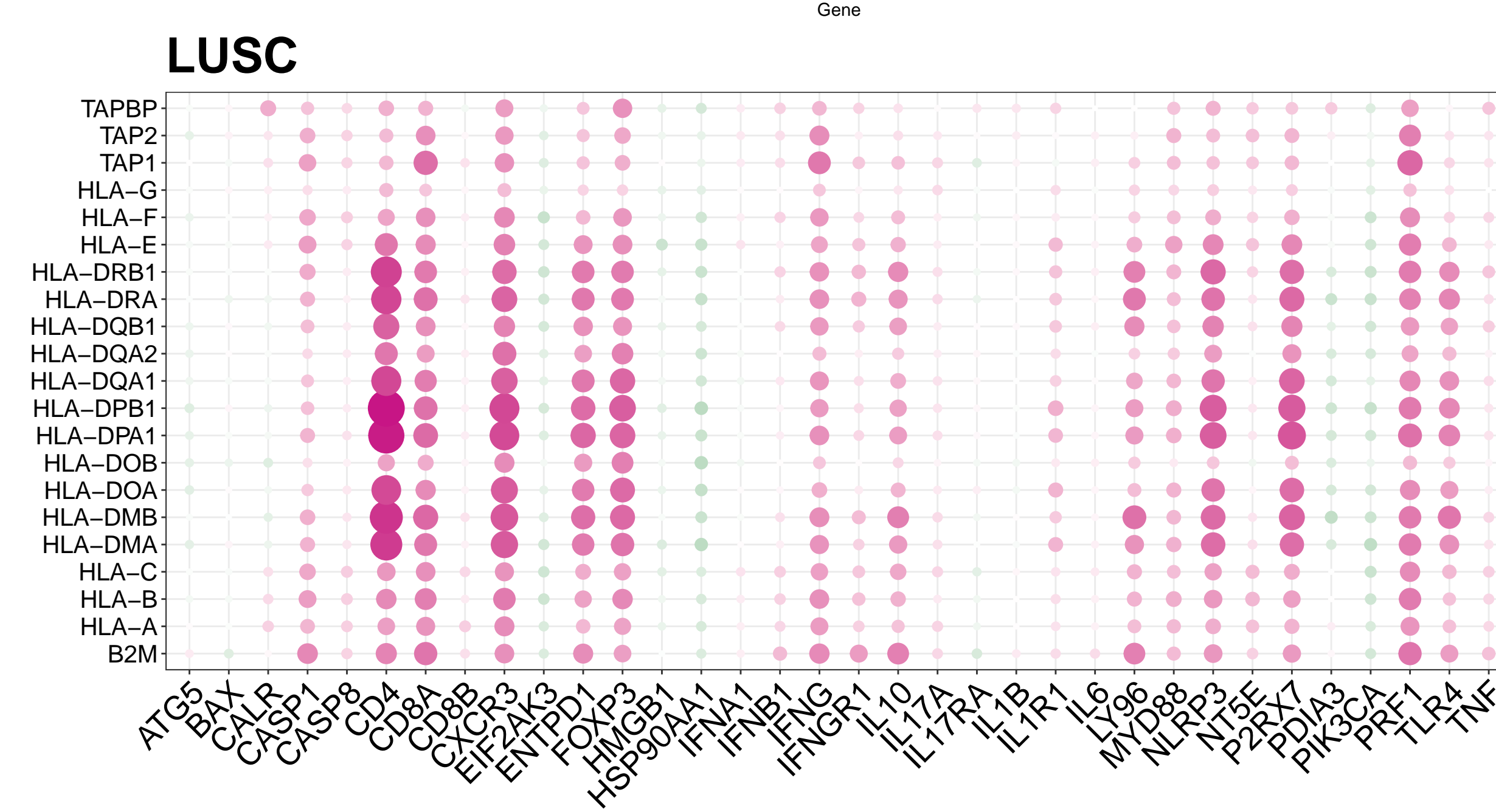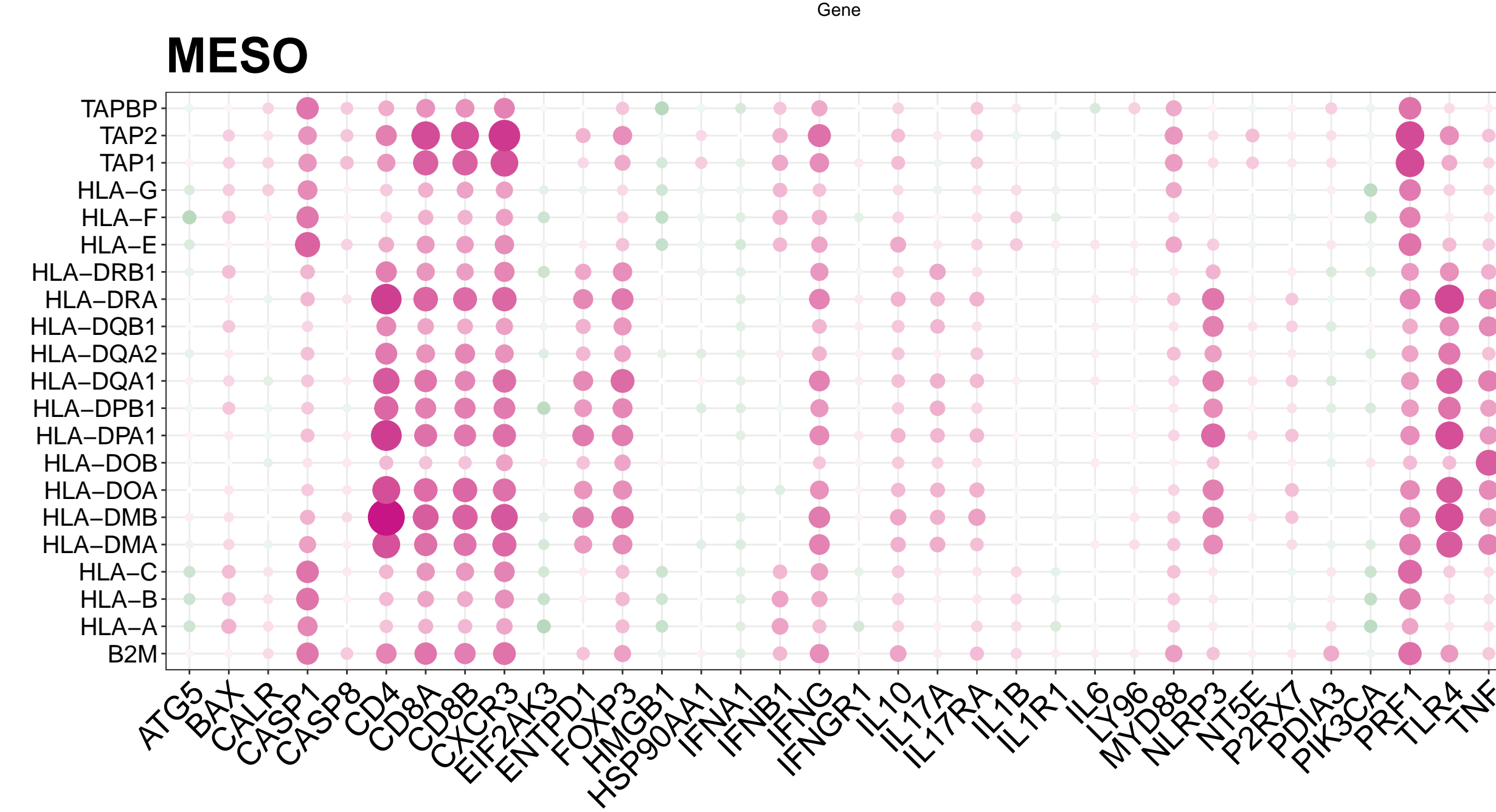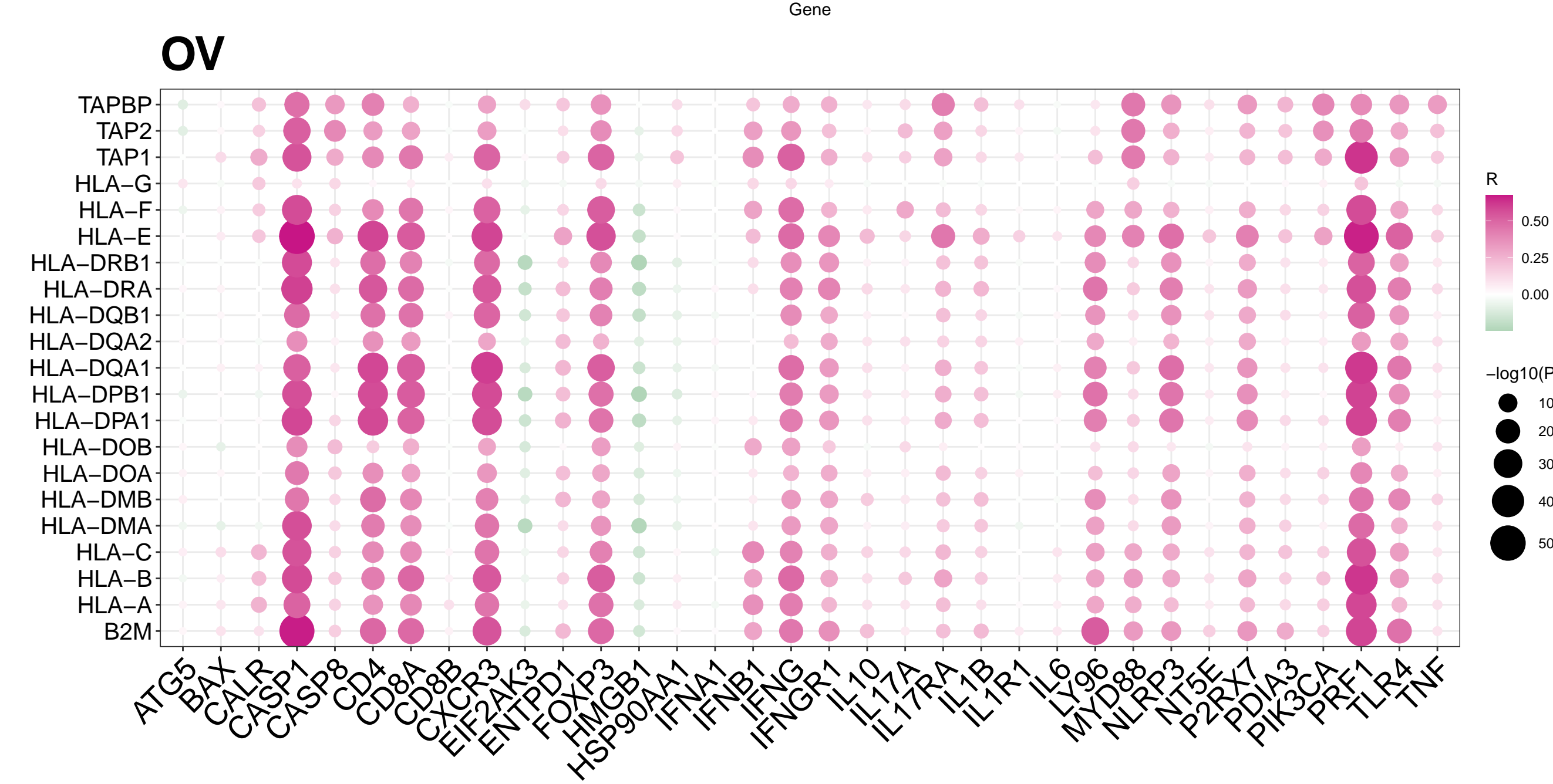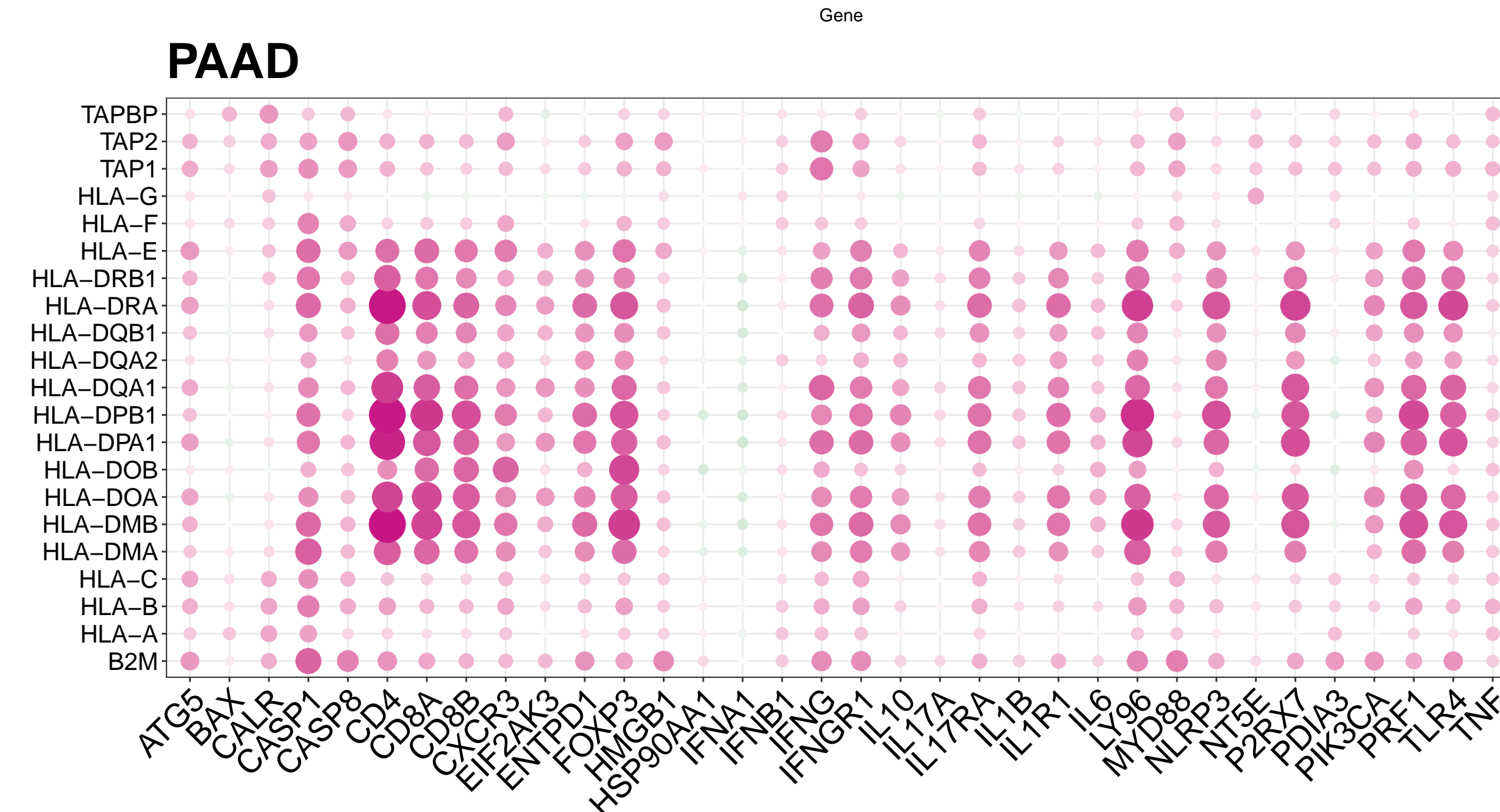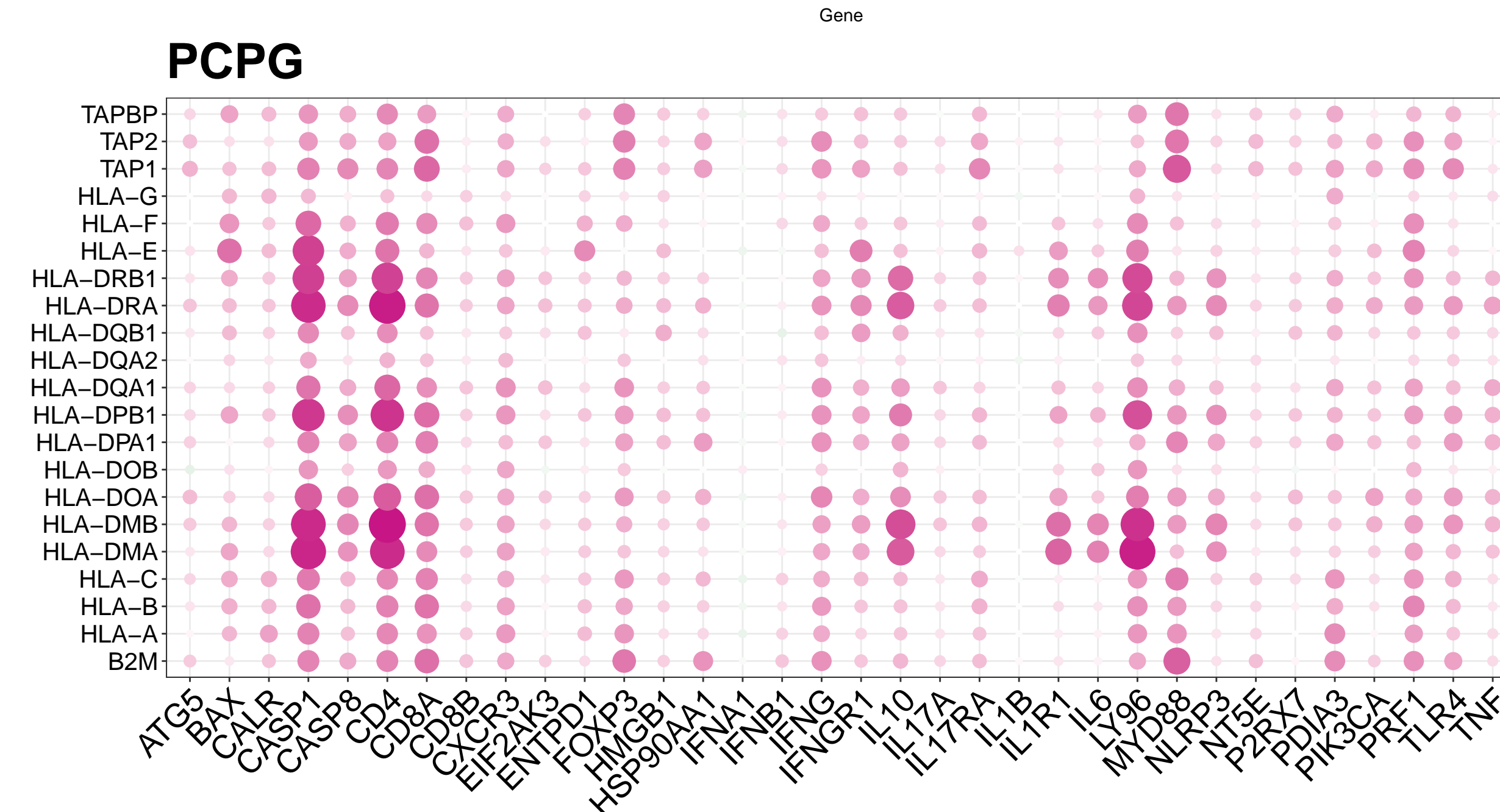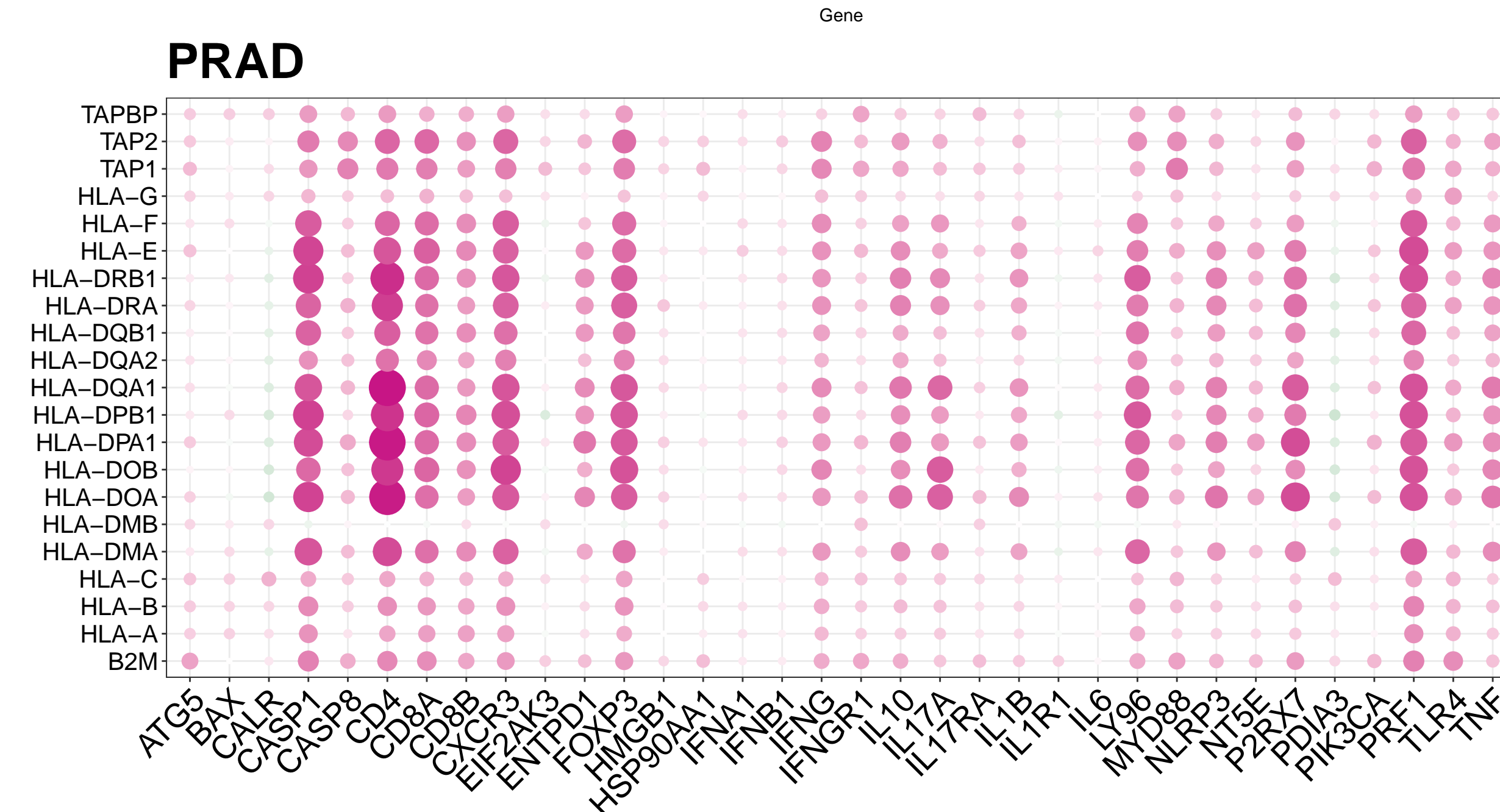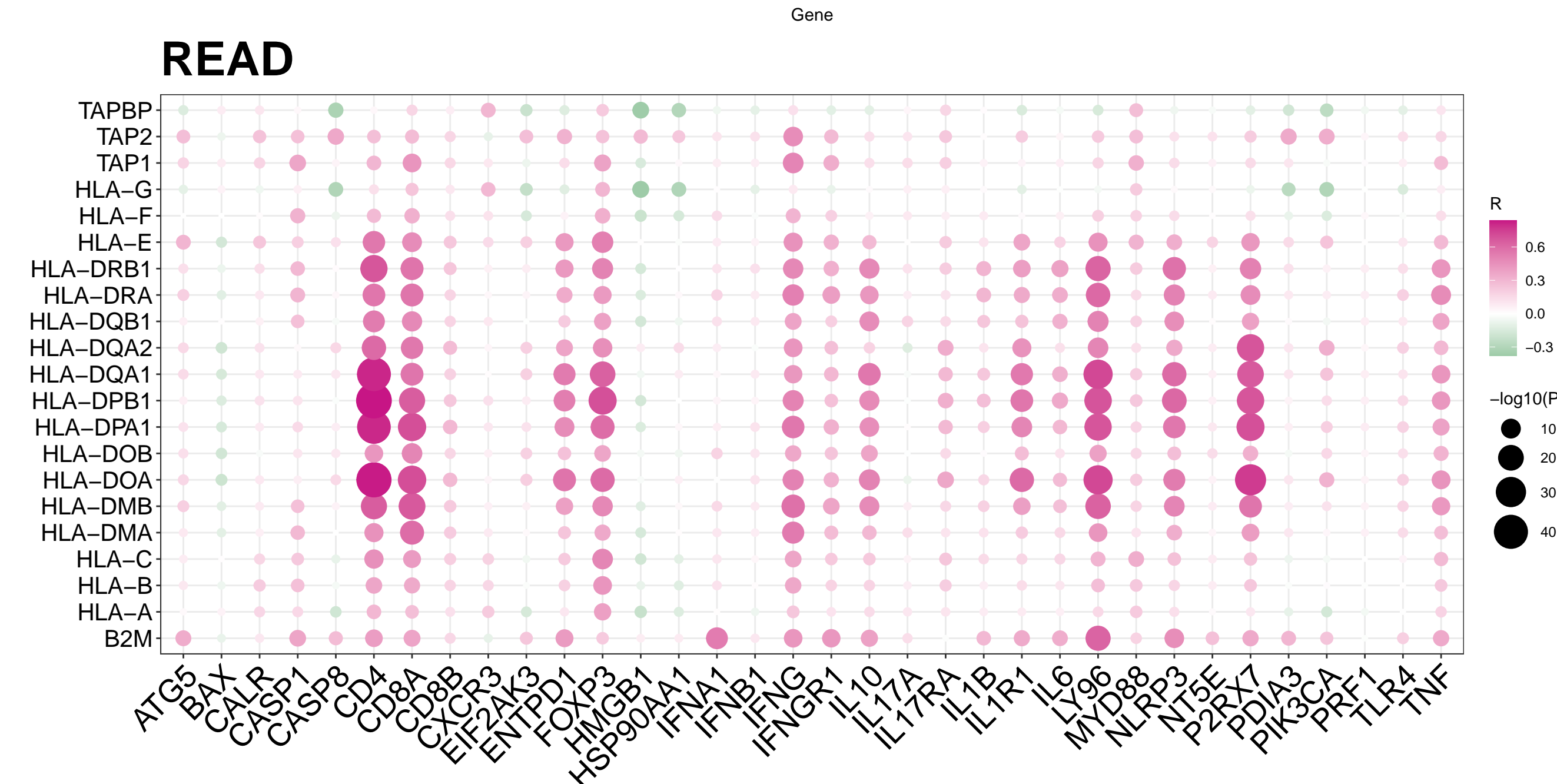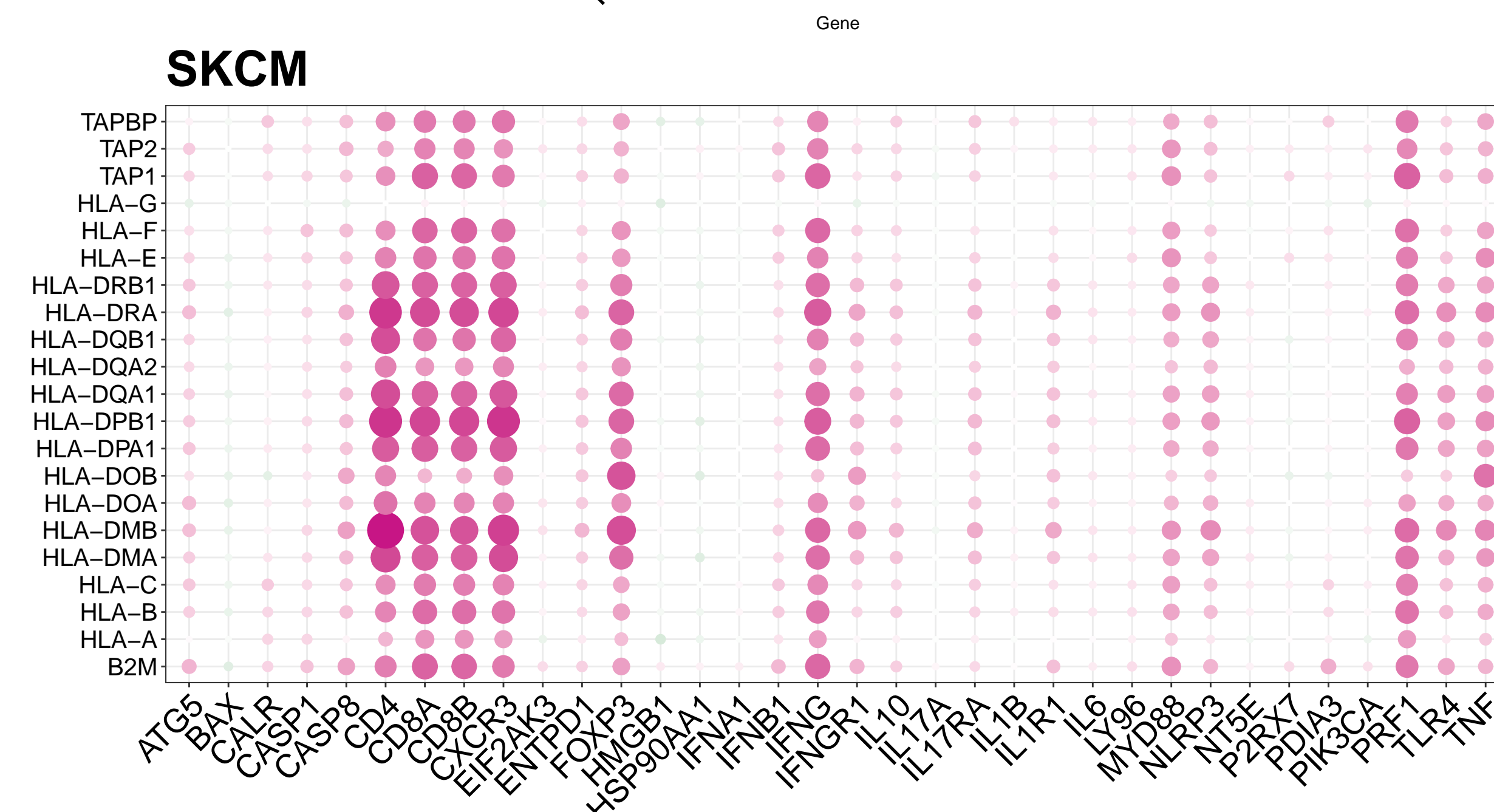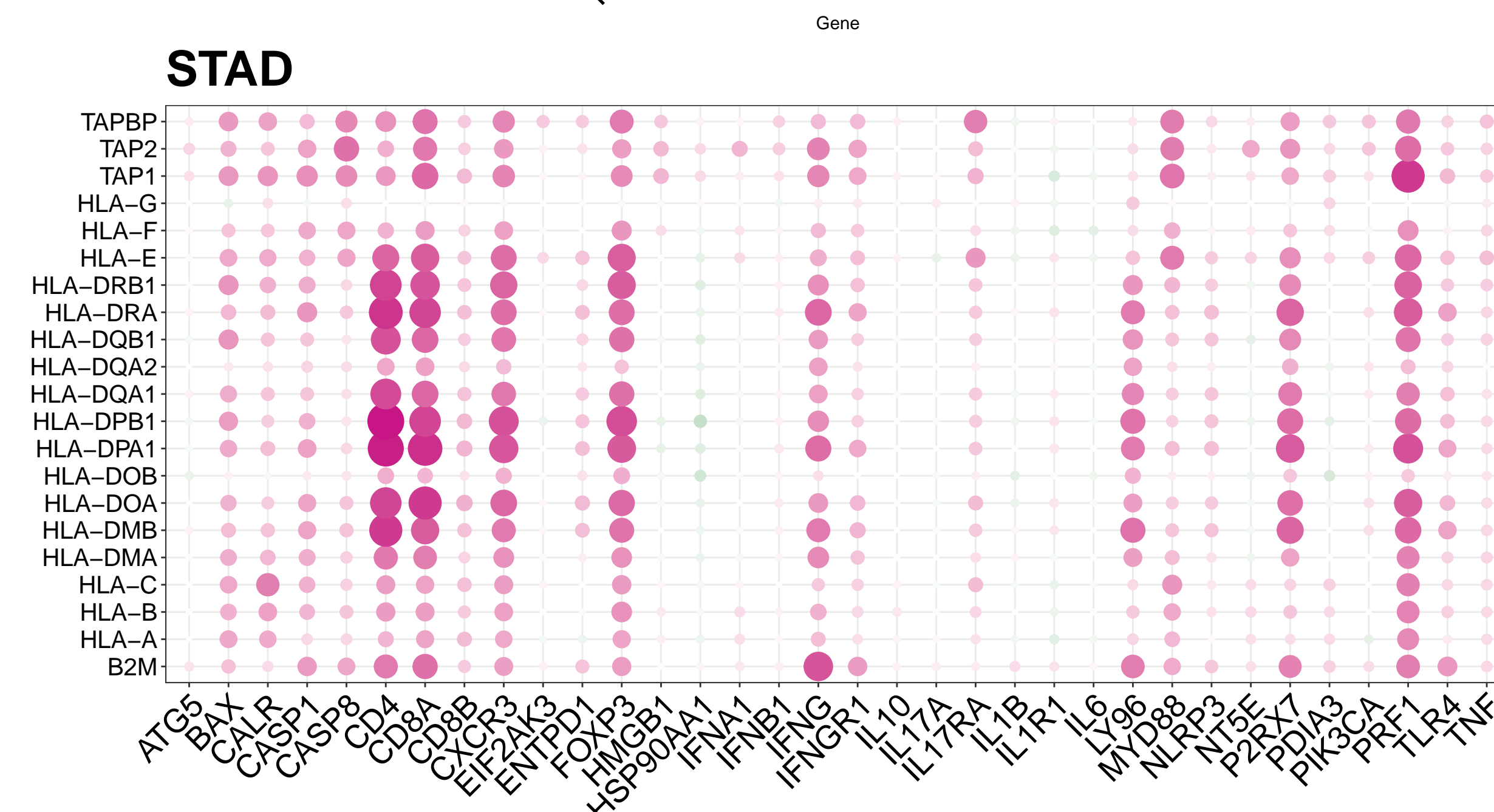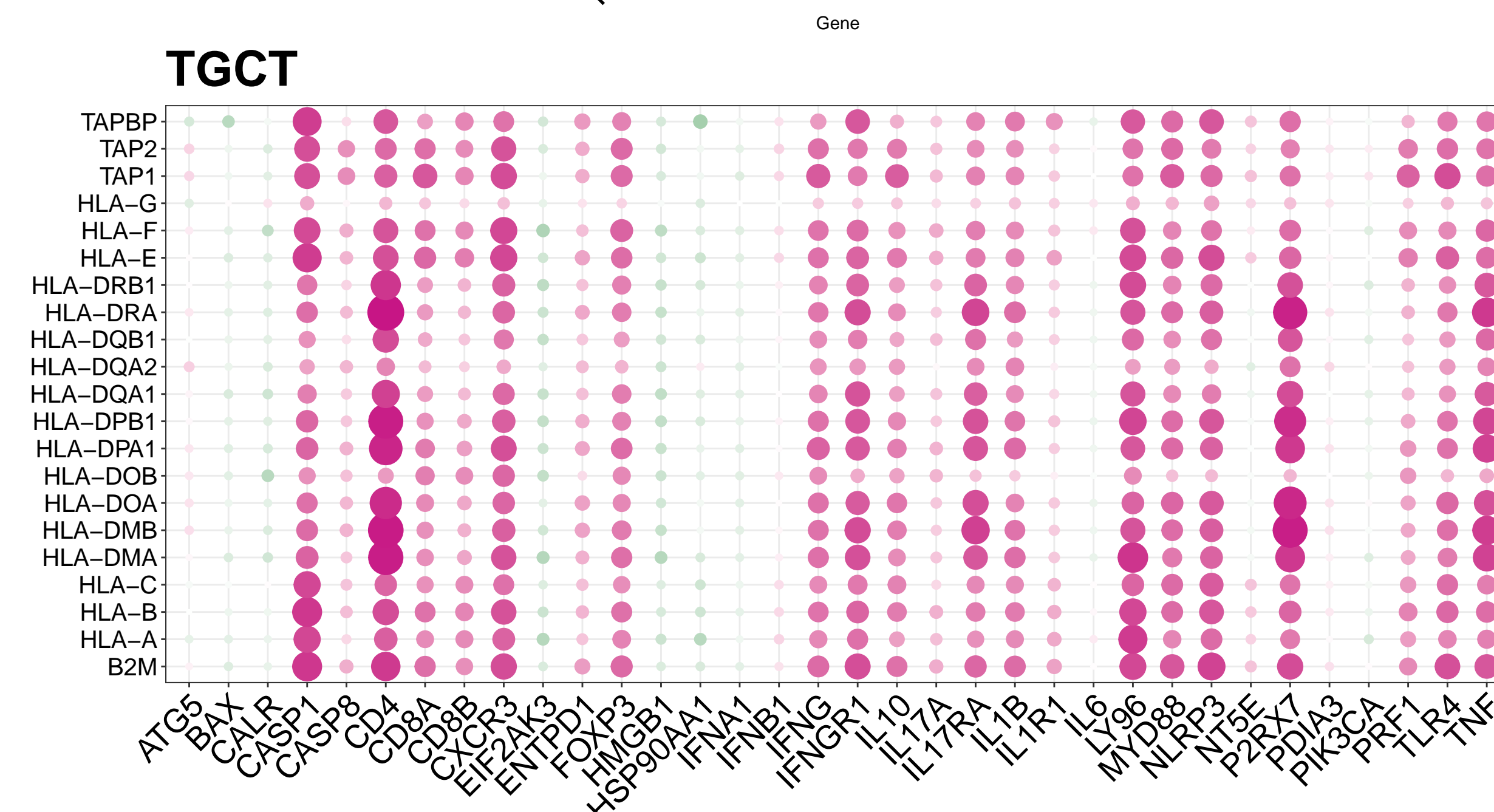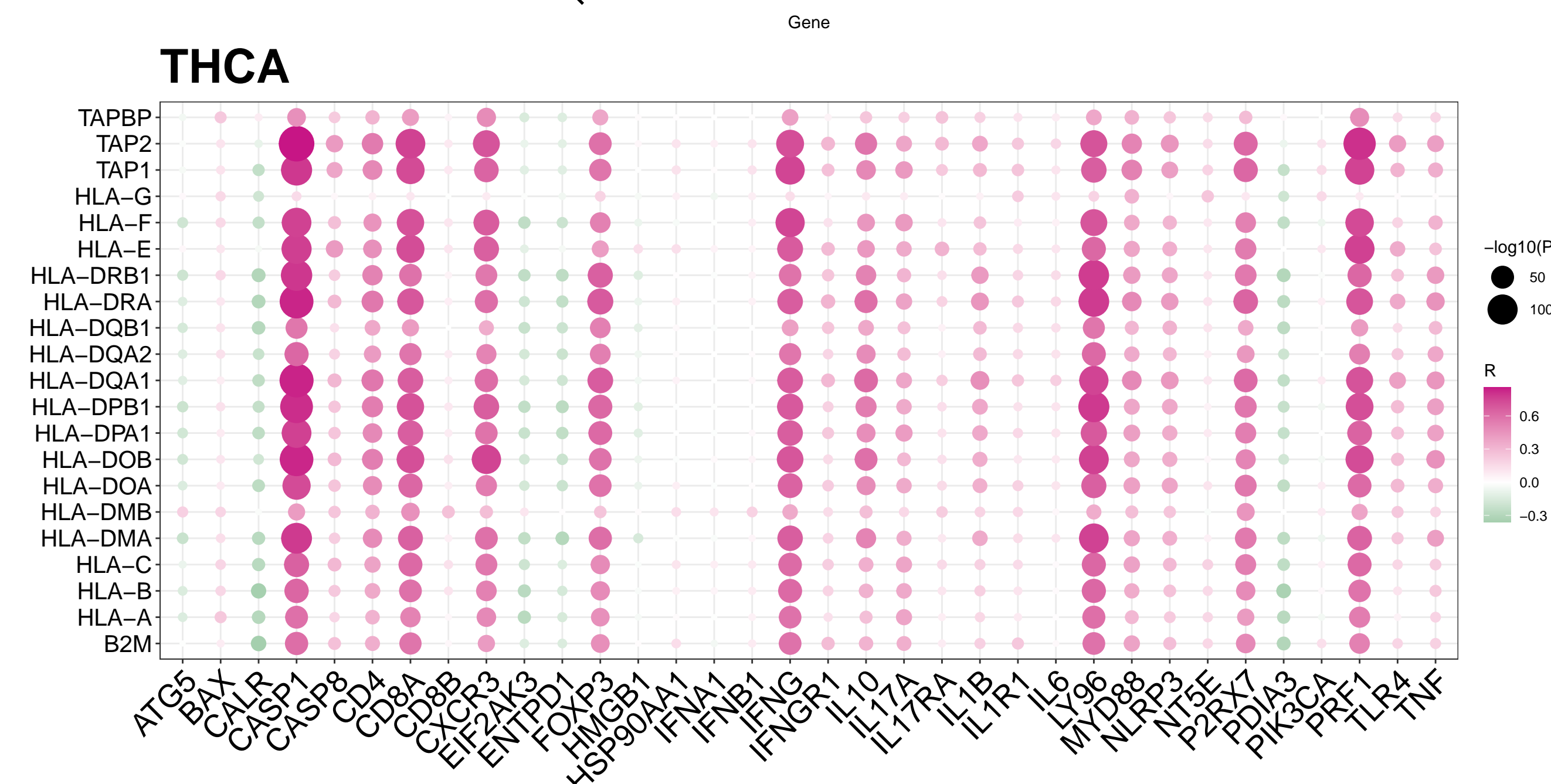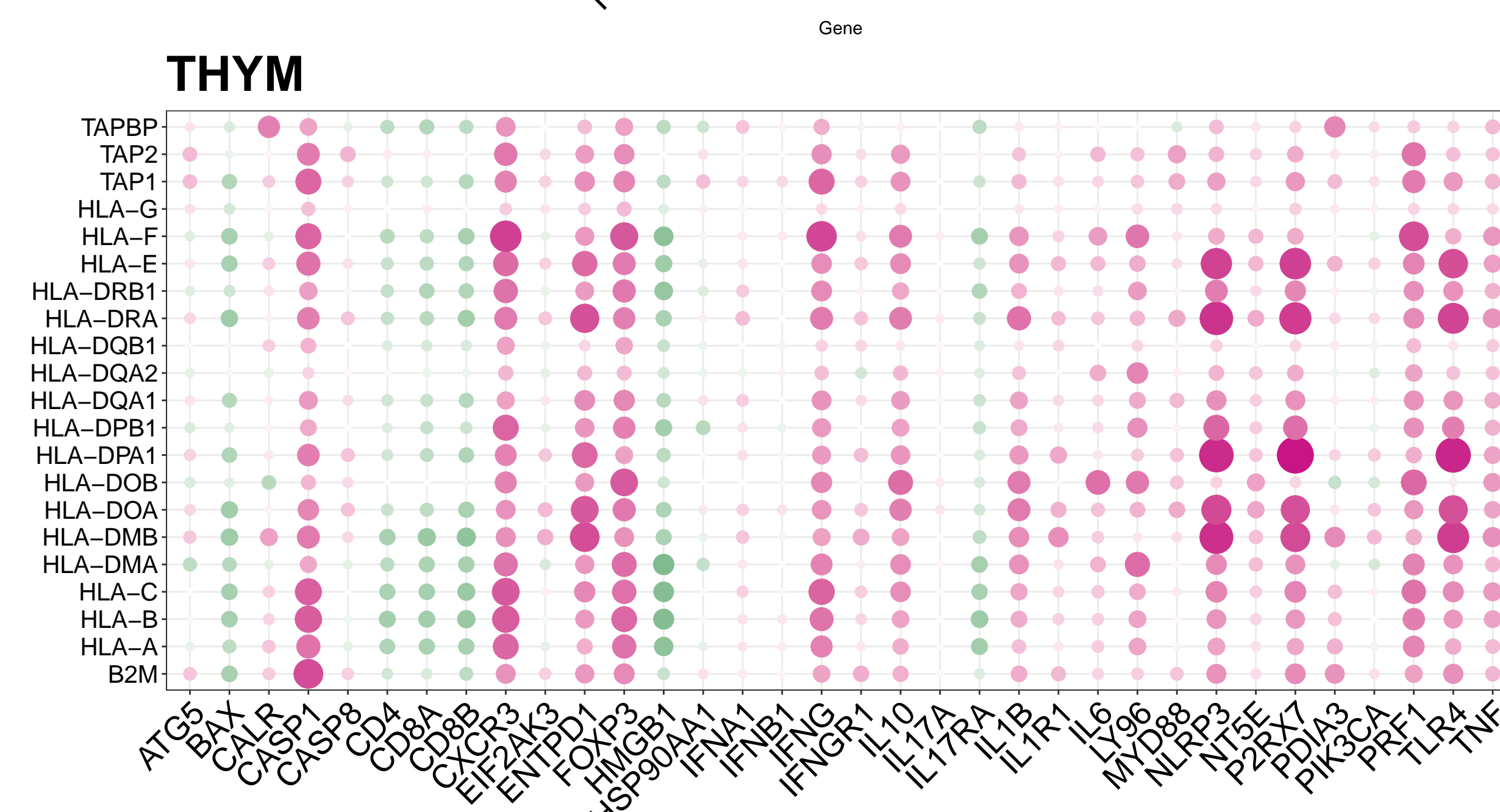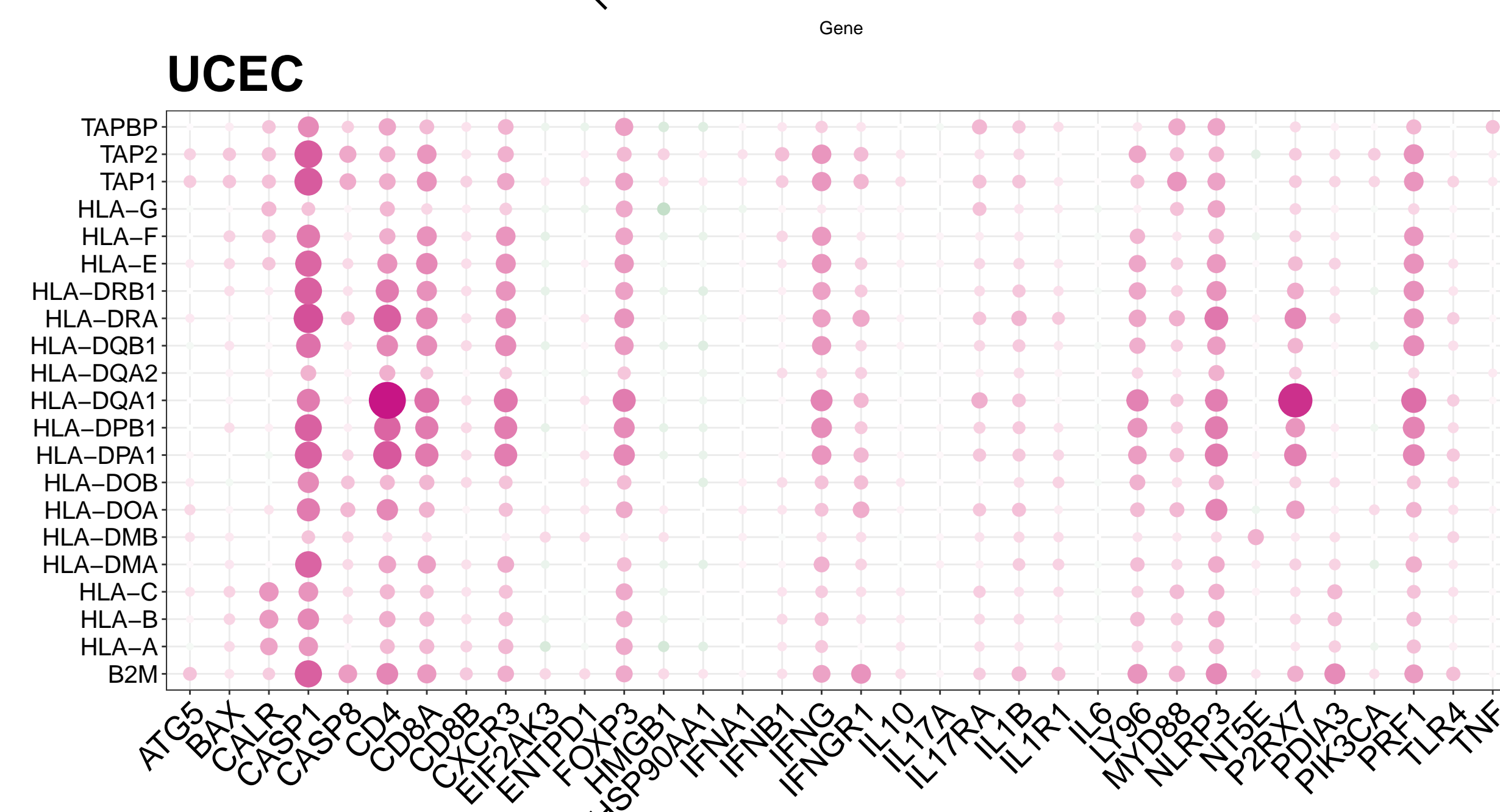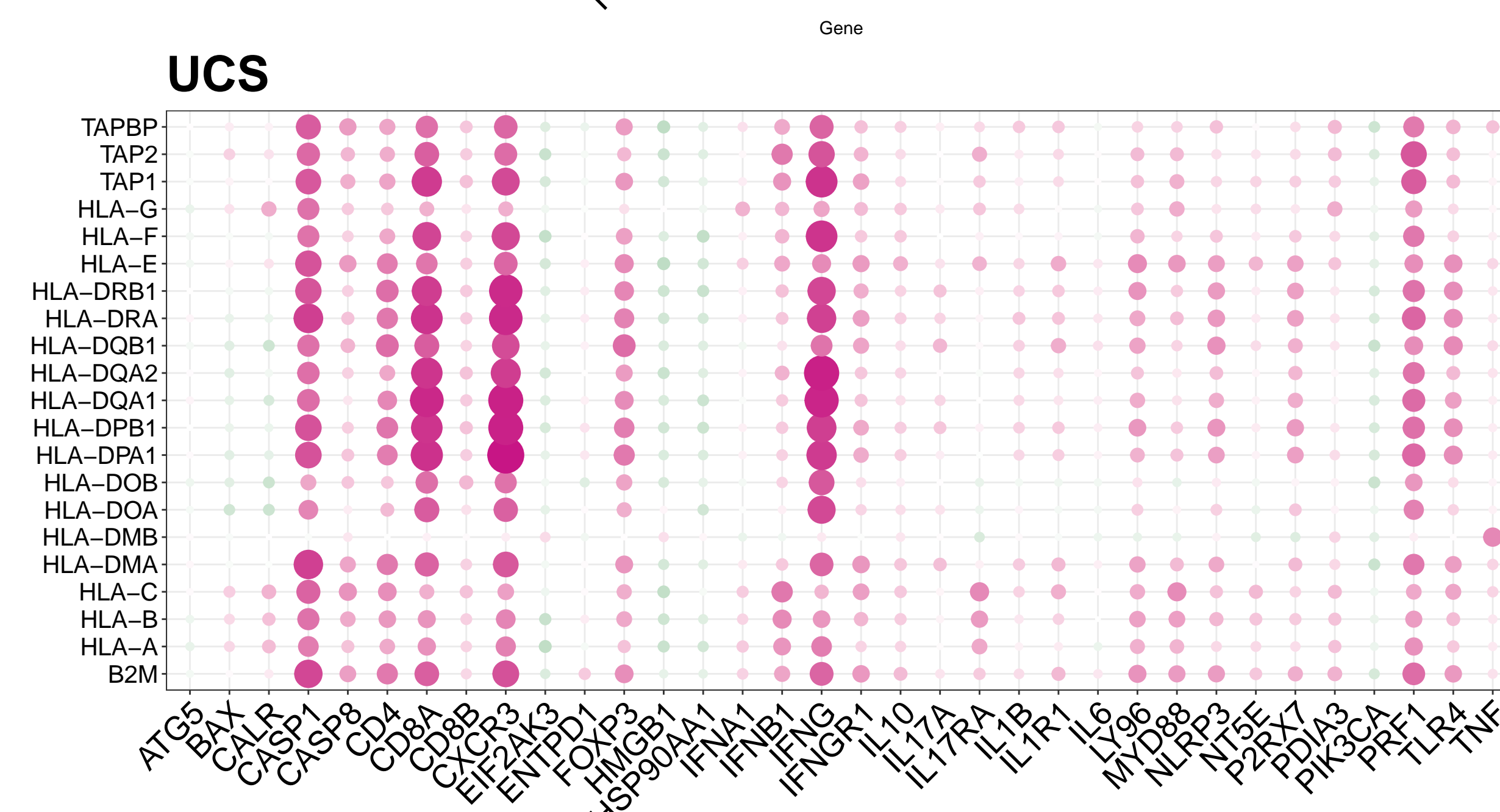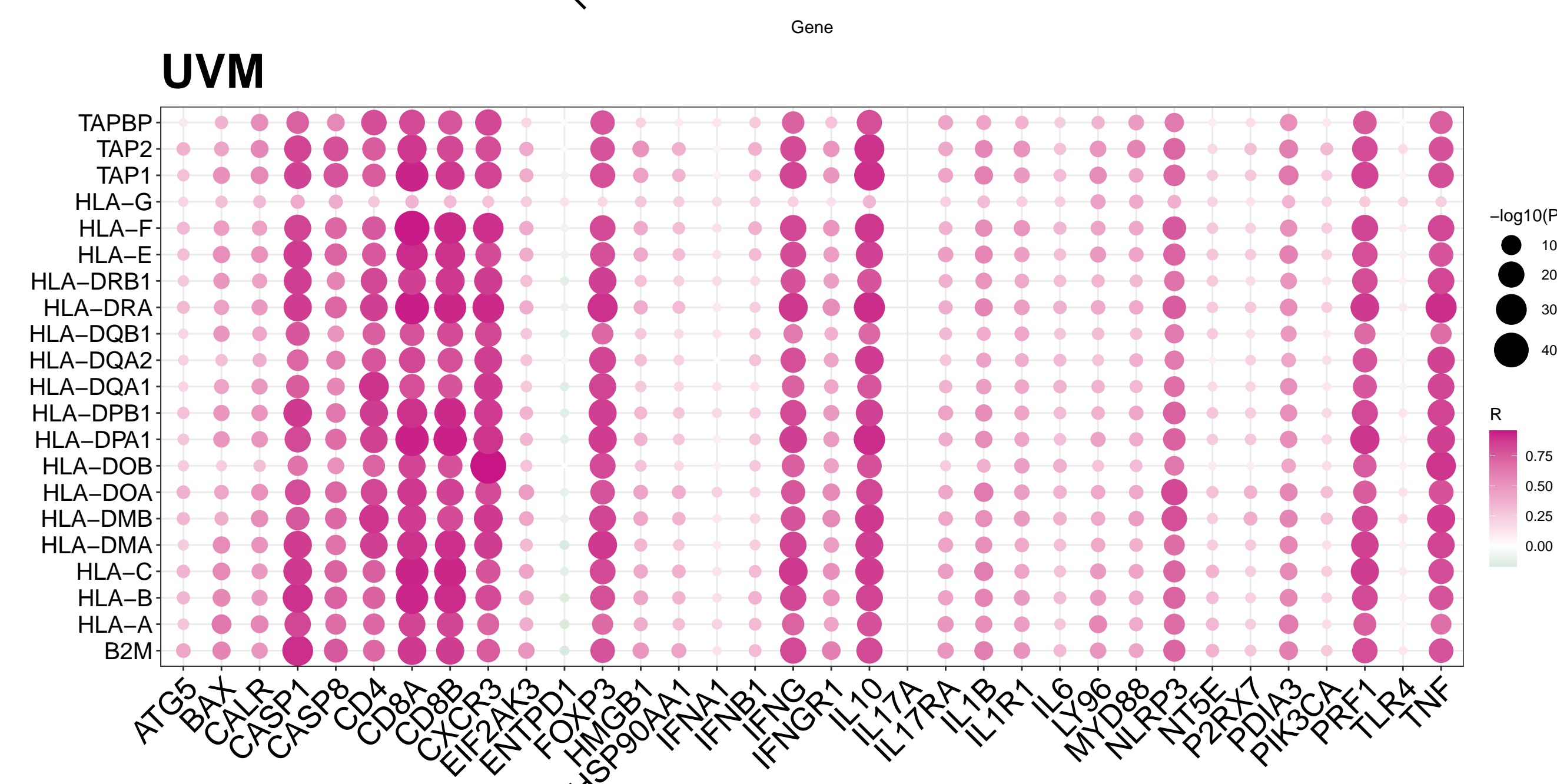

Supplement: Supplementary file 1 [file cimb-47-00812-s001.zip › cimb-3868671-supplementary/Supplementary_0930/Supplementary Figure s16-MHC.pdf]

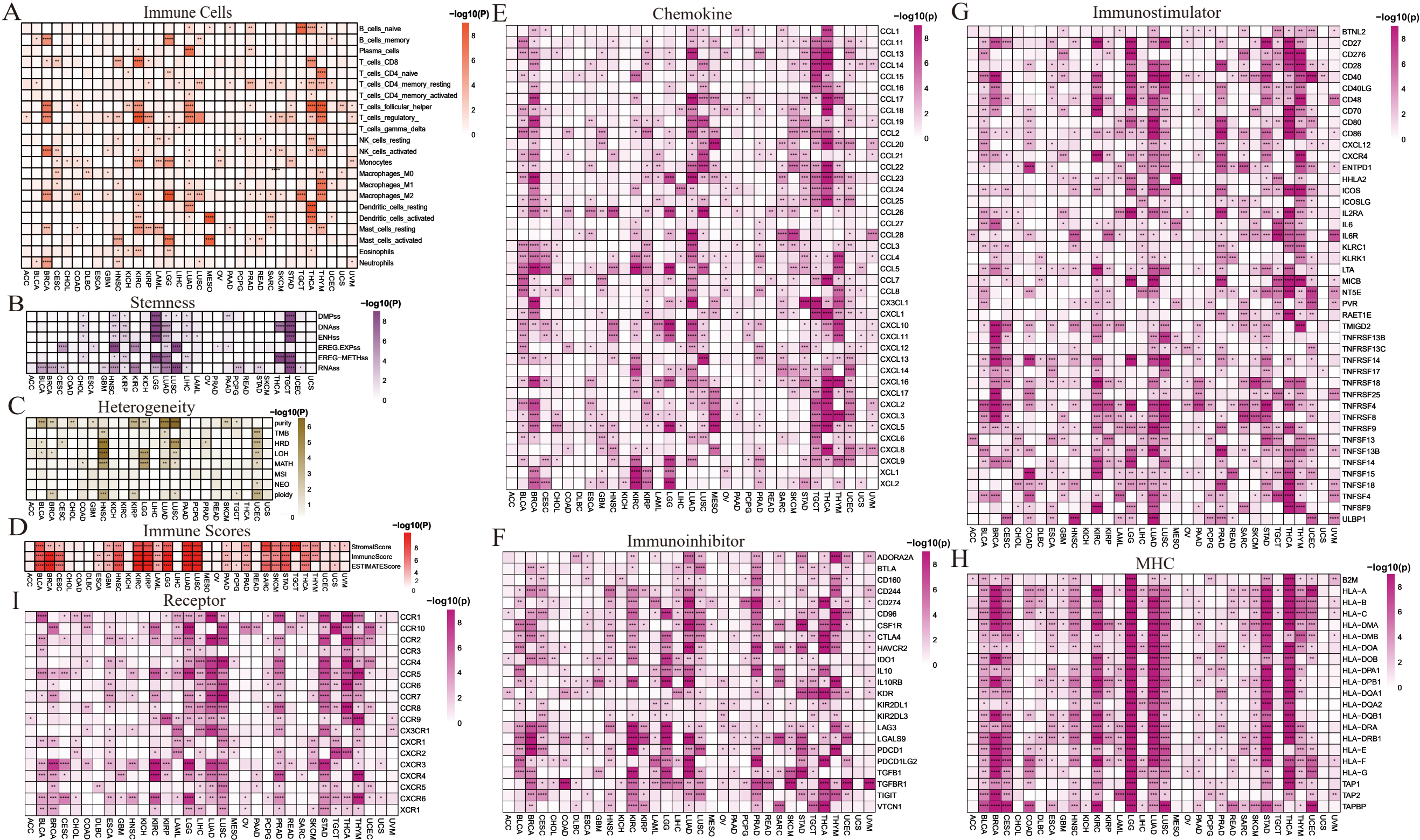

Supplement: Supplementary file 1 [file cimb-47-00812-s001.zip › cimb-3868671-supplementary/Supplementary_0930/Supplementary Figure s18.pdf]

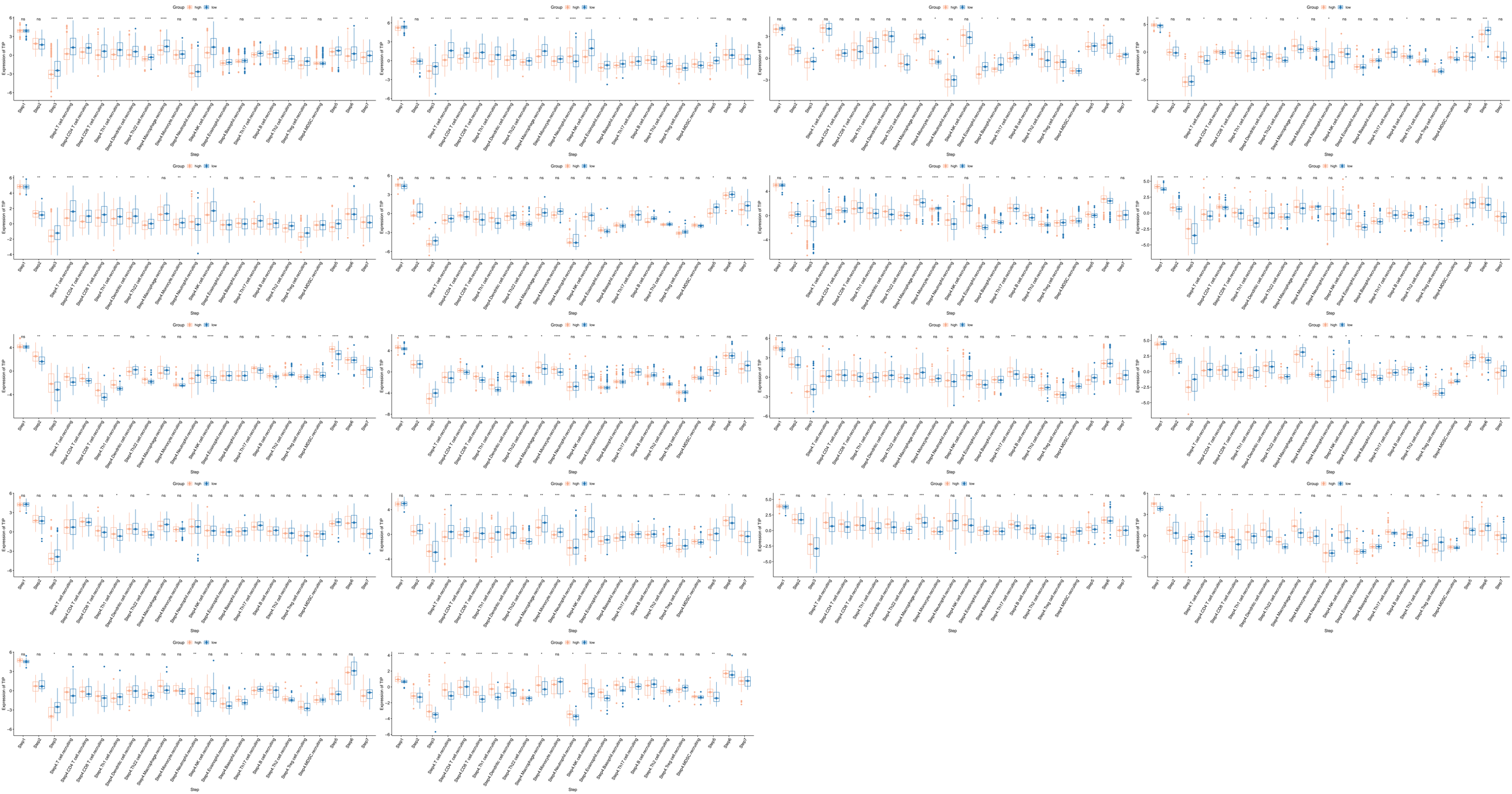

Supplement: Supplementary file 1 [file cimb-47-00812-s001.zip › cimb-3868671-supplementary/Supplementary_0930/Supplementary Figure s19.pdf]

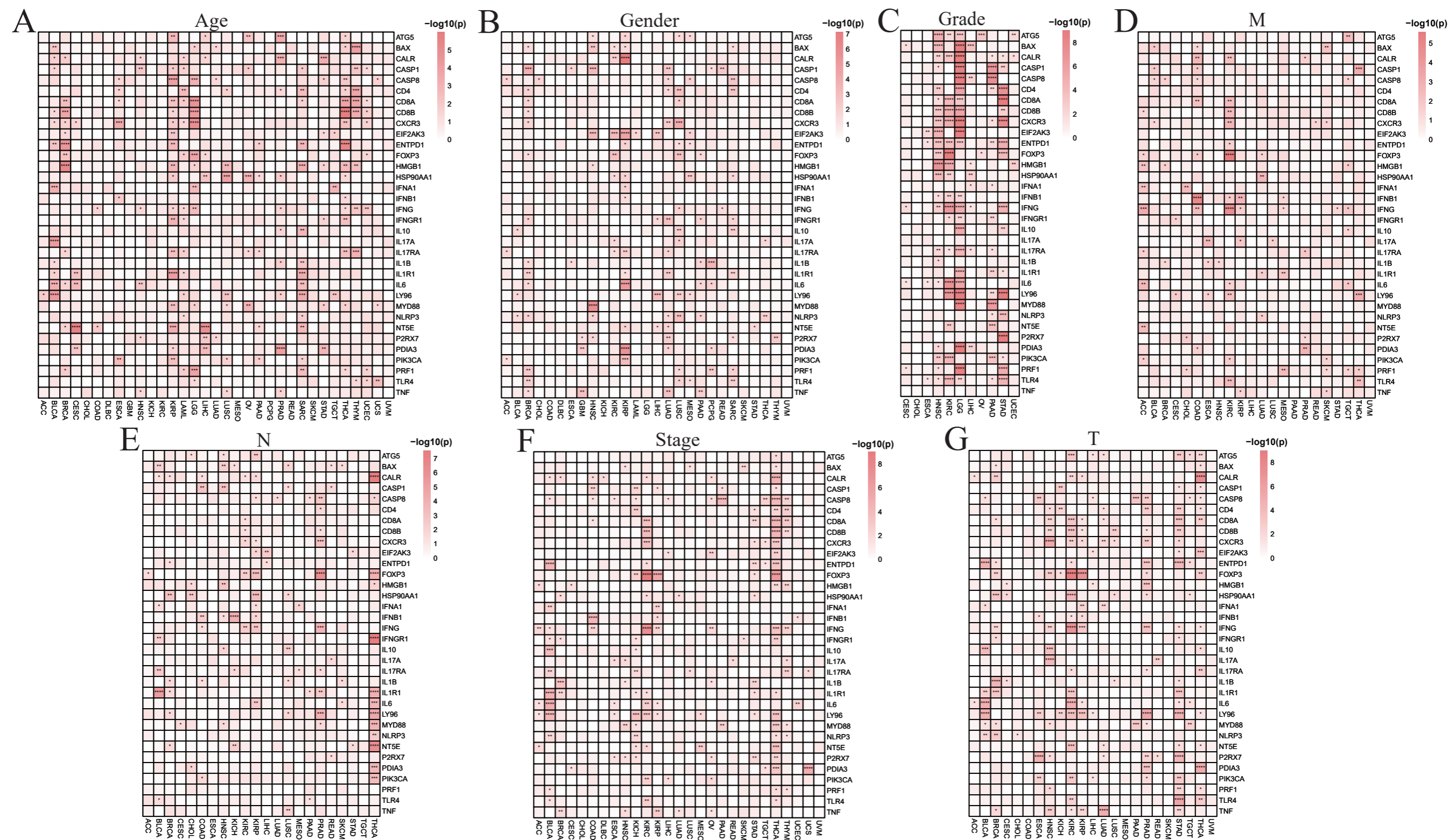

Supplement: Supplementary file 1 [file cimb-47-00812-s001.zip › cimb-3868671-supplementary/Supplementary_0930/Supplementary Figure s2.pdf]

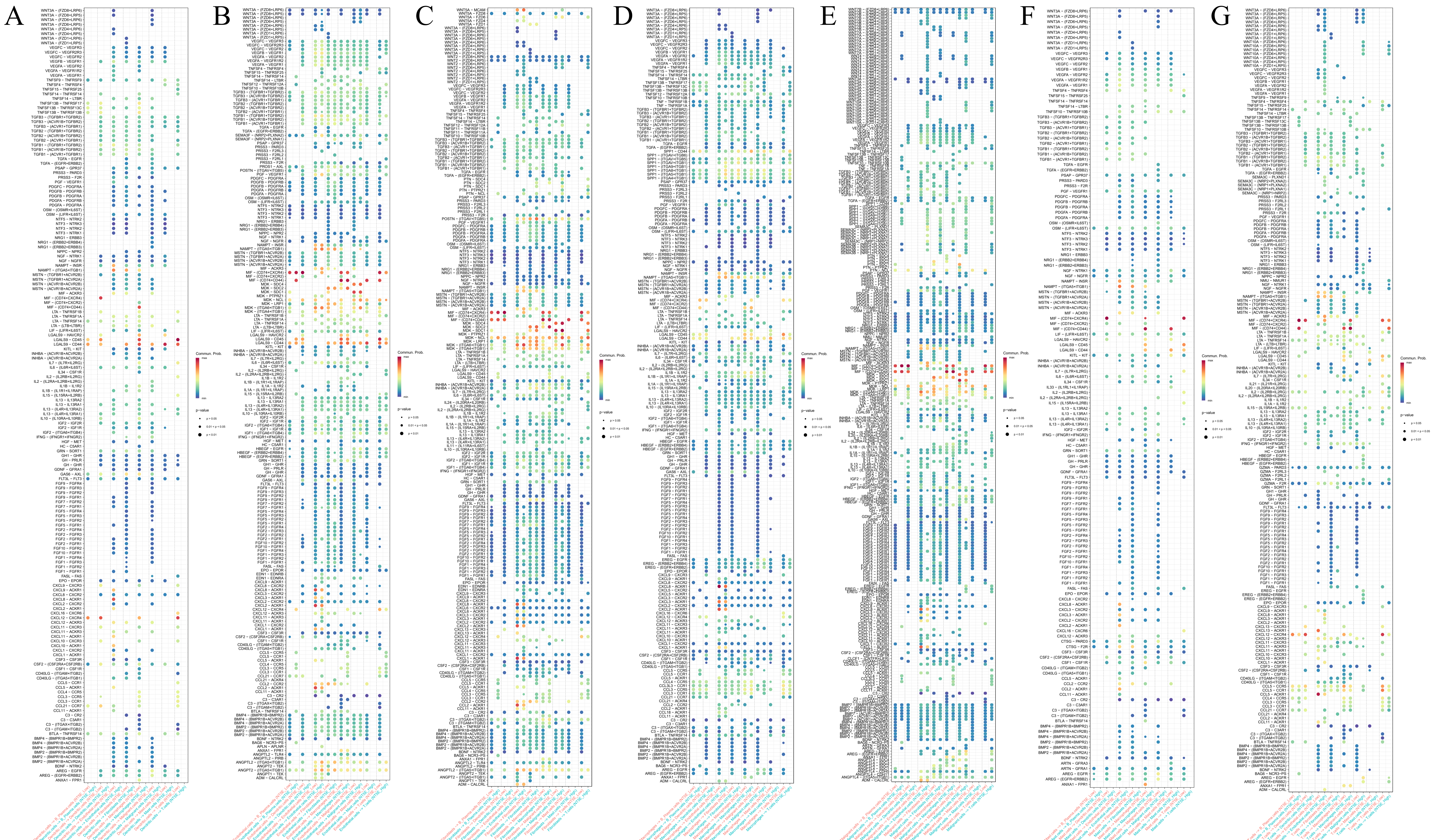

Supplement: Supplementary file 1 [file cimb-47-00812-s001.zip › cimb-3868671-supplementary/Supplementary_0930/Supplementary Figure s21.pdf]

A

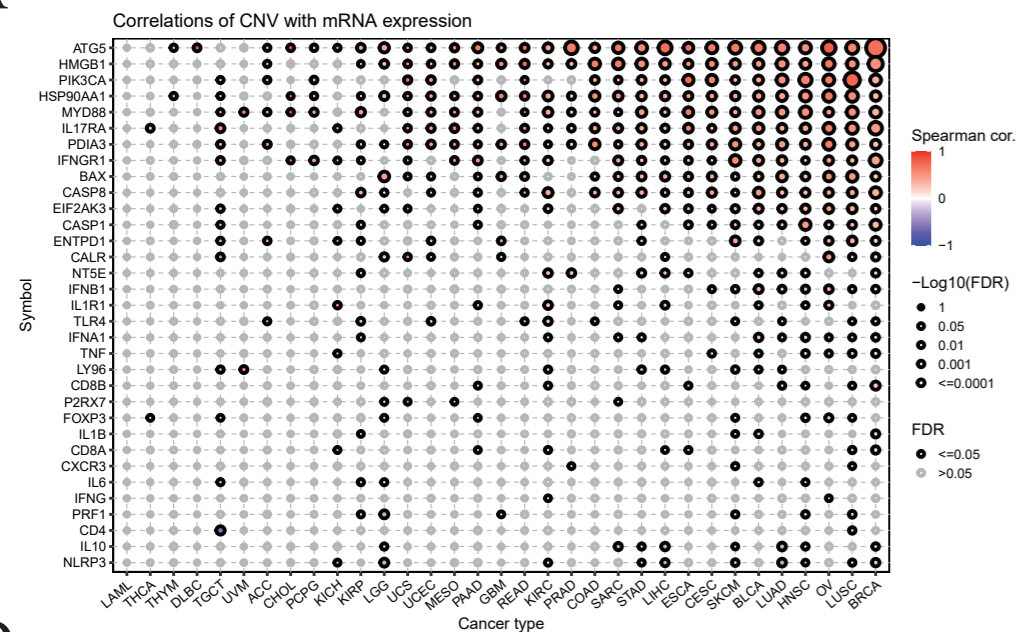

B

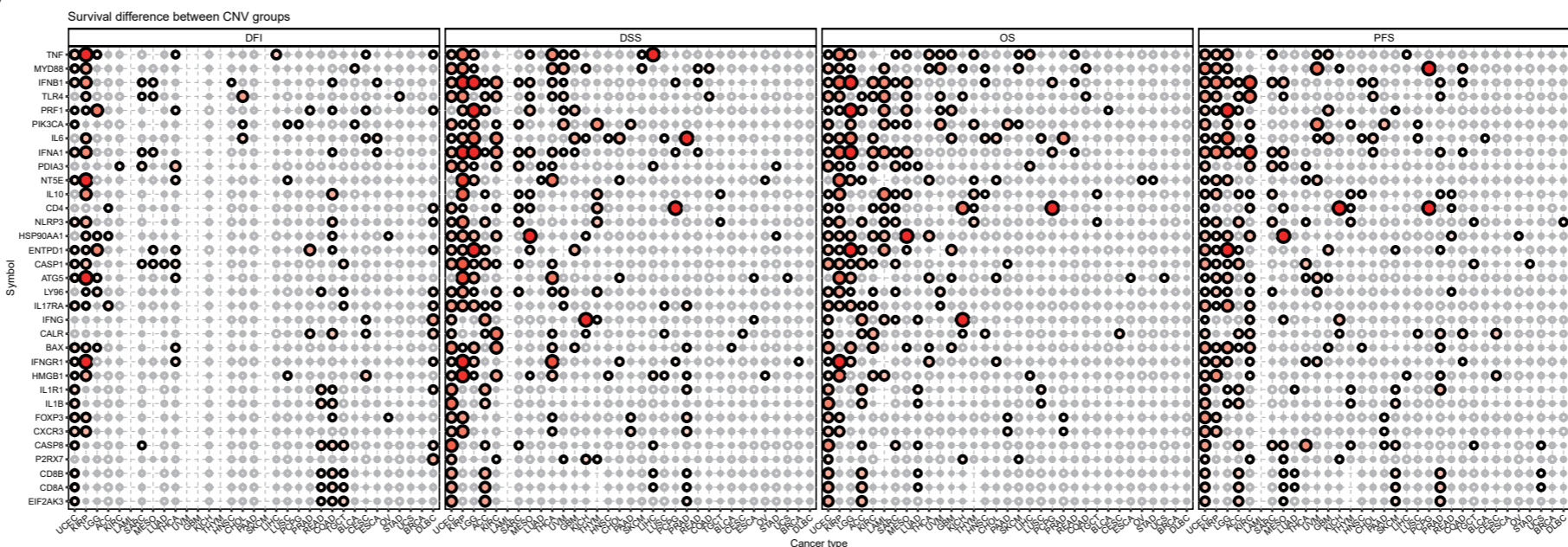

C

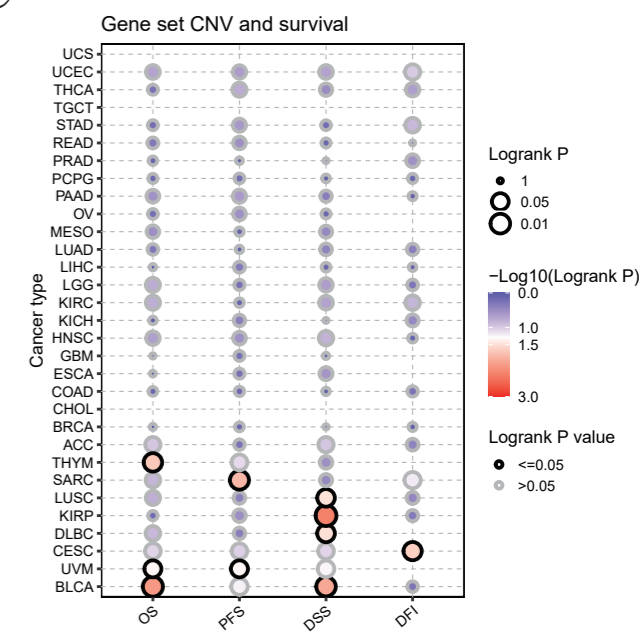

D

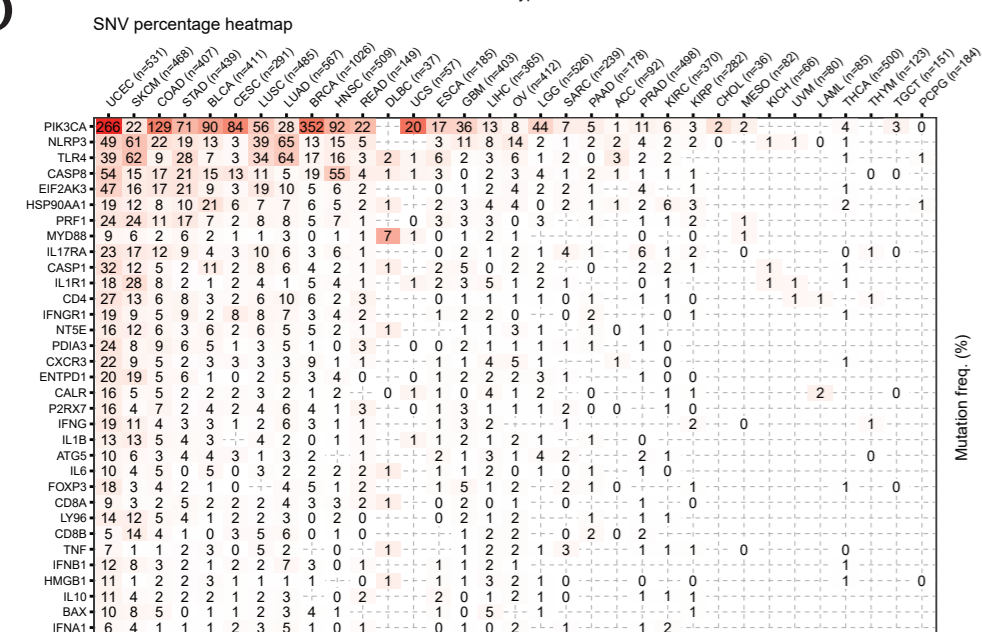

E

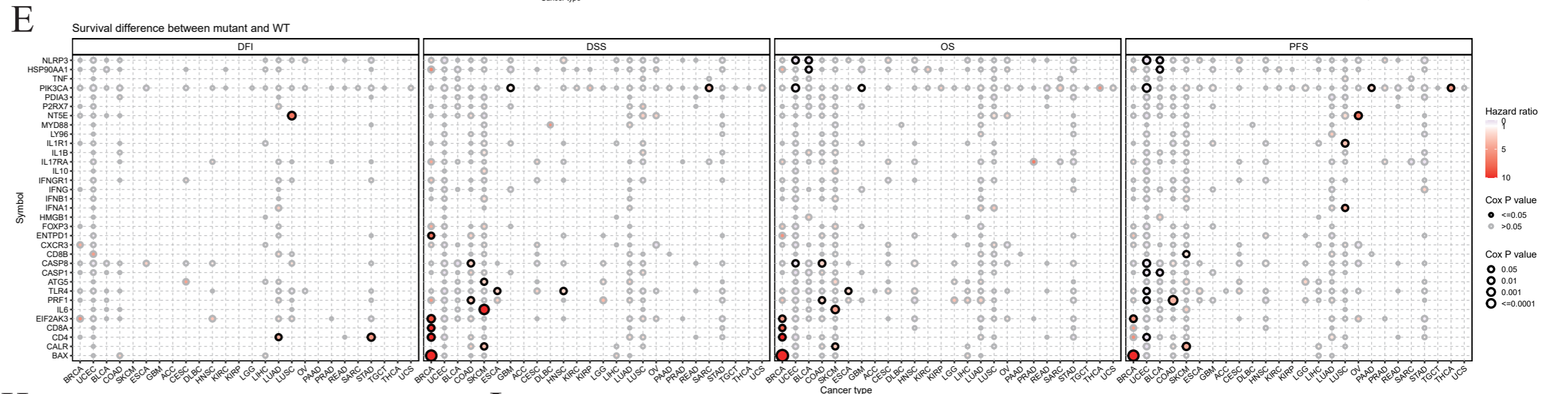

F

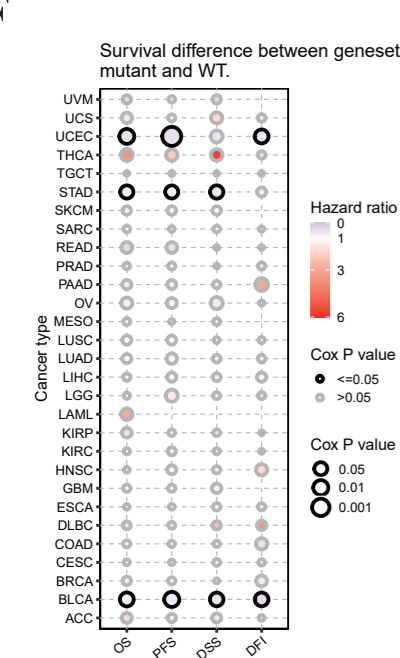

G

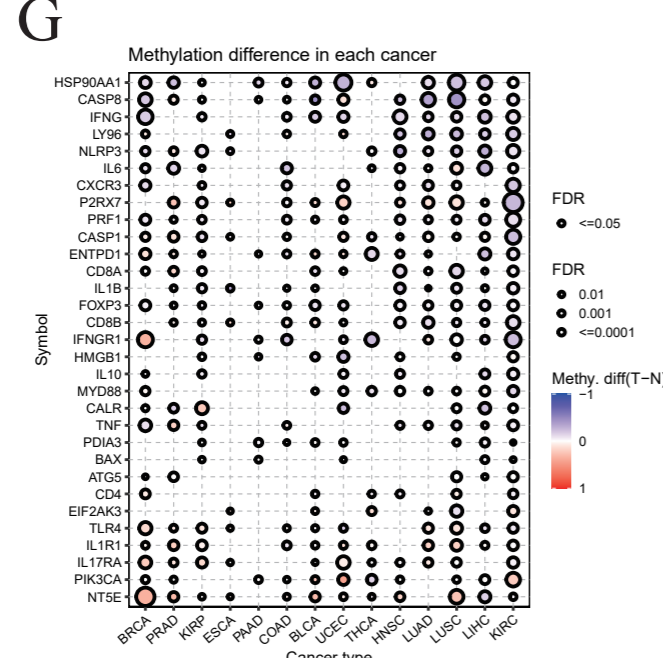

H

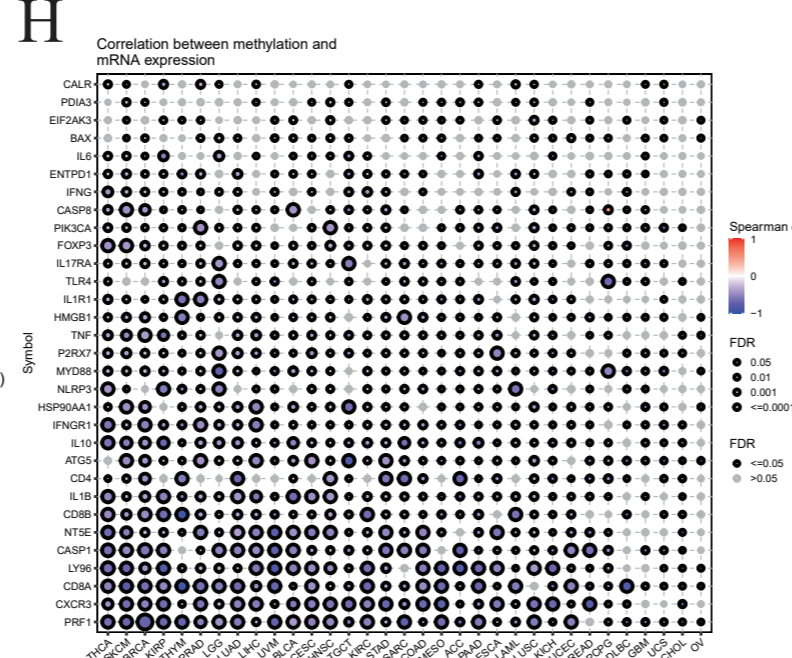

I

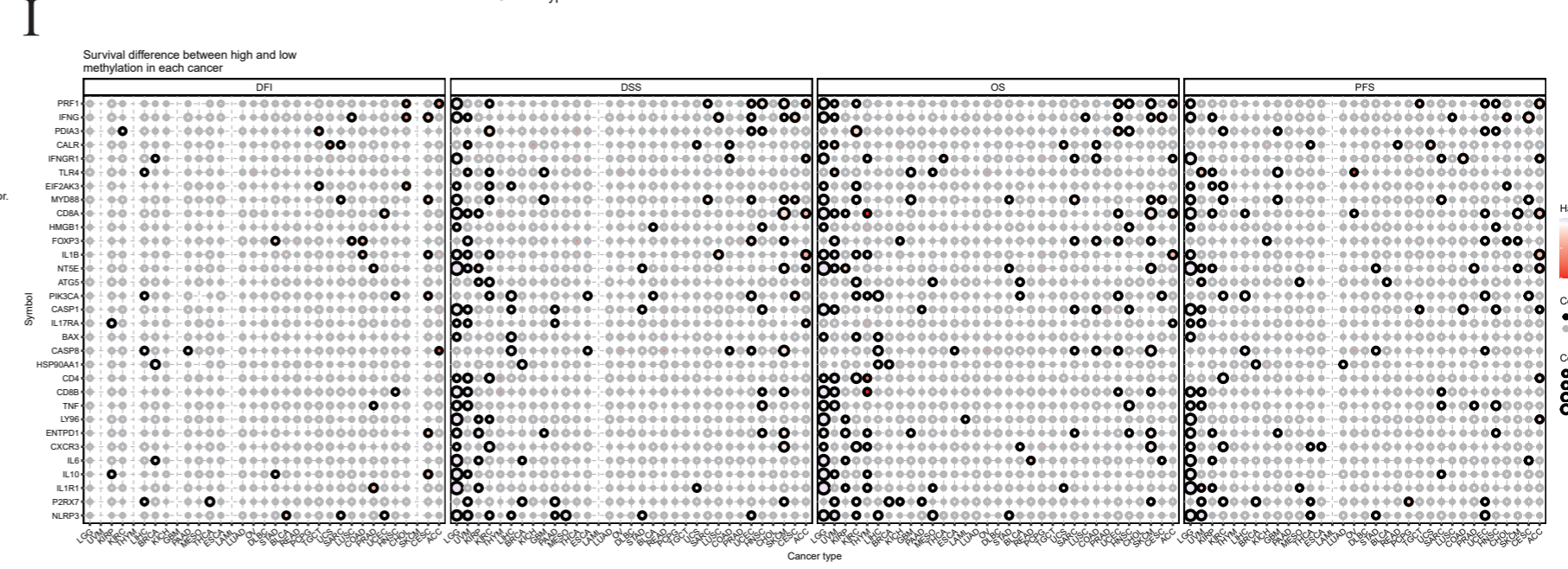

Supplement: Supplementary file 1 [file cimb-47-00812-s001.zip › cimb-3868671-supplementary/Supplementary_0930/Supplementary Figure s3.pdf]

A

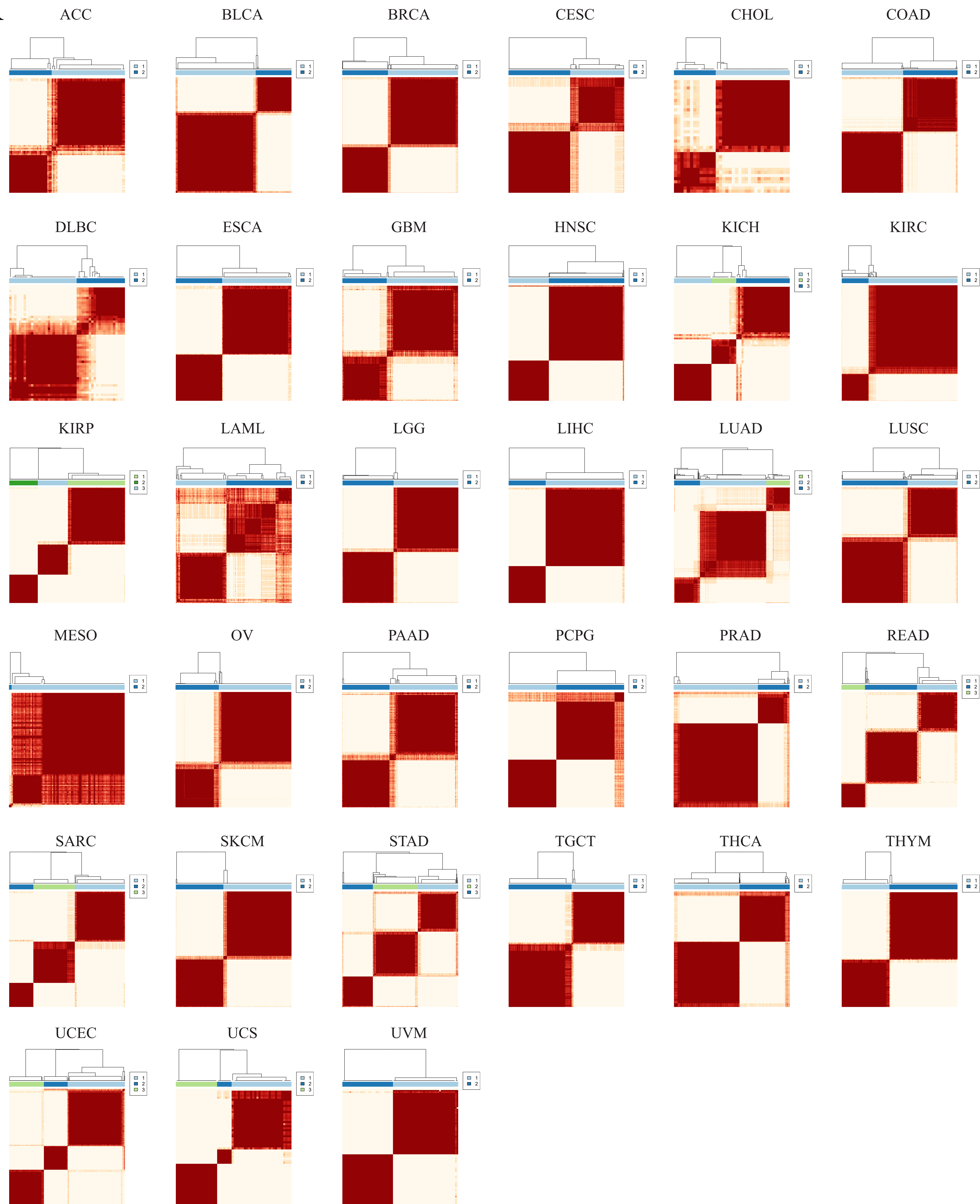

B

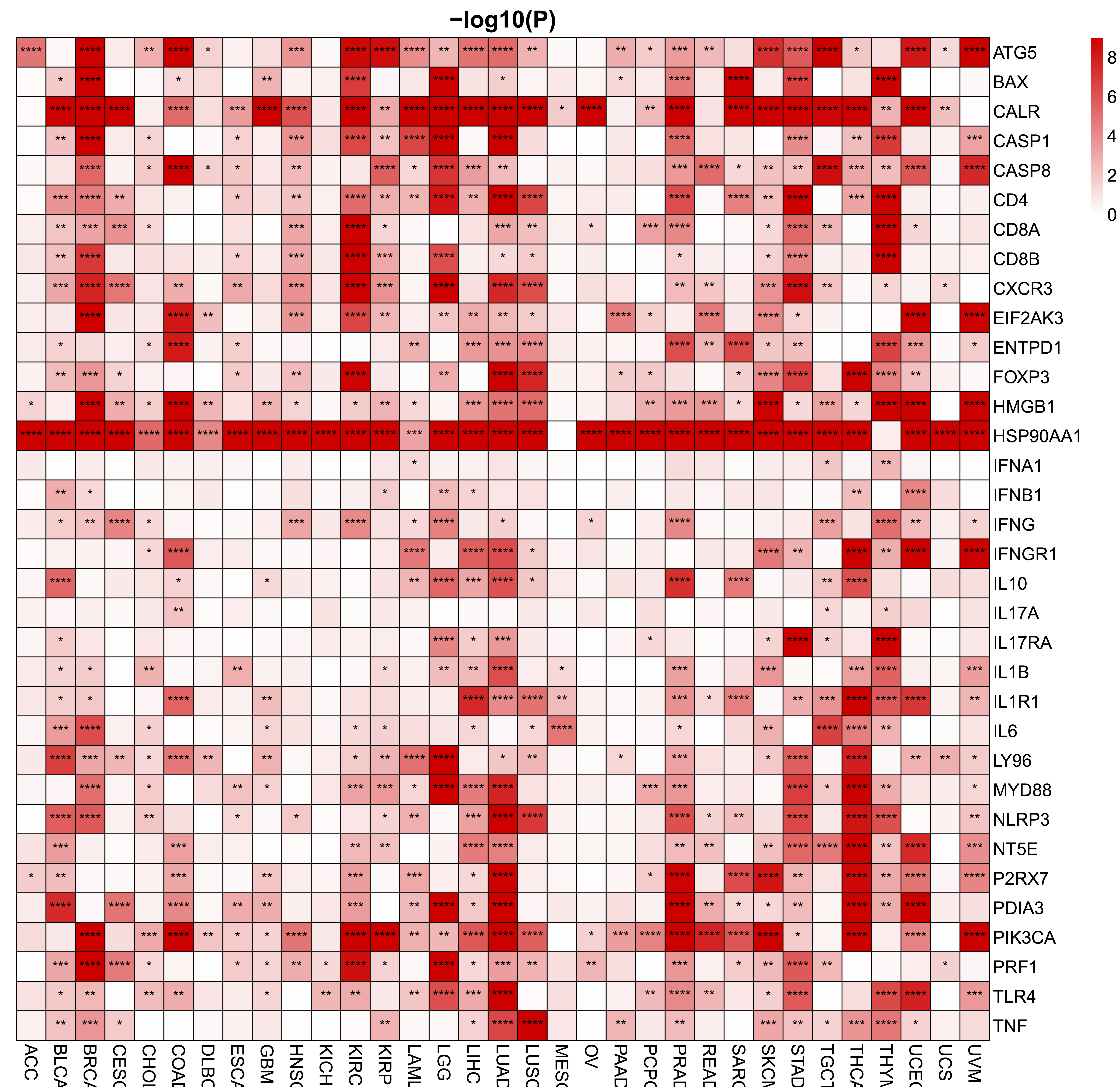

C

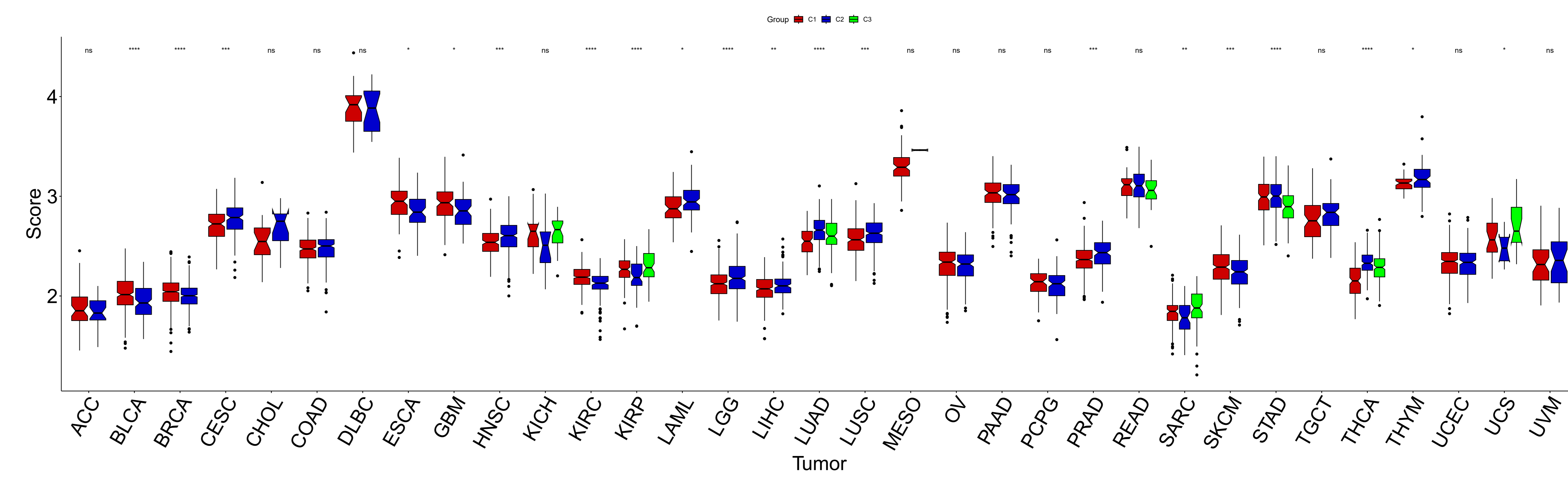

Supplement: Supplementary file 1 [file cimb-47-00812-s001.zip › cimb-3868671-supplementary/Supplementary_0930/Supplementary Figure s4.pdf]

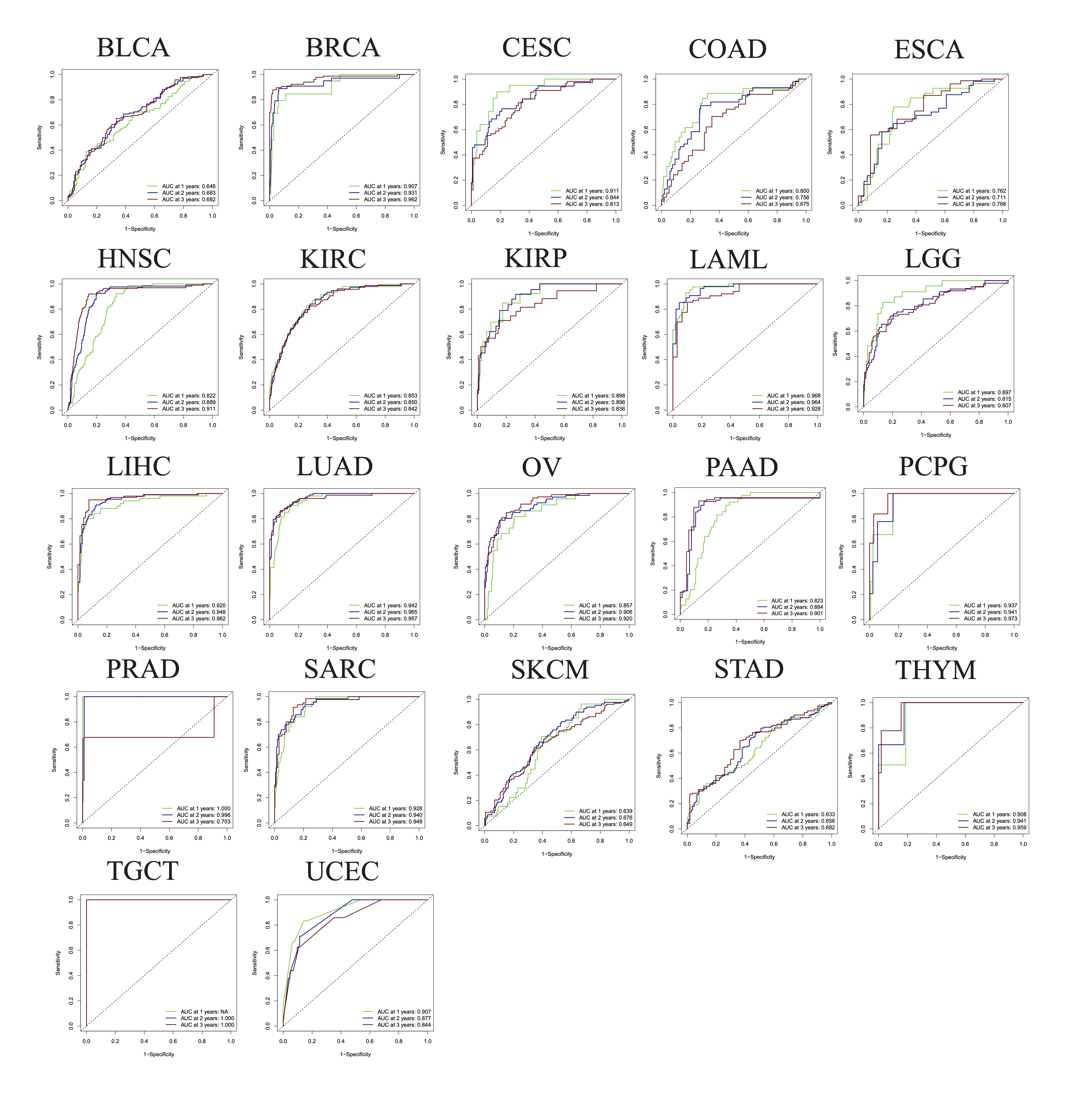

Supplement: Supplementary file 1 [file cimb-47-00812-s001.zip › cimb-3868671-supplementary/Supplementary_0930/Supplementary Figure s6-ROC.jpg]

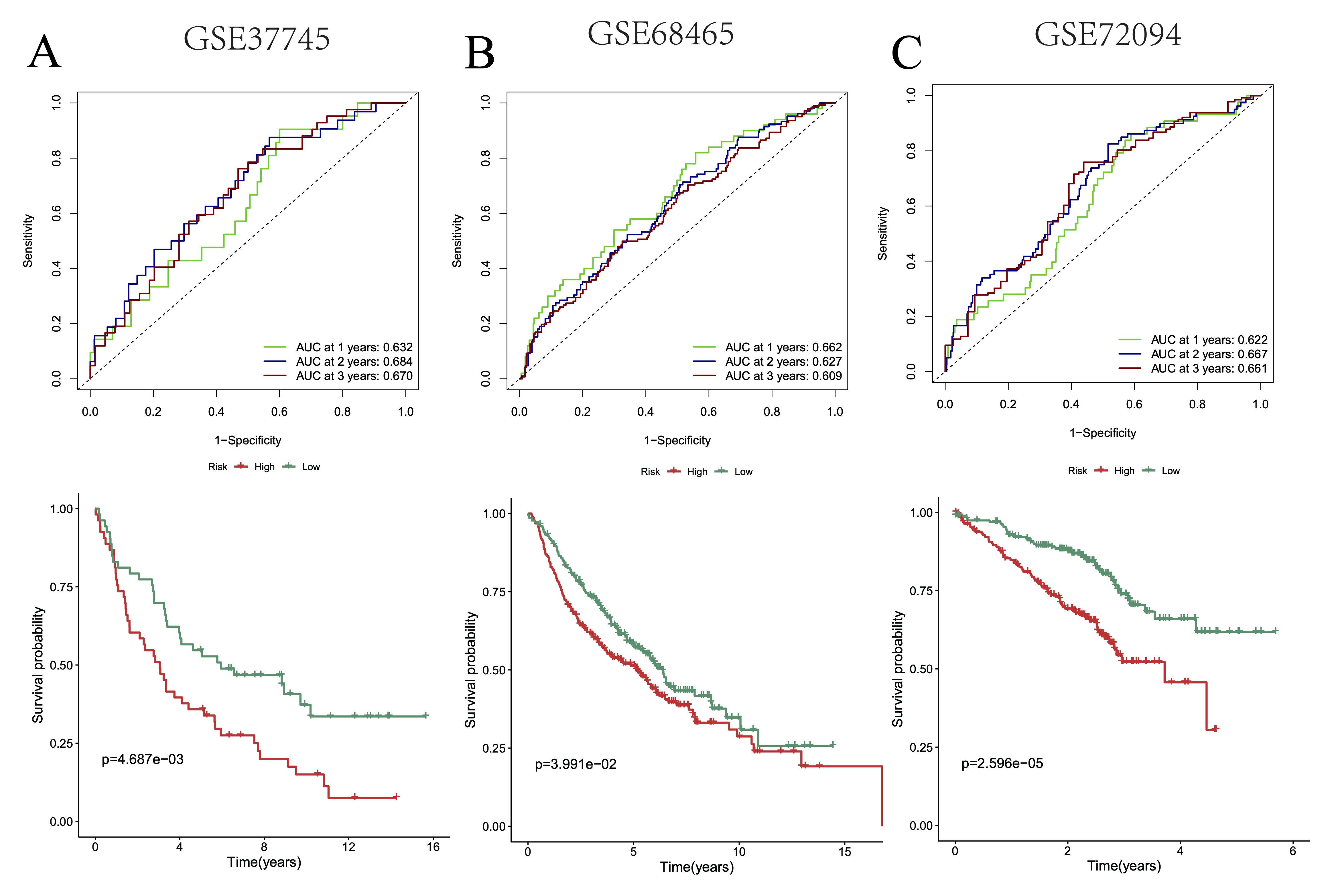

Supplement: Supplementary file 1 [file cimb-47-00812-s001.zip › cimb-3868671-supplementary/Supplementary_0930/Supplementary Figure s7-luad.jpg]

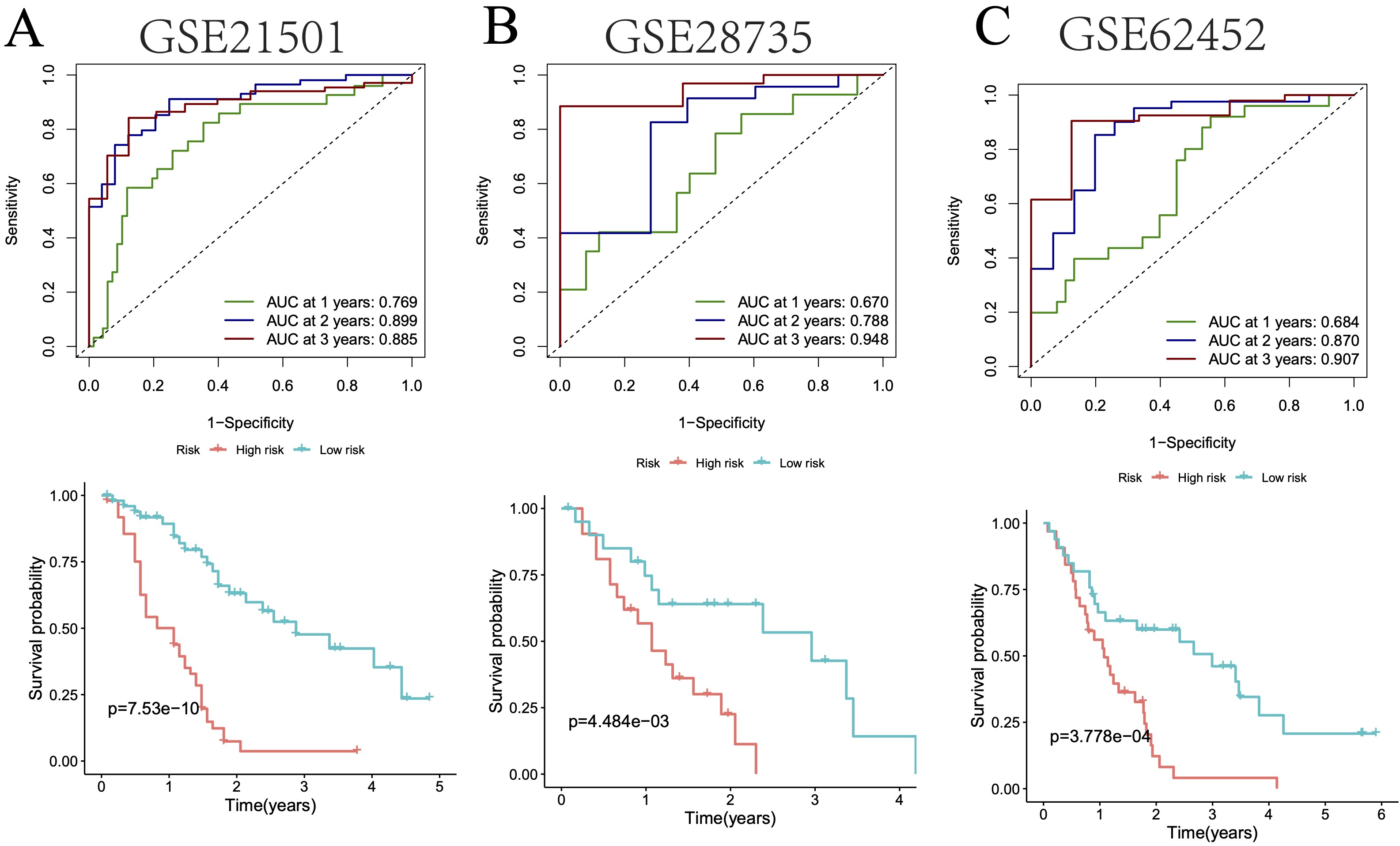

Supplement: Supplementary file 1 [file cimb-47-00812-s001.zip › cimb-3868671-supplementary/Supplementary_0930/Supplementary Figure s8-paad.jpg]
