# Supplementary material for: Constructing a Pan-Cancer Prognostic Model via Machine Learning Based on Immunogenic Cell Death Genes and Identifying NT5E as a Biomarker in Head and Neck Cancer
Source: Curr Issues Mol Biol. 2025 Oct 1;47(10):812. doi: 10.3390/cimb47100812 (PMC12564767; doi:10.3390/cimb47100812)
Supplement: Supplementary file 1 [file Supplementary Figure s14-Immunoinhibitor.pdf › cimb-3868671-supplementary/Supplementary_0930/Supplementary Figure s14-Immunoinhibitor.pdf]

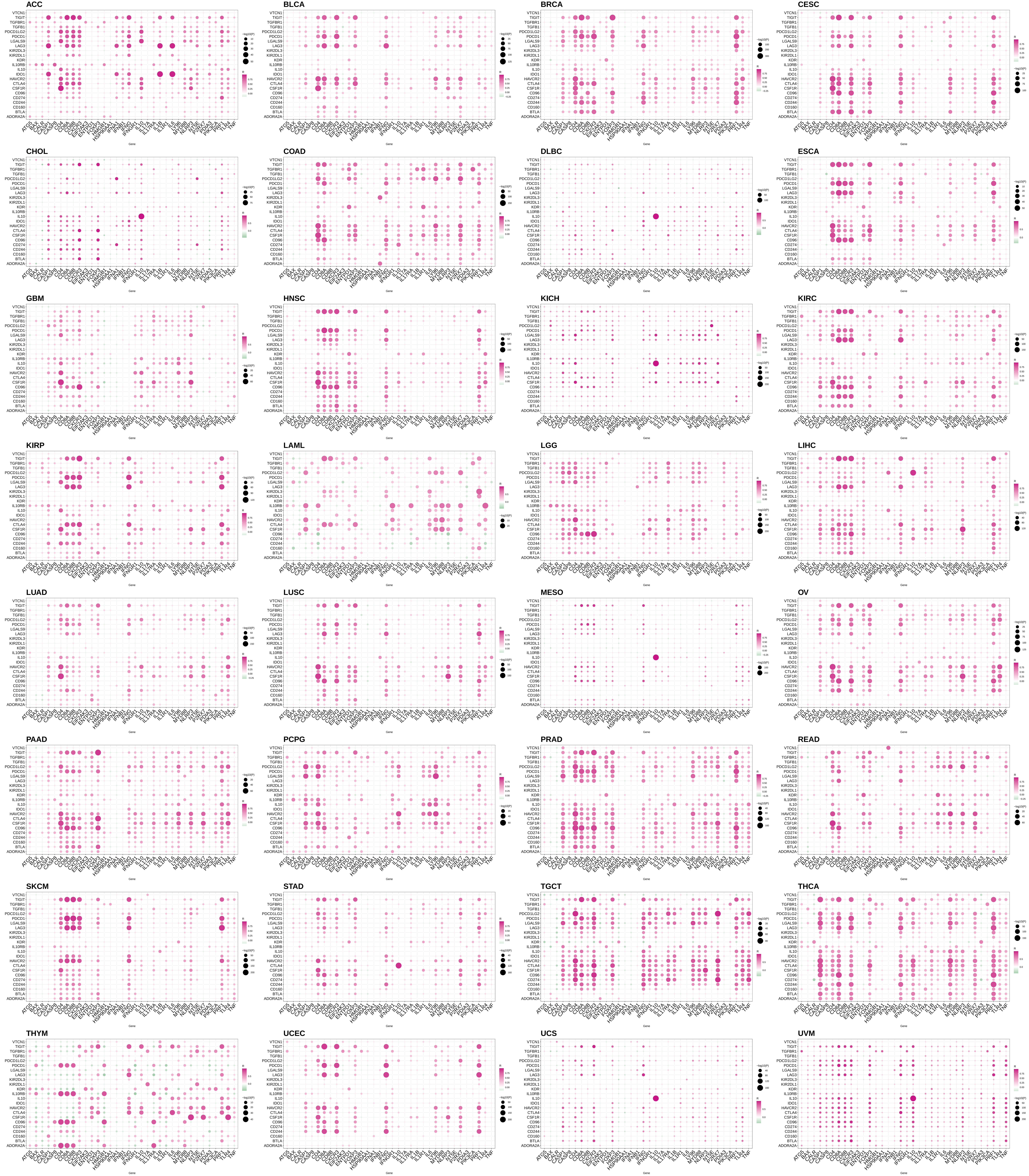
ACC
BLCA
BRCA
CESC
CHOL
COAD
DLBC
ESCA
GBM
HNSC
KICH
KIRC
KIRP
LAML
LGG
LIHC
LUAD
LUSC
MESO
OV
PAAD
PCPG
PRAD
READ
SKCM
STAD
TGCT
THCA
THYM
UCEC
UCS
UVM
VTCN1
TIGIT
TGFBR1
TGFB1
PDCD1LG2
PDCD1
LGALS9
LAG3
KIR2DL3
KIR2DL1
KDR
IL10RB
IL10
IDO1
HAVCR2
CTLA4
CSF1R
CD96
CD274
CD244
CD160
BTLA
ADORA2A
ATG5
BAX
CALR
CASP8
CD4
CD8A
CD8B
CXCR3
EIF2AK3
ENTPD1
FOXP3
HMGB1
HSP90AA1
IFNA1
IFNB1
IFNG
IFNGR1
IL10
IL17A
IL17RA
IL1B
IL1R1
IL6
LY96
MYD88
NLRP3
NT5E
P2RX7
PDIA3
PIK3CA
PRF1
TLR4
TNF
Gene
R
-log10(P)
