# Supplementary material for: Constructing a Pan-Cancer Prognostic Model via Machine Learning Based on Immunogenic Cell Death Genes and Identifying NT5E as a Biomarker in Head and Neck Cancer
Source: Curr Issues Mol Biol. 2025 Oct 1;47(10):812. doi: 10.3390/cimb47100812 (PMC12564767; doi:10.3390/cimb47100812)
Supplement: Supplementary file 1 [file Supplementary Figure s17-Chemokine.pdf › cimb-3868671-supplementary/Supplementary_0930/Supplementary Figure s17-Chemokine.pdf]

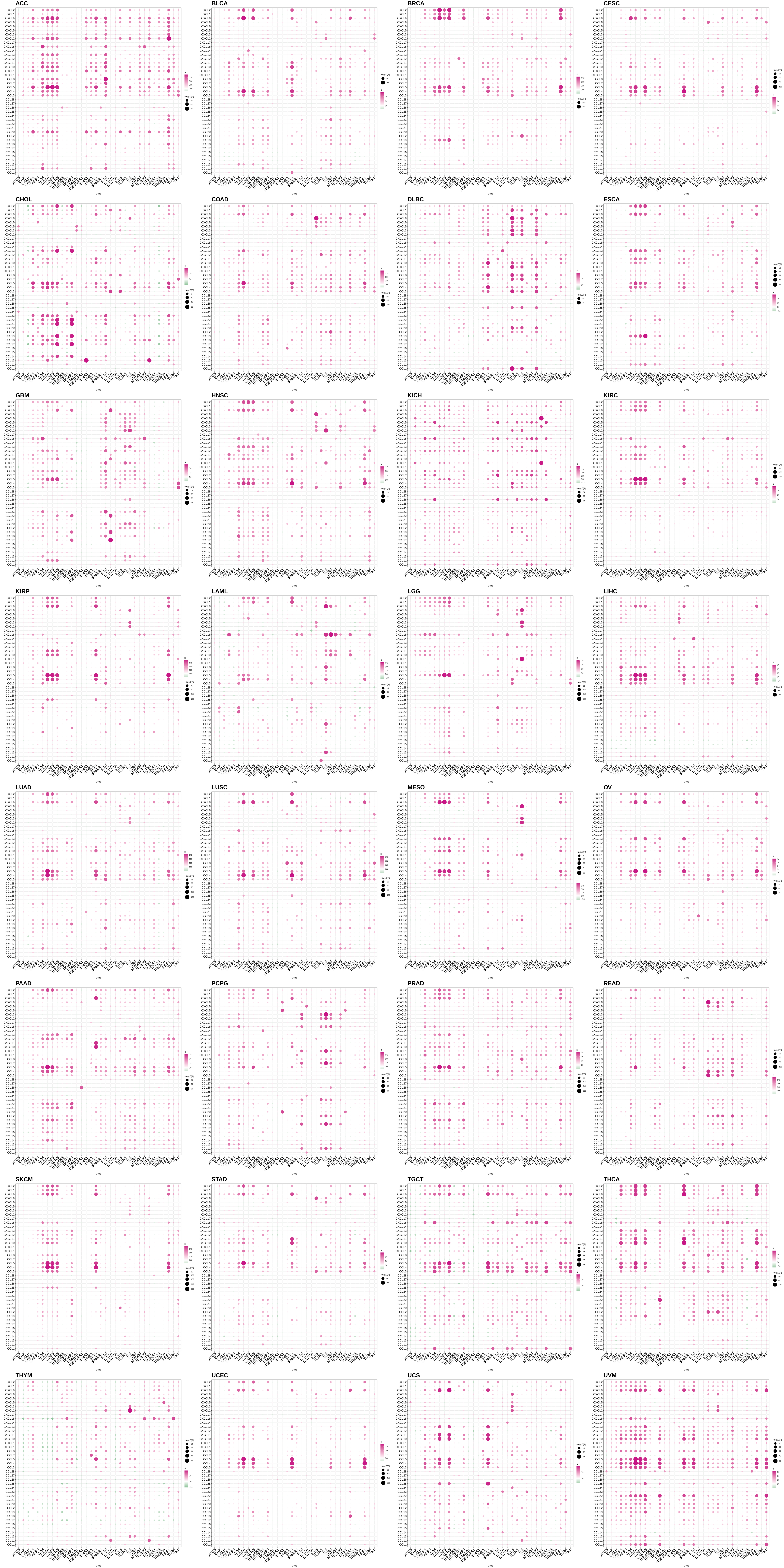
ACC
BLCA
BRCA
CESC
CHOL
COAD
DLBC
ESCA
GBM
HNSC
KICH
KIRC
KIRP
LAML
LGG
LIHC
LUAD
LUSC
MESO
OV
PAAD
PCPG
PRAD
READ
SKCM
STAD
TGCT
THCA
THYM
UCEC
UCS
UVM
