# Supplementary material for: Constructing a Pan-Cancer Prognostic Model via Machine Learning Based on Immunogenic Cell Death Genes and Identifying NT5E as a Biomarker in Head and Neck Cancer
Source: Curr Issues Mol Biol. 2025 Oct 1;47(10):812. doi: 10.3390/cimb47100812 (PMC12564767; doi:10.3390/cimb47100812)
Supplement: Supplementary file 1 [file Supplementary Figure s5-out_full.pdf › cimb-3868671-supplementary/Supplementary_0930/Supplementary Figure s5-out_full.pdf]

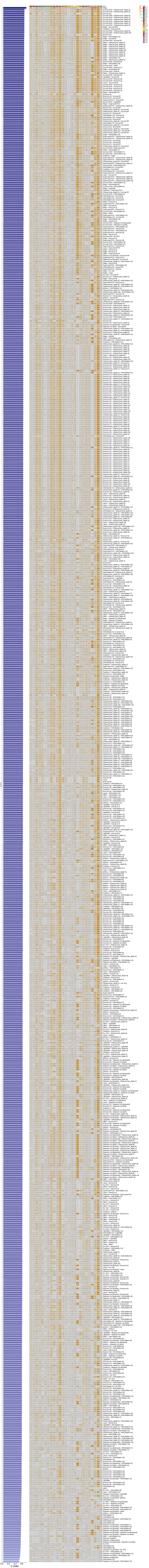
C.index
0.0
0.2
0.4
0.6
